# Effect of Physician-Delivered COVID-19 Public Health Messages and Messages Acknowledging Racial Inequity on Black and White Adults’ Knowledge, Beliefs, and Practices Related to COVID-19: A Randomized Clinical Trial

**DOI:** 10.1001/jamanetworkopen.2021.17115

**Published:** 2021-07-14

**Authors:** Carlos Torres, Lucy Ogbu-Nwobodo, Marcella Alsan, Fatima Cody Stanford, Abhijit Banerjee, Emily Breza, Arun G. Chandrasekhar, Sarah Eichmeyer, Mohit Karnani, Tristan Loisel, Paul Goldsmith-Pinkham, Benjamin A. Olken, Pierre-Luc Vautrey, Erica Warner, Esther Duflo

**Affiliations:** 1 Harvard Medical School, Boston, Massachusetts; 2 Department of Pediatrics–General Pediatrics, Massachusetts General Hospital for Children, Boston; 3 Department of Psychiatry, Massachusetts General Hospital, Boston; 4 Department of Psychiatry, McLean Hospital, Belmont, Massachusetts; 5 Harvard Kennedy School of Government, Cambridge, Massachusetts; 6 Department of Medicine–Neuroendocrine Unit, Department of Pediatrics–Endocrinology, Massachusetts General Hospital, Boston; 7 Department of Economics, Massachusetts Institute of Technology, Cambridge; 8 Department of Economics, Harvard University, Cambridge, Massachusetts; 9 Department of Economics, Stanford University, Stanford, California; 10 Department of Economics, Ludwig Maximilian University of Munich, Munich, Germany; 11 Paris School of Economics, Paris, France; 12 Yale School of Management, New Haven, Connecticut; 13 Clinical Translational Epidemiology Unit, Massachusetts General Hospital, Boston

## Abstract

**Importance:**

Social distancing is critical to the control of COVID-19, which has disproportionately affected the Black community. Physician-delivered messages may increase adherence to these behaviors.

**Objectives:**

To determine whether messages delivered by physicians improve COVID-19 knowledge and preventive behaviors and to assess the differential effectiveness of messages tailored to the Black community.

**Design, Setting, and Participants:**

This randomized clinical trial of self-identified White and Black adults with less than a college education was conducted from August 7 to September 6, 2020. Of 44 743 volunteers screened, 30 174 were eligible, 5534 did not consent or failed attention checks, and 4163 left the survey before randomization. The final sample had 20 460 individuals (participation rate, 68%). Participants were randomly assigned to receive video messages on COVID-19 or other health topics.

**Interventions:**

Participants saw video messages delivered either by a Black or a White study physician. In the control groups, participants saw 3 placebo videos with generic health topics. In the treatment group, they saw 3 videos on COVID-19, recorded by several physicians of varied age, gender, and race. Video 1 discussed common symptoms. Video 2 highlighted case numbers; in one group, the unequal burden of the disease by race was discussed. Video 3 described US Centers for Disease Control and Prevention social distancing guidelines. Participants in both the control and intervention groups were also randomly assigned to see 1 of 2 American Medical Association statements, one on structural racism and the other on drug price transparency.

**Main Outcomes and Measures:**

Knowledge, beliefs, and practices related to COVID-19, demand for information, willingness to pay for masks, and self-reported behavior.

**Results:**

Overall, 18 223 participants (9168 Black; 9055 White) completed the survey (9980 [55.9%] women, mean [SD] age, 40.2 [17.8] years). Overall, 6303 Black participants (34.6%) and 7842 White participants (43.0%) were assigned to the intervention group, and 1576 Black participants (8.6%) and 1968 White participants (10.8%) were assigned to the control group. Compared with the control group, the intervention group had smaller gaps in COVID-19 knowledge (incidence rate ratio [IRR], 0.89 [95% CI, 0.87-0.91]) and greater demand for COVID-19 information (IRR, 1.05 [95% CI, 1.01-1.11]), willingness to pay for a mask (difference, $0.50 [95% CI, $0.15-$0.85]). Self-reported safety behavior improved, although the difference was not statistically significant (IRR, 0.96 [95% CI, 0.92-1.01]; *P* = .08). Effects did not differ by race (*F* = 0.0112; *P* > .99) or in different intervention groups (*F* = 0.324; *P* > .99).

**Conclusions and Relevance:**

In this study, a physician messaging campaign was effective in increasing COVID-19 knowledge, information-seeking, and self-reported protective behaviors among diverse groups. Studies implemented at scale are needed to confirm clinical importance.

**Trial Registration:**

ClinicalTrials.gov Identifier: NCT04502056

## Key Points

**Question:**

Do messages delivered by physicians increase COVID-19 knowledge and improve preventive behaviors among White and Black individuals?

**Findings:**

In this randomized clinical trial of 18 223 White and Black adults, a message delivered by a physician increased COVID-19 knowledge and shifted information-seeking and self-protective behaviors. Effects did not differ by race, and tailoring messages to specific communities did not exhibit a differential effect on knowledge or individual behavior.

**Meaning:**

These findings suggest that physician messaging campaigns may be effective in persuading members of society from a broad range of backgrounds to seek information and adopt preventive behaviors to combat COVID-19.

## Teaser

This randomized clinical trial evaluates whether messages delivered by physicians improve COVID-19 knowledge and preventive behaviors and assesses the differential effectiveness of messages tailored to the Black community.

## Introduction

Physical distancing and mask wearing remain essential to the control of COVID-19, yet vigilance has decreased over time.^1^ To address fatigue, health care professionals have used social media to spread public health messages.^2^ There is evidence that these messages improve knowledge, but there are less data on whether they change behavior.^3^

Black US residents have been disproportionately affected by the pandemic.^4^ This reflects the cumulative impact of systemic racism, acknowledged as a public health threat by the American Medical Association (AMA) in a June 2020 statement.^5^ This raises the question on whether the effectiveness of public health messages regarding COVID-19 would be enhanced if tailored to the Black community. The focus of this study was to identify whether messages delivered by physicians increase COVID-19 knowledge and improve preventive behaviors for White and Black individuals and to assess whether various ways of increasing the relevance of messages to the Black community (ie, physician race, AMA acknowledgments of racial injustices, or information about the disproportionate burden of COVID-19 on the Black community) affects their impact on both White and Black participants.

## Methods

### Trial Design and Oversight

**Figure 1.**
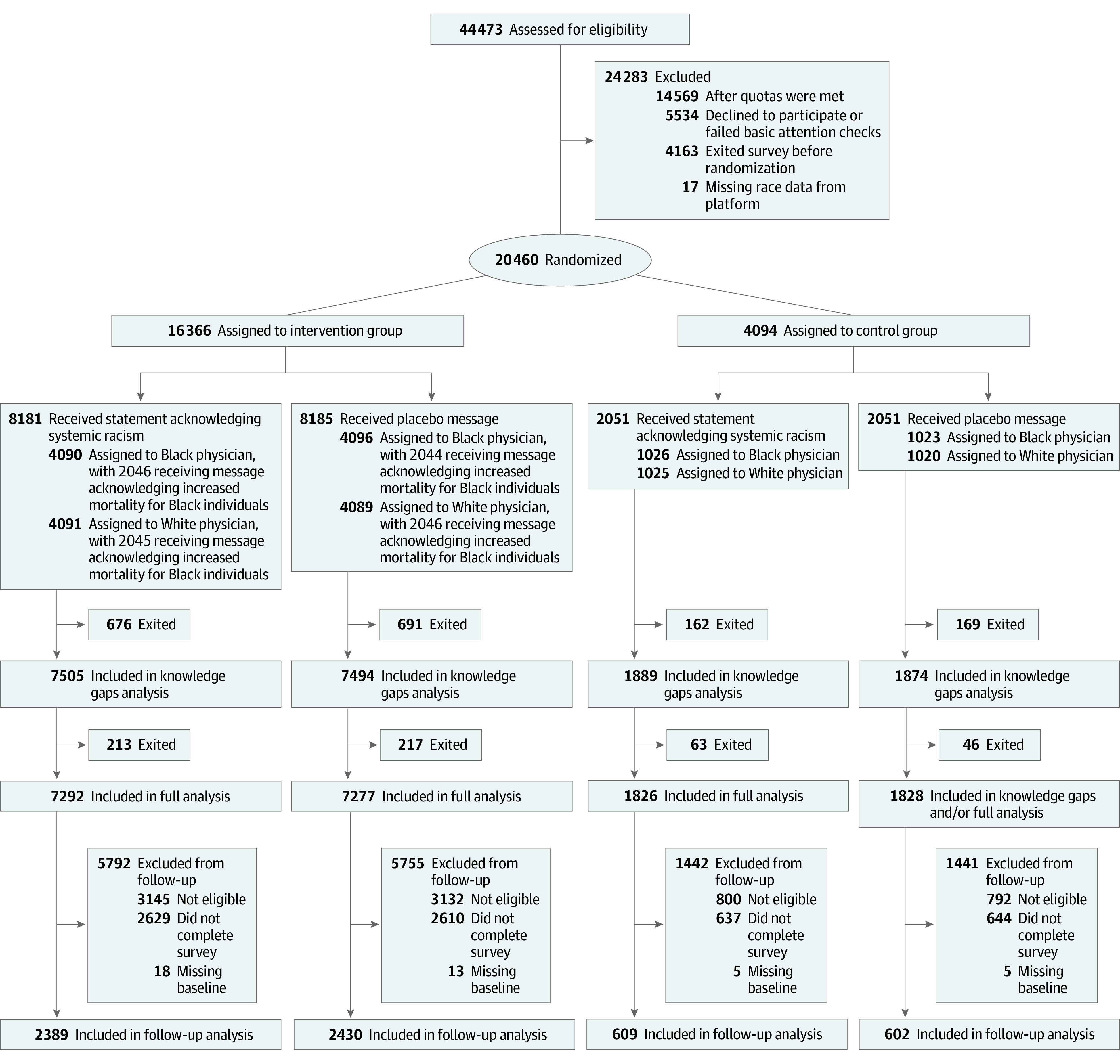
Enrollment and Randomization of Participants

The trial flowchart (Figure 1; eFigure 1 in Supplement 1) describes the factorial design and the allocation of participants to each intervention arm. The design was approved by the ethical review boards of Massachusetts Institute of Technology (MIT) and Stanford, with Massachusetts General Hospital, Yale, and Harvard ceding authority to MIT. All participants provided written informed consent. The trial and the outcomes were registered on ClinicalTrials.gov (NCT04502056). Planned analyses were published on the American Economic Association trial registry (AEARCTR-0006177). The pre-analysis plan and institutional review board–approved protocol are available in Supplement 2. This study followed the Consolidated Standards of Reporting Trials (CONSORT) and American Association for Public Research (AAPOR) reporting guidelines.

### Participants

Individuals were recruited online throughout the United States by the survey company Lucid from August 7, 2020, to September 6, 2020. Lucid recruits survey participants by advertising surveys to third-party suppliers, including double opt-in panels, publishing networks, social media, and other types of online communities. Participants aged 18 years or older, self-identifying as White or Black, and without a college degree were eligible. We focused on these 2 groups because we were interested in tailoring messages toward the Black community as well as the reaction of the White community to the tailoring of those messages. Latinx individuals were included in our previous study,^3^ along with specific tailoring toward this community. Recruitment used quotas to match the 2018 population estimates by age, sex, and race issued by the US Census Bureau.^6^

### Interventions

#### Intervention vs Control Messages

After reading the informed consent form (approved by the institutional review board) (eAppendix 1 in Supplement 1) and agreeing to participate, all individuals answered sociodemographic questions, saw 3 videos, and then completed the outcome survey questions. In the control groups, participants saw 3 placebo videos with generic health topics, including fitness guidelines, recommended sugar intake, and the importance of adequate sleep. In the treatment group, they saw 3 videos on COVID-19, recorded by several physicians of varied age, gender, and race. Participants in each group (placebo and intervention) saw video messages delivered either by a Black or a White study physician (including L.O.-N., M.A., F.C.S., and E.W.).

Video 1 defined COVID-19 and discussed common symptoms associated with COVID-19 as well as asymptomatic transmission. Video 2 reminded the viewer that COVID-19 was actively circulating in the United States. Video 3 described the Centers for Disease Control and Prevention social distancing guidelines (complete scripts appear in eAppendix 1 in Supplement 1).

#### Unequal Burden of COVID-19

Video 2 in the COVID-19 intervention had 2 randomized variants. Script 1 emphasized the number of new cases in the week of July 6, 2020. Script 2 added that, controlling for age, Black individuals were 3 times as likely to become infected as White individuals and 4 times as likely to die from it. These 2 variants of video 2 were cross-randomized with the intervention.

#### AMA Antiracism or Placebo Statement

At the beginning of the study, all participants saw a video of an actor delivering the script of a public statement by the AMA. The AMA antiracism statement, issued on June 7, 2020, “recognizes that racism in its systemic, structural, institutional, and interpersonal forms is an urgent threat to public health, the advancement of health equity, and a barrier to excellence in the delivery of medical care.”^5^ The AMA placebo intervention was an AMA statement on drug pricing.^7^ The race and gender of the person reading the statement were randomized to each recipient.

### Outcomes

Most outcomes were measured online immediately following the intervention or the placebo. The prespecified primary outcomes were knowledge, beliefs, and practices related to COVID-19, measured immediately after the intervention; intended behavior, measured immediately after the intervention; and knowledge and behavior, measured a few days after the intervention.

eAppendix 2 in Supplement 1 describes the outcome measurement in detail. Primary outcomes presented in the main text include 5 outcomes. First, knowledge gap, which measures knowledge and beliefs. Participants were asked to identify ways to prevent COVID-19 spread and identify 4 common symptoms. The knowledge gap outcome is an integer that can have values from 0 (no error) to 10 (10 errors). Second, information seeking was measured by offering participants the option of requesting additional information on COVID-19–related resources by clicking on up to 5 links that included more content. We measured information-seeking behavior as the number of links in which participants expressed interest, a count variable between 0 (lowest information-seeking behavior) and 5 (greatest information-seeking behavior). Third, self-reported safety behavior was measured a few days after the initial intervention among a subsample that was eligible for follow-up and could be tracked. Participants were asked about how often they engaged in 4 behaviors of interest: (1) whether they wore a mask indoors; (2) whether they wore a mask outdoors; (3) whether they washed their hands; and (4) whether they followed social distancing guidelines. The safety gap index had values of 0 (if a participant reported that they always practiced the 4 behaviors of interest) to 4 (participant reported that they practiced none of the behaviors). Fourth, at the end of the survey, each participant was asked the price they would be willing to pay for high-quality masks. Each participant was entered into a draw to receive either a coupon for masks or a gift card to an online retailer. When a participant was selected by the draw, a price was then randomly drawn for the coupon. If their reported willingness to pay was greater than the price, they would receive the masks; otherwise, they would receive the gift card. Therefore, it was in participants’ best interest to report their true willingness to pay for the masks. This type of procedure has been shown to lead to truthful reporting.^8^

We collected data on 3 secondary outcomes specified in our pre-analysis plan (Supplement 2). First, we asked participants to report their judgment of how well federal and state policies balanced opening the economy and limiting the health impacts of COVID-19. Second, we measured how participants prioritized COVID-19 protection vs other issues by asking the participants how they would want to allocate a donation of $1000 (which the research team would fund) between 2 charities: Give a Mask or the Alzheimer Association. Third, we asked whether they would prioritize a COVID-19 relief donation to a Black-focused charity (the BET COVID-19 relief fund) or a general charity (the Give Directly Project 100+ relief program).

### Randomization

Randomization into intervention vs control and between interventions was stratified according to sex, age (≥45 years), race, and self-identified partisan affiliation (Republican vs other affiliation). The flowchart (Figure 1) summarizes the randomization; a more detailed version is available as eFigure 1 in Supplement 1.

Participants were first randomized into the AMA antiracism or placebo statements, with equal probability. They were then randomly assigned to intervention or control. One of 5 participants was assigned to control; the remainder were assigned to an intervention.

Within each group, participants were randomized into Black or White physician groups with equal probability. Intervention participants were further randomized, with equal probability, into 1 the 2 arms for video 2: they either received the information about the unequal burden of disease or did not. Randomization was performed using the Qualtrics platform, using a randomizer block within each stratum with the option to evenly present elements.

### Statistical Analysis

We determined that a sample of 20 000 individuals (10 000 Black and 10 000 White) would provide 85% power to detect effect sizes of 0.05 SDs for intervention relative to control and for effects of specific variations in message content. These are small effect sizes that would justify scale up of this inexpensive intervention.

The analysis was performed by original assigned group, and it included all participants who completed the survey. Multivariable regression models include the stratifying variables (age × sex × race × Republican identity dummies).

#### Effect of Any Video Message Intervention Relative to Control

To analyze the effect of seeing any video message on the knowledge gap, information-seeking behavior, and safety behavior outcomes, we fit the following negative binomial regression model for the count outcome:

log(μ*_i_*) = β_0_ + β_1_intervention*_i_* + β^T^_2_stratum*_i_*,

where μ*_i_* is the estimated mean outcome value (knowledge gap count, count of demanded links, or safety behavior count), intervention*_i_* is an indicator that equals 1 if the individual received the intervention videos and 0 if they received the placebo videos, and stratum*_i_* is a vector of indicator variables. Similar models are estimated for binary regressions (using the logistic regression equation) and continuous variable (using ordinary least squares) (eAppendix 2 in Supplement 1). Because there were multiple outcomes, we provide *P* values and q values adjusted for false discovery rates.

#### Effect of Variation in the Message Framing

To analyze the impact of different arms, we fit a negative binomial regression model to the count data:

log(μ*_i_*) = β_0_ + β_1_Black physician*_i_* + β_2_AMA antiracism*_i_* + β_3_intervention*_i_* + (β_4_Black physician*_i_* × intervention*_i_*) + (β_5_AMA antracism*_i_* × intervention*_i_*) + (β_6_mortality difference*_i_* × intervention*_i_*) + β^T^_7_stratum*_i_*,

where Black physician*_i_* is an indicator that equals 1 if the physician was a Black individual and 0 otherwise; AMA antiracism*_i_* is an indicator for the AMA statement featuring the antiracism message (rather than the drug pricing message); mortality difference*_i_* is an indicator for video 2 mentioning the excess mortality from COVID-19. Similar specifications are fitted with logistic regression for binary variables and ordinary least squares for continuous variables.

To address possible bias stemming from nonrandom attrition for the follow-up survey, we weighted the follow-up data using Hainmueller entropy weights,^9^ which ensures that the observed baseline characteristics of the follow-up sample matches the original sample as closely as possible (eAppendix 2 in Supplement 1). eAppendix 3 in Supplement 1 describes further robustness checks and subgroups analysis.

Analyses were performed using R version 4.0.3 (R Project for Statistical Computing). Statistical significance was set at *P* < .05, and all tests were 2-tailed.

## Results

### Trial Sample

The trial sample was enrolled from August 7 through September 6, 2020. Of 44 743 screened, 30 174 were eligible for participation, 5534 individuals did not consent or failed 2 simple attention checks, 4163 left the survey before randomization, and 17 were excluded from the analysis due to unknown race or multiple survey completion. The final sample at randomization had 20 460 individuals, for a participation rate of 68%. After attrition, 18 223 individuals were included in the study.

Summary statistics (Table 1) were computed on the sample that was randomized and that completed all main baseline variables. Our sample included 9880 (55.9%) women. The mean (SD) age was 40.2 (17.8) years, 4206 (23.8%) reported household incomes greater than $60 000, 4228 Black participants (53.7%) and 2749 White participants (28.0%) identified as members of the Democratic party, 12 106 (68.4%) reported always wearing a mask indoors (outside the home), and 5517 (31.2%) reported always wearing a mask when outdoors. Black participants were twice as likely as White participants to report always wearing a mask outdoors (3513 [44.6%] vs 2004 [20.4%]) and were also more likely to report practicing hand hygiene (5084 [64.5%] vs 5695 [58.1%]) and physical distancing (4587 [58.2%] vs 4874 [49.7%]). Baseline covariates were balanced between intervention and control at all stages (eAppendix 5 and eTable 1A in Supplement 1).

**Table 1.** Summary of Participant Characteristics^a^

Variable	Respondents, No. (%)								
	Full sample			Intervention group			Control group		
	All (N = 17 689)	Black (n = 7879)	White (n = 9810)	All (n = 14 145)	Black (n = 6303)	White (n = 7842)	All (n = 3544)	Black (n = 1576)	White (n = 1968)
Age, mean (SD), y	40.22 (17.81)	34.12 (15.48)	45.12 (18.04)	40.20 (17.83)	34.15 (15.48)	45.07 (18.09)	40.30 (17.73)	34.00 (15.47)	45.35 (17.81)
Region									
Northeast	3024 (17.1)	1187 (15.1)	1837 (18.7)	2424 (17.1)	952 (15.1)	1472 (18.8)	600 (16.9)	235 (14.9)	365 (18.5)
Midwest	3884 (22.0)	1494 (19.0)	2390 (24.4)	3114 (22.0)	1207 (19.1)	1907 (24.3)	770 (21.7)	287 (18.2)	483 (24.5)
South	8046 (45.5)	4291 (54.5)	3755 (38.3)	6397 (45.2)	3406 (54.0)	2991 (38.1)	1649 (46.5)	885 (56.2)	764 (38.8)
West	2735 (15.5)	907 (11.5)	1828 (18.6)	2210 (15.6)	738 (11.7)	1472 (18.8)	525 (14.8)	169 (10.7)	356 (18.1)
Demographic characteristics									
High school graduate	15 016 (84.9)	6125 (77.7)	8891 (90.6)	12 009 (84.9)	4899 (77.7)	7110 (90.7)	3007 (84.8)	1226 (77.8)	1781 (90.5)
Household income >$60 000	4206 (23.8)	1657 (21.0)	2549 (26.0)	3356 (23.7)	1327 (21.1)	2029 (25.9)	850 (24.0)	330 (20.9)	520 (26.4)
Female	9880 (55.9)	4492 (57.0)	5388 (54.9)	7907 (55.9)	3595 (57.0)	4312 (55.0)	1973 (55.7)	897 (56.9)	1076 (54.7)
Male	7809 (44.1)	3387 (43.0)	4422 (45.1)	6238 (44.1)	2708 (43.0)	3530 (45.0)	1571 (44.3)	679 (43.1)	892 (45.3)
Party									
Democrat	6977 (39.4)	4228 (53.7)	2749 (28.0)	5594 (39.5)	3385 (53.7)	2209 (28.2)	1383 (39.0)	843 (53.5)	540 (27.4)
Republican	4376 (24.7)	699 (8.9)	3677 (37.5)	3494 (24.7)	553 (8.8)	2941 (37.5)	882 (24.9)	146 (9.3)	736 (37.4)
Independent	6336 (35.8)	2952 (37.5)	3384 (34.5)	5057 (35.8)	2365 (37.5)	2692 (34.3)	1279 (36.1)	587 (37.2)	692 (35.2)
Preventive practices^b^									
Mask in (always)	12 106 (68.4)	5316 (67.5)	6790 (69.2)	9648 (68.2)	4230 (67.1)	5418 (69.1)	2458 (69.4)	1086 (68.9)	1372 (69.7)
Mask out (always)	5517 (31.2)	3513 (44.6)	2004 (20.4)	4408 (31.2)	2807 (44.5)	1601 (20.4)	1109 (31.3)	706 (44.8)	403 (20.5)
Wash hands (always)	10 779 (60.9)	5084 (64.5)	5695 (58.1)	8688 (61.4)	4090 (64.9)	4598 (58.6)	2091 (59.0)	994 (63.1)	1097 (55.7)
Distance (always)	9461 (53.5)	4587 (58.2)	4874 (49.7)	7571 (53.5)	3681 (58.4)	3890 (49.6)	1890 (53.3)	906 (57.5)	984 (50.0)

^a^ This table presents summary statistics on baseline variables for our main sample of individuals who completed all baseline variables.

^b^ The preventive practices variables refer to the question: “What fraction of the time would you say that you engage in the following behaviors?” Mask in (always) is equal to 1 if the respondent answered “always” to “Wearing a mask when you go inside buildings that are not your home / take public transportation,” otherwise it is 0. Mask out (always) is equal to 1 if the respondent answered “always” to “Wearing a mask outside,” otherwise it is 0. Wash hands (always) is equal to 1 if the respondent answered “always” to “Washing your hands with soap and water right away when you come home after going out.” Distance (always) is equal to 1 if the respondent answered “always” to “Staying at least 6 feet away from people who are not part of your household.”

### Attrition

Due to the online nature of the survey, participants could exit at any point after watching the video messages without finishing the survey. To include as many participants as possible in the analysis, we included everyone who answered knowledge questions for the knowledge outcome. Overall, 18 762 participants included in the initial randomization were included at that stage (9445 Black; 9317 White). For other outcomes, except follow-up outcomes, we included 18 223 participants (9168 Black; 9055 White) who completed the survey. Attrition was similar in all groups (eTable 1B in Supplement 1).

The adherence to safety behavior was collected a few days later among a smaller follow-up sample that experienced more attrition. Overall, 12 591 individuals were included in the follow-up sample to track; only people who had given permission to Lucid to be recontacted were included in this sample. Of those, we successfully contacted 6217. Attrition at this stage was 51.8% in the treatment group and 51.0% in the control group. A systematic analysis of attrition at all 3 stages (eTable 1B in Supplement 1) revealed no systematic difference between the characteristic of attritors in the treatment and comparison groups. At the realized sample size, the power was 0.055 SDs for the knowledge sample (18 762 respondents), 0.056 SDs for all other outcomes in the first survey, and 0.095 SDs for the follow up sample (6217 respondents).

### Effects of Any Video Message, Intervention vs Control

Receiving any COVID-19 video improved knowledge of COVID-19 and adherence to preventive practices. Table 2 shows main outcomes overall and by racial group.

**Table 2.** Impact of Any Video Message Intervention vs Control: Incidence Rate^a^

Panel	Outcome	Control group		Intervention group		IRR (CI 95%)	*P* value	q value	Observations, No.
		Mean incidence rate (95% CI)	Observations, No.	Mean incidence rate (95% CI)	Observations, No.				
All participants	Knowledge gap score	0.241 (0.235 to 0.246)	3763	0.214 (0.211 to 0.217)	14 999	0.89 (0.87 to 0.91)	<.001	<.001	18 762
	Information seeking behavior	0.32 (0.31 to 0.33)	3654	0.338 (0.332 to 0.344)	14 569	1.05 (1.01 to 1.11)	.03	.04	18 223
	Safety gap score	0.47 (0.45 to 0.48)	1212	0.45 (0.44 to 0.46)	4823	0.96 (0.92 to 1.01)	.08	.08	6035
	Knowledge gap follow-up	0.25 (0.24 to 0.26)	1211	0.241 (0.238 to 0.244)	4819	0.956 (0.917 to 0.996)	.03	.04	6030
Black participants^b^	Knowledge gap score	0.316 (0.307 to 0.324)	1892	0.297 (0.292 to 0.301)	7553	0.94 (0.91 to 0.97)	<.001	<.001	9445
	Information seeking behavior	0.38 (0.36 to 0.40)	1840	0.40 (0.39 to 0.41)	7328	1.06 (1.00 to 1.12)	.06	.15	9168
	Safety gap score	0.40 (0.36 to 0.43)	416	0.38 (0.36 to 0.40)	1683	0.95 (0.87 to 1.04)	.26	.28	2099
	Knowledge gap follow-up	0.27 (0.25 to 0.28)	416	0.258 (0.253 to 0.263)	1681	0.97 (0.91 to 1.03)	.28	.28	2097
White participants^b^	Knowledge gap score	0.165 (0.159 to 0.170)	1871	0.131 (0.128 to 0.133)	7446	0.80 (0.76 to 0.83)	<.001	<.001	9317
	Information seeking behavior	0.26 (0.25 to 0.28)	1814	0.275 (0.267 to 0.282)	7241	1.05 (0.97 to 1.13)	.22	.22	9055
	Safety gap score	0.50 (0.48 to 0.53)	796	0.49 (0.48 to 0.50)	3140	0.96 (0.91 to 1.02)	.20	.22	3936
	Knowledge gap follow-up	0.24 (0.23 to 0.25)	795	0.231 (0.228 to 0.235)	3138	0.95 (0.89 to 1.00)	.05	.09	3933
**Panel**	**Outcome**	**Mean (95% CI), $**	**Observations, No.**	**Mean (95% CI), $**	**Observations, No.**	**Coefficient (95% CI)**	***P *value**	**q Value**	**Observations, No.**
All	WTP masks	14.07 (13.76 to 14.38)	3360	14.58 (14.42 to 14.74)	13 399	0.50 (0.15 to 0.85)	.005	.01	16 759
Black	WTP masks	15.70 (15.22 to 16.18)	1550	16.13 (15.87 to 16.38)	6175	0.42 (−0.14 to 0.97)	.14	.24	7725
White	WTP masks	12.68 (12.29 to 13.07)	1810	13.26 (13.06 to 13.46)	7224	0.57 (0.12 to 1.01)	.01	.01	9034

Abbreviations: IRR, incidence rate ratio; WTP, willingness to pay.

^a^ Incidence rate for knowledge gaps is the count of knowledge gaps divided by the maximum possible count (10). Incidence rate for interest in links is the count of links demanded divided by the maximum possible count (5). Incidence rate for safety gaps is the count of safety gaps divided by the maximum possible count (4). IRR (or coefficients) compare the any intervention with the control group. IRRs for safety gap score were estimated by fitting a negative binomial regression model with units weighted following Hainmueller entropy-based weighting^9^ to account for imbalances due to attrition for the follow-up outcomes. q values are reported accounting for the different outcomes and coefficients in each panel.

^b^ The *F* statistic for a test equality of the coefficients for the Black participants and White participants (obtained by estimating all outcomes in a joint system) was 0.0112 (*P* > .99).

#### Main Sample

**Figure 2.**
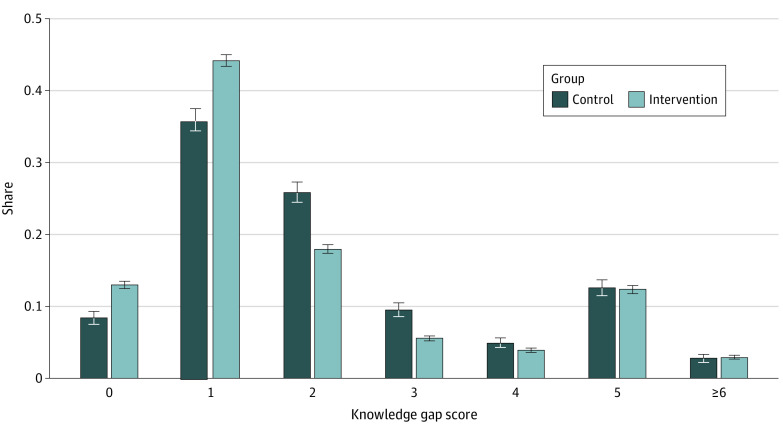
Distribution of the Knowledge Gap Score in the Control and Intervention Groups Whiskers indicate 95% CIs.

The knowledge gap incidence rate was 0.241 (95% CI, 0.235-0.246) in the control group and 0.214 (95% CI, 0.211-0.217) in intervention group. The intervention had a significant effect on reducing knowledge gaps relative to the control group (estimated incidence rate ratio [IRR], 0.89 [95% CI, 0.87-0.91]; *P* < .001; q < .001). In the control group, 315 participants (8.4%) had no gap in knowledge (Figure 2). The proportion increased to 13.0% (1915 participants) in the intervention group; 1352 participants (35.9%) in the control group had a 1-point knowledge gap compared with 6636 (44.2%) in the intervention group. The incidence rate of information seeking behavior was 0.320 in the control group and 0.338 in the intervention group (estimated IRR, 1.05 [95% CI, 1.01-1.11]; *P* = .03; q = .04). The willingness to pay for a mask increased from $14.07 in the control group to $14.58 in the intervention group (difference, $0.50 [95% CI, $0.15-$0.85]; *P* = .005; q = .013).

The intervention was impactful for both Black and White participants. It was more impactful for White participants vs Black participants on knowledge (IRR, 0.80 [95% CI, 0.76-0.83] vs 0.94 [95% CI 0.91-0.97]; *P* for difference < .001), but equally impactful for all the other measures (eTable 2C in Supplement 1). The *F* statistics for the hypothesis that the coefficients across all outcomes are jointly different for both races was 0.0112 (*P* > .99).

#### Follow-up Survey

At the follow-up survey (Table 2), which was realized on a smaller sample with larger attrition, the safety gap index incidence rate was 0.47 (95% CI, 0.45-0.48) in the control group and 0.45 (95% CI, 0.44-0.46) in the treatment group (IRR, 0.96 [95% CI 0.92-1.01]; *P* = .08, q = .08). Overall, 244 participants (20.1%) and 218 participants (18.0%) in the control group and 1040 participants (21.6%) and 837 participants (17.4%) in the intervention group reported respecting all and none, respectively, of 4 safety practices (eFigure 2 in Supplement 1).

#### Supplemental Outcomes

eTable 3 in Supplement 1 reports effects on secondary outcomes. One noteworthy result is that we found significant effects of the intervention on donations both to a COVID-19 charity (vs Alzheimer) and to a COVID-19 relief charity specific to Black US residents (vs COVID-19 economic relief for everyone).

### Effects of the Framing of the Videos

None of the efforts to make the messages more relevant to the Black community had a differential effect on knowledge or individual behavior (Table 3). An *F* statistic that all effects for Black physician, AMA antiracism statement, and disproportionate burden statement are jointly different from 0 was 0.324 (*P* > .99).

**Table 3.** Impact of Tailoring Messages: Incidence Rate Ratios^a^

Panel	Outcome	Intervention group						Control group				Intervention vs control		Observations, No.
		Black physician		AMA antiracism		Racial disparity		Black physician		AMA antiracism				
		IRR (95% CI)	*P *value	IRR (95% CI)	*P *value	IRR (95% CI)	*P *value	IRR (95% CI)	*P *value	IRR (95% CI)	*P* value	IRR (95% CI)	*P* value	
All participants	Knowledge gap score	1.01 (0.96 to 1.06)	.66	0.99 (0.94 to 1.04)	.71	1.01 (0.98 to 1.03)	.66	0.99 (0.95 to 1.04)	.78	0.99 (0.95 to 1.03)	.65	0.89 (0.85 to 0.93)	<.001	18 762
	Information seeking	1.04 (0.95 to 1.14)	.42	1.03 (0.93 to 1.13)	.60	1.02 (0.98 to 1.07)	.30	0.96 (0.89 to 1.05)	.38	0.97 (0.89 to 1.05)	.42	1.01 (0.93 to 1.10)	.83	18 223
	Safety gap score	1.04 (0.95 to 1.14)	.44	1.03 (0.93 to 1.12)	.74	1.01 (0.97 to 1.05)	.73	0.97 (0.89 to 1.05)	.44	0.95 (0.87 to 1.03)	.20	0.93 (0.86 to 1.01)	.09	6035
	Knowledge gap follow up	0.98 (0.90 to 1.07)	.65	0.98 (0.90 to 1.07)	.63	1.01 (0.98 to 1.05)	.47	1.01 (0.94 to 1.09)	.72	0.98 (0.91 to 1.06)	.66	0.97 (0.90 to 1.04)	.38	6030
Black participants	Knowledge gap score	1.01 (0.94 to 1.07)	.89	1.01 (0.95 to 1.07)	.84	1.001 (0.98 to 1.04)	.55	1.00 (0.94 to 1.05)	.89	0.98 (0.93 to 1.04)	.48	0.93 (0.88 to 0.98)	.009	9445
	Information seeking	1.04 (0.92 to 1.17)	.58	1.02 (0.91 to 1.15)	.76	1.02 (0.97 to 1.08)	.45	0.97 (0.84 to 1.08)	.60	0.98 (0.88 to 1.09)	.75	1.02 (0.92 to 1.14)	.71	9168
	Safety gap score	1.01 (0.85 to 1.20)	.93	1.03 (0.87 to 1.23)	.72	1.12 (1.04 to 1.21)	.005	0.98 (0.84 to 1.15)	.79	0.87 (0.74 to 1.02)	.08	0.88 (0.76 to 1.02)	.10	2099
	Knowledge gap follow up	0.97 (0.86 to 1.10)	.66	0.99 (0.87 to 1.11)	.81	1.05 (0.99 to 1.11)	.08	1.02 (0.91 to 1.13)	.78	0.97 (0.87 to 1.09)	.63	0.96 (0.87 to 1.07)	.49	2097
White participants	Knowledge gap score	1.03 (0.94 to 1.12)	.55	0.96 (0.88 to 1.04)	.32	1.00 (0.96 to 1.04)	.87	0.99 (0.92 to 1.06)	.74	1.01 (0.94 to 1.09)	.78	0.80 (0.75 to 0.87)	<.001	9317
	Information seeking	1.05 (0.90 to 1.22)	.56	1.03 (0.89 to 1.20)	.68	1.03 (0.96 to 1.10)	.46	0.95 (0.83 to 1.09)	.48	0.95 (0.83 to 1.08)	.43	1.00 (0.87 to 1.14)	.96	9055
	Safety gap score	1.06 (0.94 to 1.19)	.35	1.01 (0.90 to 1.13)	.89	0.94 (0.89 to 0.99)	.02	0.96 (0.87 to 1.07)	.44	1,00 (0.90 to 1.11)	.93	0.96 (0.87 to 1.07)	.47	3936
	Knowledge gap follow up	0.99 (0.88 to 1.11)	.81	0.98 (0.87 to 1.09)	.68	0.98 (0.93 to 1.04)	.50	1.01 (0.92 to 1.12)	.81	0.99 (0.90 to 1.10)	.87	0.97 (0.88 to 1.08)	.58	3933
**Panel**	**Outcome**	**Coefficient (95% CI)**	***P *value**	**Coefficient (95% CI)**	***P *value**	**Coefficient (95% CI)**	***P *value**	**Coefficient (95% CI)**	***P *value**	**Coefficient (95% CI)**	***P *value**	**Coefficient (95% CI)**	***P *value**	**Observations, No.**
All	WTP masks	−0.21 (−0.91 to 0.49)	.56	−0.29 (−0.99 to 0.41)	.42	0.07 (−0.24 to 0.39)	.65	0.17 (−0.45 to 0.80)	.59	0.32 (−0.30 to 0.95)	.31	0.71 (0.09 to 1.34)	.03	16 759
Black	WTP masks	0.04 (−1.08 to 1.15)	.95	−0.09 (−1.21 to 1.02)	.87	0.001 (−0.498 to 0.501)	>.99	−0.01 (−1.01 to 0.98)	.98	0.15 (−0.84 to 1.15)	.76	0.45 (−0.56 to 1.45)	.38	7725
White	WTP masks	−0.43 (−1.31 to 0.46)	.35	−0.46 (−1.35 to 0.42)	.30	0.13 (−0.26 to 0.53)	.51	0.33 (−0.46 to 1.12)	.41	0.47 (−0.32 to 1.26)	.24	0.94 (0.15 to 1.74)	.02	9034

Abbreviations: IRR, incidence rate ratio; WTP, willingness to pay.

^a^ The test statistics for the hypothesis that all the interaction coefficients are jointly 0 across equations was 0.324 (*P* > .99). Estimates in each row came from a single negative binomial regression (or ordinary least squares regression for WTP masks) following the second equation in the main text. IRRs for follow-up outcomes were calculated from estimates obtained by fitting a negative binomial regression model with units reweighted following Hainmueller entropy-based reweighting^9^ to account for imbalances due to attrition.

The only outcome that was affected by some of these variants was the desired donation to a Black-specific COVID-19 charity (eTable 4 in Supplement 1). eTable 5 in Supplement 1 shows that the combination of the AMA antiracism statement, a Black physician, and a video acknowledging racial disparities significantly increased how White and Black participants allocated donations to a charity focused on Black communities (ordinary least squares coefficient, $30.60 [95% CI, $10.93-$50.27]; *P* = .002).

### Heterogeneity by Sex, Education, Income, and Political Affiliation

Across all conditions, there were no statistically significant differences by sex or political affiliation (eTable 2A and eTable 2E in Supplement 1). The effect of intervention relative to control on knowledge gaps was more pronounced for participants with a high school education or more (eTable 2B in Supplement 1). However, there was no significant difference for the demand for links and willingness to pay for a mask. The intervention was more impactful among participants with lower incomes (ie, <$60 000).

## Discussion

Exposure to public health video messages about COVID-19 recorded by a diverse set of physicians decreased knowledge gaps on COVID-19 symptoms, preventive behaviors, and transmission among Black and White participants with modest incomes, relative to a control condition that saw placebo videos. The effect on knowledge was substantial and clear. This replicates the results of our prior study conducted in May 2020^3^ and extends it to White participants. New to this study, we also found a modest but statistically significant increase in the demand for more information, the willingness to pay for high quality masks, and self-reported behavior at follow-up.

Despite the heightened awareness of racial justice issues during the period of this intervention and increased polarization in the political discourse in the run-up to the presidential election, effects are remarkably similar across racial and political lines. These results suggest that physicians still have the ability to inform and persuade members of society from a broad range of backgrounds.

Our results also indicate that tailoring the message to specific communities did not affect its impact on behavior. Both White and Black physicians were able to effectively convey the importance of masking and social distancing to Black and White participants (unlike the previous study, in which concordance was important to change behavior^3^). The AMA antiracism statement did not affect participants’ attentiveness to the message delivered, for Black or White respondents. Acknowledgment of structural racism remains important, but it may not be sufficient to increase the level of trust from the Black community.

Importantly, the intervention made both Black and White participants more willing to focus resources both toward COVID-19 in general and toward the Black community in particular. Highlighting health conditions that disproportionately affect the Black community is one step toward increasing public consciousness of structural racism.

### Limitations

There are several limitations of the study. First, it was conducted online, and the participants may not be representative of the population with less than a college degree, given that they have access to the internet and are used to participating in online studies. Second, although information-seeking behavior and willingness to pay for masks were objectively measured, participants’ preventive health behaviors were not directly observed. Outcomes were self-reported. Third, outcomes might be subject to social desirability bias. Fourth, there may be bias due to attrition, particularly for the self-reported safety behavior, given that only a small fraction of the sample could be followed up a few days after the initial intervention. While the observable variables remain balanced, the unobservable may not be. Furthermore, while we found consistent effects on knowledge, information seeking, the willingness to pay for masks, and self-reported behavior, the final clinical significance of these findings is uncertain because effects on all were quantitatively small.

## Conclusions

These results suggest physician messaging campaigns may be effective and trust in Black and White physicians is equally high. There is no evidence of preexisting bias that would have led the intervention to have a negative effect. Because it is inexpensive, it could be a promising way to encourage behavior at scale. However, future studies implemented at a large scale are needed to confirm whether these kinds of interventions can change behavior in a way that will affect clinical outcomes. In ongoing work, we will study scale up messaging by doctors using social media.

## Notes

Supplement 1. **eAppendix 1.** Survey Design and Videos **eAppendix 2.** Supplementary Methods **eAppendix 3.** Robustness Checks and Subgroup Analysis **eFigure 1.** Full Study Flowchart **eFigure 2.** Distribution of the Safety Gap Score in the Control and Intervention Groups **eTable 1.** Balance and Attrition **eTable 2.** Outcomes by Subgroup **eTable 3.** Effects of Any Message Intervention: Effects on Additional Outcomes **eTable 4.** Effects of Tailoring Messages on Additional Outcomes **eTable 5.** Effects of All Black Treatments on Outcomes

Supplement 2. Pre-analysis Plan and Trial Protocol

Supplement 3. Nonauthor Contributors

Supplement 4. Data Sharing Statement
