# Supplementary material for: Effect of Physician-Delivered COVID-19 Public Health Messages and Messages Acknowledging Racial Inequity on Black and White Adults’ Knowledge, Beliefs, and Practices Related to COVID-19: A Randomized Clinical Trial
Source: JAMA Netw Open. 2021 Jul 14;4(7):e2117115. doi: 10.1001/jamanetworkopen.2021.17115 (PMC8280971; doi:10.1001/jamanetworkopen.2021.17115)
Supplement: Supplement 1. — eAppendix 1. Survey Design and Videos eAppendix 2. Supplementary Methods eAppendix 3. Robustness Checks and Subgroup Analysis eFigure 1. Full Study Flowchart eFigure 2. Distribution of the Safety Gap Score in the Control and Intervention Groups eTable 1. Balance and Attrition eTable 2. Outcomes by Subgroup eTable 3. Effects of Any Message Intervention: Effects on Additional Outcomes eTable 4. Effects of Tailoring Messages on Additional Outcomes eTable 5. Effects of All Black Treatments on Outcomes [file jamanetwopen-e2117115-s001.pdf]

Supplemental Online Content

Torres C, Ogbu-Nwobodo L, Alsan M, et al; COVID-19 Working Group. Effect of physician-delivered COVID-19 public health messages and messages acknowledging racial inequity on Black and White adults' knowledge, beliefs, and practices related to COVID-19. *JAMA Netw Open*. 2021;4(7):e2117115. doi:10.1001/jamanetworkopen.2021.17115

eAppendix 1. Survey Design and Videos

eAppendix 2. Supplementary Methods

eAppendix 3. Robustness Checks and Subgroup Analysis

eFigure 1. Full Study Flowchart

eFigure 2. Distribution of the Safety Gap Score in the Control and Intervention Groups

eTable 1. Balance and Attrition

eTable 2. Outcomes by Subgroup

eTable 3. Effects of Any Message Intervention: Effects on Additional Outcomes

eTable 4. Effects of Tailoring Messages on Additional Outcomes

eTable 5. Effects of All Black Treatments on Outcomes

This supplemental material has been provided by the authors to give readers additional information about their work.

© 2021 Torres C et al. *JAMA Network Open.*

eAppendix 1. Survey Design and Videos

Section A. Initial Survey Design

For a detailed flow chart of the survey design, see Supplement Figure 1. In this section, we present a copy of the survey instrument. Page formatting has not been preserved.

Survey Instrument

[Start of Block: Consent]

[irb_consent] We are a non-partisan group of academic researchers from Harvard University, the Massachusetts General Hospital (M.G.H.), the Massachusetts Institute of Technology (M.I.T.), Stanford University and Yale University. Our goal is to learn about people's attitudes on several issues related to the novel coronavirus (COVID-19). Please read the information below before consenting to begin the survey.

This survey is voluntary. You have the right to not answer any question, and to stop the survey at any time or for any reason (to exit the survey, simply close this window). We expect that the survey will take about 15 minutes. The survey platform, Lucid.io, may recontact you on our behalf for follow-up surveys. We will not receive your contact information. By accepting these terms you accept to be recontacted by Lucid on our behalf and to take part in the follow-up survey which will work under the same terms. Your name will never be recorded by researchers. Results may include summary data, but you will never be identified. The data will be stored on M.I.T. servers and will be kept confidential. The collected anonymous data may be made available to other researchers for replication purposes. You will be compensated for this interview conditional upon (i) completing the survey and (ii) passing our survey quality checks, which use sophisticated statistical control methods to detect incoherent and rushed responses. Responding without adequate effort may result in your responses being flagged for low quality and you may not receive your payment. Please note that it is very important for the success of our research that you answer honestly and read the questions very carefully before answering. If at any time you don't know an answer, please give your best guess without consulting any external sources. However, please be sure to spend enough time reading and understanding the questions. You are encouraged to print or take a screenshot of this page for your records. If you have any question about this study, you may contact us at coviddocstudy@gmail.com. If you have questions regarding your rights as a research subject, you may contact the Chairman of the Committee on the Use of Humans as Experimental Subjects, M.I.T., Room E25-143b, 77 Massachusetts Ave, Cambridge, MA 02139, phone 1-617-253-6787.

o Yes, I would like to take part in this study, and confirm that I LIVE IN THE U.S., and I am 18 or older (1)

o No, I would not like to participate (2)

[page break]

© 2021 Torres C et al. *JAMA Network Open.*

[welcome] Welcome to our study! First, we will ask you a few questions. Then, we will show you videos recorded by Medical Doctors. Finally we will ask some more questions. It is very important that you pay close attention to the information in this study. We hope you find it interesting!

[End of Block: Consent]

[Start of Block: COVID priors]

[cases_prior_bin] On average, in the past week, how many new cases of COVID do you think were diagnosed <u>each day</u> in the US?

- o Between 0 and 1,000 each day (2)
- o Between 1,001 and 10,000 each day (3)
- o Between 10,001 and 100,000 each day (4)
- o Between 100,001 and 200,000 each day (5)

[Display This Question: If On average, in the past week, how many new cases of COVID do you think were diagnosed each day in... = Between 0 and 1,000 each day]

[cases_prior_hun] Please indicate how many cases within you guess this range.

	0 100 200 300 400 500 600 700 800 900 1000
Number of cases	

[Display This Question: If On average, in the past week, how many new cases of COVID do you think were diagnosed each day in... = Between 1,001 and 10,000 each day]

[cases_prior_thou] Please indicate how many cases you guess within this range.

	1001 2001 3001 4001 5001 6000 7000 8000 9000 10000
Number of cases	

[Display This Question: If On average, in the past week, how many new cases of COVID do you think were diagnosed each day in... = Between 10,001 and 100,000 each day]

[cases_prior_ten_thou] Please indicate how many cases you guess within this range.

10001 30001 50001 70000 90000
20001 40001 60000 80000 100000

© 2021 Torres C et al. *JAMA Network Open.*

Number of cases	

[Display This Question: If On average, in the past week, how many new cases of COVID do you think were diagnosed each day in... = Between 100,001 and 200,000 each day]

[cases_prior_hun_thou] Please indicate how many cases you guess within this range.

100001 110001 120001 130001 140001 150001 160000 170000 180000 190000 200000

Number of cases	

[Page Break]

[bw_death_prior] Do you think that Black individuals are more or less likely to die from COVID-19 than White individuals of the same age? [order of options is randomized]

- ○ Black individuals are less likely to die than White individuals (1)
- ○ Black individuals are more likely to die than White individuals (2)
- ○ Black individuals are equally as likely to die as White individuals (3)

[Display This Question: If Do you think that Black individuals are more or less likely to die from COVID-19 than White indiv... = Black individuals are more likely to die than White individuals]

[b_death_mult_prior] How much more likely do you think Black individuals are to die from COVID-19 than White individuals of the same age?

1 2 3 4 5 6 7 8 9 10

Number of times more likely Black individuals are to die	

[Display This Question: If Do you think that Black individuals are more or less likely to die from COVID-19 than White indiv... = Black individuals are less likely to die than White individuals]

[w_death_mult_prior] How much more likely do you think White individuals are to die from COVID-19 than Black individuals of the same age?

1 2 3 4 5 6 7 8 9 10

Number of times more likely White individuals are to die	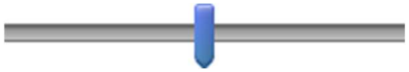

[End of Block: COVID priors]

[Start of Block: Baseline behaviors]

[attn_check_likely] It is important that we know if you are paying attention. Please select "Somewhat likely" if you are paying attention.
○ Very unlikely (1)
○ Somewhat unlikely (2)
○ Somewhat likely (3)
○ Very likely (4)

[safety_behaviors] What fraction of the time would you say that you engage in the following behaviors?

	Never (1)	Sometimes (2)	About half the time (3)	Most of the time (4)	Always (5)
Wearing a mask when you go inside buildings that are not your home / take public transportation (1)	○	○	○	○	○
Wearing a mask outside (2)	○	○	○	○	○
Washing your hands with soap and water right away when you come home after going out (3)	○	○	○	○	○
Staying at least 6 feet away from people who are not part of your household (4)	○	○	○	○	○

[Page Break]

© 2021 Torres C et al. *JAMA Network Open.*

[own_disposable_masks] Do you currently have more than 10 disposable masks at your home?
o Yes (1)
o No (2)

own_reusable_masks Do you currently own reusable masks (including home-made)?
o Yes, more than 3 in the home. (1)
o Yes, between 1 and 3 in the home. (2)
o No (3)

[Page Break]

[persons_met] Think about the most recent weekday. How many different people outside your household did you interact with at a distance of less than 6 feet?
o None (1)
o 1-3 (2)
o 4-10 (3)
o 11-20 (4)
o 21-30 (5)
o 31 or more (6)

[public_behavior] Think about the most recent weekday. Did you do any of the following activities:

	Yes (1)	No (2)
Eat a meal or had a drink inside an indoors cafe/restaurant/bar (1)	o	o
Watch a movie at a movie theater (2)	o	o
Go grocery shopping (3)	o	o
Go shopping for something else than groceries (4)	o	o
Take public transportation or a ride share or a taxi for work (5)	o	o
Take public transportation or a ride share or a taxi for a non-work related activity (6)	o	o

[Page Break]

© 2021 Torres C et al. *JAMA Network Open.*

[attn_check_color] It is important that we know you pay attention to this study. Please enter the word puce below when prompted for your favorite color.

What is your favorite color? [text entry field]

[End of Block: Baseline behaviors]

[Start of Block: AMA Video Introduction]

[ama_intro] Next, we would like you to watch a statement made by the American Medical Association.

[End of Block: AMA Video Introduction]

[Start of Block: Priming video]

[priming_instruct] Please watch the following video, and move on to the next page when you are done. Please do not spend more than 10 minutes on this page.

[priming video plays here]

[Page Break]

[priming_video_rating] Please rate the video you just watched on the following dimensions.

	Strongly agree (1)	Somewhat agree (2)	Neither agree nor disagree (3)	Somewhat disagree (4)	Strongly disagree (5)
The content is useful (1)	o	o	o	o	o
The content is trustworthy (2)	o	o	o	o	o

[End of Block: Priming video]

[Start of Block: Health / Wellness Video Introduction]

[Q171] We are now going to show you three short videos on health and wellness.

[End of Block: Health / Wellness Video Introduction]

[Start of Block: COVID Video Introduction]

© 2021 Torres C et al. *JAMA Network Open.*

[Q172] We are now going to show you three short videos on COVID-19.

[End of Block: COVID Video Introduction]

[Start of Block: Video 1 Doctor]

[video_1_instruct] Please watch the following video, and move on to the next page when you are done.

[Video 1 plays here]

[Page Break]

[video_1_rating] Please rate the video you just watched on the following dimensions.

	Strongly agree (1)	Somewhat agree (2)	Neither agree nor disagree (3)	Somewhat disagree (4)	Strongly disagree (5)
The content is useful (1)	○	○	○	○	○
The content is trustworthy (2)	○	○	○	○	○
I intend to follow the recommendations in the video (3)	○	○	○	○	○
I intend to share the information from this video with others (8)	○	○	○	○	○

[End of Block: Video 1 Doctor]

[Start of Block: Video 2 Doctor]

[video_2_instruct] Please watch the following video, and move on to the next page when you are done.

[Video 2 plays here]

[Page Break]

[video_2_rating] Please rate the video you just watched on the following dimensions.

© 2021 Torres C et al. *JAMA Network Open.*

	Strongly agree (1)	Somewhat agree (2)	Neither agree nor disagree (3)	Somewhat disagree (4)	Strongly disagree (5)
The content is useful (1)	○	○	○	○	○
The content is trustworthy (2)	○	○	○	○	○
I intend to follow the recommendations in the video (3)	○	○	○	○	○
I intend to share the information from this video with others (8)	○	○	○	○	○

[End of Block: Video 2 Doctor]

[Start of Block: Video 3 Doctor]

[video_3_instruct] Please watch the following video, and move on to the next page when you are done.

[Video 3 plays here]

[Page Break]

[video_3_rating] Please rate the video you just watched on the following dimensions.

	Strongly agree (1)	Somewhat agree (2)	Neither agree nor disagree (3)	Somewhat disagree (4)	Strongly disagree (5)
The content is useful (1)	○	○	○	○	○
The content is trustworthy (2)	○	○	○	○	○
I intend to follow the recommendations in the video (3)	○	○	○	○	○
I intend to share the information from this video with others (8)	○	○	○	○	○

[Page Break]

[thank_you_for_watch] Thank you for watching and rating these videos! We are now going to ask you some additional questions.

[End of Block: Video 3 Doctor]

[Start of Block: Outcomes - health]

[how_to_prevent] What are the **three most important things to do** to prevent the spread of COVID-19? Check up to three. [order of options is randomized]
- o Stay outdoors as much as possible and stay six feet away from other people, when around people outside of your household (1)
- o Wash your hands before going outside and when you come home (2)
- o Drink water regularly (3)
- o Regular outdoor exercise, such as playing basketball or soccer (4)
- o Wear a mask or facial covering (5)
- o Reduce screen time (6)
- o Regular exposure to sunlight (7)

[when_to_mask] What are the two situations where it is most important to wear a mask in public places?
- o Indoors, at all times (1)
- o Indoors when it is impossible to stay six feet away from people (2)
- o Outdoors when it is impossible to stay six feet away from people (3)
- o Outdoors, at all times (4)

[asymptomatic_infect] Can someone with COVID-19 infect others **without feeling sick or without showing any symptoms**?
- o Yes (1)
- o No (2)

[covid_symptoms] From the list below, select **4 symptoms** most commonly associated with COVID-19? [order of options is randomized]
- o Fever (1)
- o Hair loss (2)
- o Skin rash (3)
- o Cough (4)
- o Difficulty breathing (5)
- o Swollen legs (6)
- o Acid Reflux (7)
- o Back and/or knee pain (8)
- o New loss of taste or smell (9)

[End of Block: Outcomes - health]

[Start of Block: Outcomes – Posterior beliefs about COVID spread]

© 2021 Torres C et al. *JAMA Network Open.*

[cases_revision] On average, in the past week, how many new cases of COVID do you think were diagnosed each day in the US? Earlier, you answered [cases_prior answer] cases per day. Do you think this guess was too low, too high or just about right? We will then ask you to enter your revised guess no matter what you choose here.

o Too High (1)
o Too Low (2)
o Just About Right (3)

[cases_post_bin] Let's now enter your revised guess (which may be the same if you chose "Just About Right").
On average, in the past week, how many new cases of COVID do you think were diagnosed <u>each day</u> in the US?

o Between 0 and 1,000 each day (2)
o Between 1,001 and 10,000 each day (3)
o Between 10,001 and 100,000 each day (4)
o Between 100,001 and 200,000 each day (5)

[Display This Question : If Let's now enter your revised guess (which may be the same if you chose "Just About Right"). On av... = Between 0 and 1,000 each day]

[cases_post_hun] Please indicate how many cases within you guess this range.

	0 100 200 300 400 500 600 700 800 900 1000
Number of cases	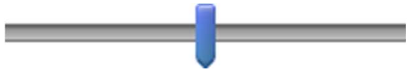

[Display This Question: If Let's now enter your revised guess (which may be the same if you chose "Just About Right"). On av... = Between 1,001 and 10,000 each day]

[cases_post_thou] Please indicate how many cases you guess within this range.

	1001 2001 3001 4001 5001 6000 7000 8000 9000 10000
Number of cases	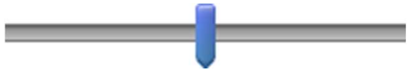

[Display This Question: If Let's now enter your revised guess (which may be the same if you chose "Just About Right"). On av... = Between 10,001 and 100,000 each day]

[cases_post_ten_thou] Please indicate how many cases you guess within this range.

10001 30001 50001 70000 90000
20001 40001 60000 80000 100000

© 2021 Torres C et al. *JAMA Network Open.*

Number of cases	

[Display This Question:If Let's now enter your revised guess (which may be the same if you chose "Just About Right"). On av... = Between 100,001 and 200,000 each day]

[cases_post_hun_thou] Please indicate how many cases you guess within this range.

100001 110001 120001 130001 140001 150001 160000 170000 180000 190000 200000

Number of cases	

[Page Break]

[Display This Question: If Do you think that Black individuals are more or less likely to die from COVID-19 than White indiv... = Black individuals are less likely to die than White individuals Or Do you think that Black individuals are more or less likely to die from COVID-19 than White indiv... = Black individuals are more likely to die than White individuals]
[bw_death_post] Previously, you answered that **[prior_more_likely]** individuals are **[prior_death_mult] times** more likely to die from COVID-19 compared to [prior_less_likely] individuals. Would you now want to revise it in any of the following ways: [randomize order of options]
o I now think this guess is just right (1)
o I now think this number is too high (2)
o I now think this number is too low (3)
o I now think [prior_less_likely] individuals are more likely to die compared to [prior_more_likely] individuals (4)

[Display This Question: If Do you think that Black individuals are more or less likely to die from COVID-19 than White indiv... = Black individuals are equally as likely to die as White individuals]

[bw_death_post_equal] Previously, you answered that Black individuals are equally likely to die from COVID-19 compared to White individuals. Would you now want to revise it in any of the following ways: [randomize order of options]
o I now think this guess is just right (1)
o I now think Black individuals are more likely to die compared to White individuals (2)
o I now think White individuals are more likely to die compared to Black individuals (3)

[Display This Question: If Do you think that Black individuals are more or less likely to die from COVID-19 than White indiv... = Black individuals are more likely to die than White individuals And Previously, you answered that [prior_more_likely] individuals are ... = I now think this number is too high
Or If Do you think that Black individuals are more or less likely to die from COVID-19 than White indiv... = Black individuals are more likely to die than White individuals And Previously, you answered that [prior_more_likely] individuals are ... = I now think this number is too low

© 2021 Torres C et al. *JAMA Network Open.*

Or If Do you think that Black individuals are more or less likely to die from COVID-19 than White indiv... = Black individuals are more likely to die than White individuals And Previously, you answered that [prior_more_likely] individuals are ... = I now think this guess is just right
Or If Do you think that Black individuals are more or less likely to die from COVID-19 than White indiv... = Black individuals are less likely to die than White individuals And Previously, you answered that [prior_more_likely] individuals are ... = I now think [prior_less_likely] individuals are more likely to die compared to [prior_more_likely] individuals
Or If Do you think that Black individuals are more or less likely to die from COVID-19 than White indiv... = Black individuals are equally as likely to die as White individuals And Previously, you answered that Black individuals are equally likely to die from COVID-19 compared... = I now think Black individuals are more likely to die compared to White individuals]

[b_death_mult_post] How much more likely do you think Black individuals are to die from COVID-19 than White individuals of the same age?

	1 2 3 4 5 6 7 8 9 10
Number of times more likely Black individuals are to die	

[Display This Question: If Do you think that Black individuals are more or less likely to die from COVID-19 than White indiv... = Black individuals are less likely to die than White individuals And Previously, you answered that [prior_more_likely] individuals are ... = I now think this number is too high
Or If Do you think that Black individuals are more or less likely to die from COVID-19 than White indiv... = Black individuals are less likely to die than White individuals And Previously, you answered that [prior_more_likely] individuals are ... = I now think this number is too low
Or If Do you think that Black individuals are more or less likely to die from COVID-19 than White indiv... = Black individuals are less likely to die than White individuals And Previously, you answered that [prior_more_likely] individuals are ... = I now think this guess is just right
Or If Do you think that Black individuals are more or less likely to die from COVID-19 than White indiv... = Black individuals are more likely to die than White individuals And Previously, you answered that [prior_more_likely] individuals are ... = I now think [prior_less_likely] individuals are more likely to die compared to [prior_more_likely] individuals
Or If Do you think that Black individuals are more or less likely to die from COVID-19 than White indiv... = Black individuals are equally as likely to die as White individuals And Previously, you answered that Black individuals are equally likely to die from COVID-19 compared... = I now think White individuals are more likely to die compared to Black individuals]

[w_death_mult_post] How much more likely do you think White individuals are to die from COVID-19 than Black individuals of the same age?

	1 2 3 4 5 6 7 8 9 10
Number of times more likely White individuals are to die	

[End of Block: Outcomes – Posterior beliefs about COVID spread]

[Start of Block: Outcomes – political opinions]

© 2021 Torres C et al. *JAMA Network Open.*

[fed_balance] How well do you think the **federal government** managed to balance opening the economy and limiting the health impacts of Covid-19?
- o They were too fast in encouraging re-opening (1)
- o They managed the balance just right (2)
- o They were too slow in encouraging re-opening (3)

[state_balance] How well do you think **your state government** managed to balance opening the economy and limiting the health impacts of Covid-19?
- o My state opened too quickly (1)
- o My state decided appropriately (2)
- o My state opened too slowly (3)

[End of Block: Outcomes – political opinions]

[Start of Block: Outcomes – Donation Slider: COVID vs. non- COVID] [Randomize order in which two options are presented in text and on slider]

[char_cov_sli] The research team will **donate $1,000** to two charitable organizations. **Give a Mask** provides **masks** to doctors, nurses, first responders, and essential workers to fight COVID-19. **The Alzheimer's Association** funds research to fight and cure Alzheimer's disease, and funds care and support groups for those already affected. How do you recommend we split our $1,000 donation between these groups? We will **donate based on a randomly chosen participant's decision — it could be you.**

Give a Mask Alzheimer's Association

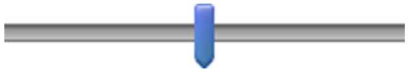

[End of Block: Outcomes – Donation Slider: COVID vs. non- COVID]

[Start of Block: Outcomes – Donation Slider: Black vs. General] [Randomize order in which two options are presented in text and on slider]

[char_blk_sli] The research team will **donate $1,000** to two groups fighting the economic impact of COVID-19. The **BET COVID-19 Relief Fund** supports community organizations, with a focus on Black families who need food assistance and emergency support. **GiveDirectly Project 100+** directly supports low-income families who need food assistance. How do you recommend we split our $1,000 donation between these groups? We donate based on **a randomly chosen participant's decision — it could be you**.

BET Fund Project 100+

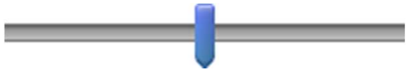

[End of Block: Outcomes – Donation Slider: Black vs. General]

[Start of Block: Outcomes – WTP question]

© 2021 Torres C et al. *JAMA Network Open.*

[wtp_masks] In this question we ask you how much a pair of reusable cloth masks is worth to you. Some individuals will be chosen to get a coupon worth 2 reusable cloth masks, made from a breathable, stretchable fabric and available in many popular designs. Some others will get an Amazon gift card. We explain how the prizes will be awarded below. Do not worry about the details of this reward system. It is in your best interest to simply **report how much a pair of reusable cloth masks is worth to you — how much you would be willing to pay for them in a store.**

	$0 $20 $40
How much US$ are 2 reusable masks worth to you?	

[Q167 WTP Instructions displayed underneath slider]
- You will first tell us how much you would pay for two reusable masks.
- A lottery will decide if you receive any prize or coupon. The probability to be chosen is 1/1000.
- If you are selected, the computer will choose a random prize value between $0 and $40 (uniformly).
- If the prize value is equal to or above your stated value for the masks, you will get an Amazon gift card equal to this prize amount.
- If the prize value is less than your stated value, you will get a coupon that can be redeemed for 2 reusable masks at an online store.

[End of Block: Outcomes – WTP question]

[Start of Block: Outcomes – Healthcare seeking behavior]

[link_choices] **The study is almost done, please make sure to finish.** Here are some additional resources you may find useful. Please let us know if you are interested in them, and we will provide a link on the next page. [order of options is randomized]

	I would like this link (1)	I am not interested in this link (4)
Videos about **working out in your home** (3)	○	○
**Your state's** public health information **hotline** (6)	○	○
Information on **testing locations** (7)	○	○
Masachussetts General Hostpital's (MGH) web resources about COVID-19 (8)	○	○
A **symptom tracker app** from Massachusetts General Hospital (MGH) (10)	○	○

[Page Break]

[Display This Question: If The study is almost done, please make sure to finish. Here are some additional resources you may... [I would like this link] (Count) > 0]

[link_display_text] Here are the links you expressed interest in. Please click on any you are interested in to open other tabs and/or copy any information for your records. Please return to the survey to complete it. This page will automatically skip to the next after 5 minutes.

[Display links selected by participant]

A symptom tracker app from Massachusetts General Hospital (MGH) [display if selected in link_choices]

A video about Working Out in a Small Space [display if selected in link_choices]]

Web Resources about COVID-19 from Massachussetts General Hospital (MGH) [display if selected in link_choices]]

Your State Hotline: [display state hotline here] [display if selected in link_choices]]

Information about Testing Locations [display if selected in link_choices]]

[End of Block: Outcomes – Healthcare seeking behavior in link_choices]]

[Start of Block: Draft outcome – Gift Card for Masks]

[congrats_maskforit] Congratulations! Your earned the mask gift card [display code]. You can redeem your gift card at https://maskforit.com/ The coupon is worth $25 on this website, which you can use towards 2 reusable masks + shipping fees. You can also use the coupon in any other way that you want. Please copy this information now and keep it safe. You will only see it once.

[End of Block: Draft outcome – Gift Card for Masks]

[Start of Block: Draft outcome – Gift Card for Amazon]

[congrats_amazon] Congratulations! You have been selected to receive an Amazon gift card. Your gift card code is [display code]. You can use this gift card on Amazon.com Please copy this information now and keep it safe. You will only see it once.

[End of Block: Draft outcome – Gift Card for Amazon]

[Start of Block: Debrief]

[researcher_expect] This is the last page of the survey. What do you think this study was about? What do you think the researchers expected to find? [text entry field]

© 2021 Torres C et al. *JAMA Network Open.*

[confusing_post_pilot] Did you find any question confusing? [text entry field]

[comment] You can leave any other comments in the box below. Please do not enter identifying information about yourself. We hope you remain in good health and spirits. [text entry field]

[End of Block: Debrief]
[End of Survey]

© 2021 Torres C et al. *JAMA Network Open.*

Section B: Scripts

Respondents watched a total of four videos. The scripts for each video are reproduced below.

Video 0

Introductory AMA Racism Statement (Intervention):
The American Medical Association recognizes that racism in its systemic, structural, institutional, and interpersonal forms is an urgent threat to public health, the advancement of health equity, and a barrier to excellence in the delivery of medical care. The American Medical Association opposes all forms of racism. The American Medical Association denounces police brutality and all forms of racially-motivated violence. The American Medical Association will actively work to dismantle racist and discriminatory policies and practices across all of health care.

Introductory Placebo Statement (Control):
The American Medical Association believes in transparency in prescription drug pricing, and we are pleased the House Ways & Means Committee moved the issue forward. Patients and their physicians want to be armed with more information, yet the current situation is opaque if not impenetrable. The committee is rightfully determined to expose factors that lead to high drug prices, and we look forward to continuing our efforts in that regard.

Video 1

Video 1 (Intervention):
Hello, I'm Dr [LAST NAME HERE] from [INSTITUTIONAL AFFILIATION HERE], and I'd like to tell you a little about Coronavirus or COVID-19. COVID-19 is a new virus that can infect the respiratory tract and lungs. Although many people who get sick from COVID will get better, some people who get it become very ill and some even die. Although there's no cure, there are ways medical professionals have found to protect you and your community from COVID. I hope that this message can give you information that will help you protect you or someone you love from COVID infection. First, I would like to tell you about the symptoms of COVID-19. The most common symptoms of COVID-19 are cough, fever, and trouble breathing. Another odd symptom some people have is loss of taste or smell. A large number of people who have COVID-19 actually don't show any symptoms at all. Unfortunately, people can still spread the disease to others even with no symptoms. The next video will provide you with more information on how you can protect yourself and others.

Video 1 (Control):
Most adults need to sleep between 6 and 8 hours a night. Now, there are some people who get five hours a night and they are fine, so there is some variation across people. But for most adults, we need 6 to 8 hours in order to function well the next day. If you feel sleep deprived you might not be able to function as well as you would normally like. It's important to have something called sleep hygiene which is a routine you follow at bedtime and can help you fall asleep. Things that can disrupt sleep hygiene include caffeine or alcohol too close to bedtime. Eating late at night can also cause indigestion. So keep a routine and trying to get 6-8 hours is important.

Video 2

© 2021 Torres C et al. *JAMA Network Open.*

Video 2 (Intervention):
Hello, I'm Dr [LAST NAME HERE] from [INSTITUTIONAL AFFILIATION HERE], you may be looking for ways to resume some activities as safely as possible. However, COVID-19 remains contagious and shows no signs of disappearing. In fact, during the week of July 6 there were 58,000 new COVID cases per day diagnosed in the United States. *[ONLY FOR UNEQUAL BURDEN SUB-INTERVENTION] Black Americans and other minority groups are three times as likely to get and, when you account for age, four times as likely to die from COVID as white Americans.* Without a safe and effective vaccine or therapy, our only option is to continue taking precautionary measures to protect ourselves, our communities, and the most vulnerable among us. While there is no way to ensure zero risk of infection from COVID-19, observing these three practices will help to protect you and others. First, continue to practice social distancing whenever possible: Try to stay outdoors, and to the maximum extent possible, please stay 6 feet apart. If you must be indoors, use visual reminders-like signs, chair arrangements, markings on the floor, or arrows-to help remind you to keep your distance from others, and maintain physical barriers whenever possible. Second, continue to wash your hands often for at least 20 seconds with soap and water, especially before going out, and every time you return home. Third, wear a mask when in public at all times, especially when indoors or when it is difficult to stay 6 feet away. The next video will tell you a bit more about masks.

Video 2 (Control):
Sugar is found in many different food items. Natural sugars are those that can be found in fruits, vegetables and dairy products like milk. Sugars like these that are natural are not really problematic because they are coming alongside lots of other vitamins and minerals. There are other sugars, though, that are processed and added to a food item. These are called additive sugars. A good rule of thumb is to eat foods with less than than 5g of sugar per serving. Avoid buying products where one of the first five products is a sugar. And it can be better to buy an unsweetened product like an unsweetened cereal or oatmeal and then add a teaspoon of sugar to it if you need the sweetness than to buy a heavily sweetened product, like a sugar cereal which can have several teaspoons of sugar per serving.

Video 3

Video 3 (Intervention):
Hello, I am doctor [LAST NAME HERE] from [INSTITUTIONAL AFFILIATION HERE], and I will tell you a bit more about masks. Wearing a mask is a key way to prevent the spread of COVID-19. You are not just protecting yourself but also your grandma and your community, just in case you have COVID-19 but don't know it. Even if wearing a mask may sometimes put you in a difficult situation, it is important to protect you and the community from COVID 19 disease. As medical professionals, I am committed to delivering the best care I can to every patient. My goal is to make sure that you and everyone you love survives this COVID-19 pandemic. Thank you for listening to these messages.

Video 3 (Control)
New fitness guidelines can be summed up as follows: just move and anything counts. Sneaking in a few minutes of physical activity throughout the day adds up in the long run. The guidelines are trying to make it easier for individuals to be fit and drop the rule that activity must be in 10 minute blocks of time. In a nutshell, activity has benefits even if it's for a short amount of time. Taking the stairs instead of the elevator, parking your car far away from the entrance to a store or walking your dog around the block can all help you be fit. The guidelines still call for at least 150 minutes a week of moderately intense aerobic exercise and two weekly sessions of muscle training activity, like lifting weights or yoga.

© 2021 Torres C et al. *JAMA Network Open.*

eAppendix 2. Supplementary Methods

Section C. Outcomes

This section reviews primary outcomes in more detail and describes secondary outcomes. The primary outcomes listed in ClinicalTrials.gov ID: NCT04502056 include "Knowledge Beliefs and Practices related to COVID-19" – we define knowledge gap outcomes in detail below which captures the knowledge and beliefs component. The seeking out of additional information and resources to protect oneself and one's family/community is the main behavioral outcome. Other primary outcomes are a safety gap outcome (for those who completed the follow up survey), the willingness to pay for a pair of reusable cloth masks and a charitable donation to a Black-targeted vs non targeted charity. The secondary outcomes in ClinicalTrials.gov ID: NCT04502056 and in the pre-analysis plan posted on https://www.socialscienceregistry.org/ included judgement of the federal and state policy responses, a charitable donation to COVID vs non COVID charity and a knowledge gap outcome of the follow up survey. They are shown in Supplementary Table 8a and 8b.

Knowledge Gaps (primary outcome): We measure knowledge through 3 questions described below.

Preventive practice: First, participants were asked to select three ways to prevent COVID-19 spread among a list that included staying six feet away from other people when outside, washing their hands when returning home and wearing a mask/facial covering when outside (See question "how_to_prevent" in the survey instrument). Each of these three practices that is not selected increases the count of **knowledge gaps in preventive practice** $\boldsymbol{g_p}$ by 1, from $g_p = 0$ up to $g_p = 3$.
Asymptomatic transmission: Second, participants were asked whether transmission by asymptomatic individuals is possible; those responding "no" were coded as having a knowledge gap for asymptomatic transmission ($g_a = 1$) while those responding "yes" were coded as having no knowledge gap for asymptomatic transmission ($g_a = 0$).
When to wear a mask: Third, participants were asked to identify the two situations where it is most important to wear a mask in public places. Each selected practice different from "Indoors, at all times" or "Outdoors when it is impossible to stay six feet away from people" increases the count of knowledge gaps in mask practice g_m by one, from $g_m = 0 \; to \; g_m = 2$.
Symptoms: Fourth, participants were asked about selecting exactly 4 common COVID-19 symptoms from a list. Each selected symptom that was not among cough, fever, difficulty breathing or a new loss of taste or smell increases the knowledge gap for symptoms $\boldsymbol{g_s}$ by 1, from $g_s = 0$ up to $g_s = 4$.
Knowledge Gaps Count: The knowledge gap count was defined as the sum of the three knowledge gaps ($G = g_p + g_a + \boldsymbol{g_m} + g_s$), taking any integer value between 0 and 10. This is our primary outcome.

Information Seeking Behavior (primary outcome): Participants were asked to indicate interest in any number of links to COVID-related resources among a list of 5. There were told that they would subsequently obtain the selected links at the end of the study. We define the behavior index as the count of links selected. This could take any integer value between 0 and 5. The 5 links are:

1. A video about Working Out in a Small Space

2. **Your state's** public health information **hotline** *[link personalized based on the participant's state]*

3. Information about Testing Locations

© 2021 Torres C et al. *JAMA Network Open.*

4. Web Resources about COVID-19 from Massachussetts General Hospital (MGH)

5. A symptom tracker app from Massachusetts General Hospital (MGH)

Safety gap score:

This outcome was measured a few days after the initial intervention, for a subsample that was eligible for follow up and could be tracked. Participants were asked about how often they engaged in four behaviors of interest (1. If they wore a mask indoors, 2. If they they wore a mask outdoors, 3. If they washed their hands, and 4. If they followed social distancing guidelines). A safety gap index was then calculated which has a value of 0 if they reported that they practiced the four behaviors of interest "always" up to 4 if they they report practicing none of them always.

Knowledge Gaps follow up: It is the same outcome as the primary knowledge gap outcome, except that it is in the follow up survey.

Willingness To Pay for Masks (primary outcome): Participants were asked how much a pair of reusable cloth masks is worth to them, i.e how much they would be willing to pay for them in a store. We define de WTP Mask index as the number of dollars selected by the respondent (between 0 and 40).

Secondary and Additional outcomes:

Donation to GiveDirectly Project 100+: Participants were asked to choose how $1000 USD from the research team should be allocated between an organization that directly supports low-income families who need food assistance versus a fund that supports community organizations, with a focus on Black families who need food assistance and emergency support. Outcome is the amount allocated to the first one, an integer between 0 and 1000.

Donation to BET COVID-19 Relief Fund (primary outcome): Participants were asked to choose how $1000 USD from the research team should be allocated between an organization that directly supports low-income families who need food assistance versus a fund that supports community organizations, with a focus on Black families who need food assistance and emergency support. Outcome is the amount allocated to the second one, an integer between 0 and 1000.

Trust in Federal response: Participants were asked how well they think the **federal government** managed to balance opening the economy and limiting the health impacts of Covid-19. This outcome takes value 1 if the respondent answered "They managed the balance just right"
Trust in Local response: Participants were asked how well they think the **state government** managed to balance opening the economy and limiting the health impacts of Covid-19. This outcome takes value 1 if the respondent answered "They managed the balance just right"

© 2021 Torres C et al. *JAMA Network Open.*

Section D. Balance and Attrition

We test for balance of baseline covariate distributions across intervention arms, first for the sample of individuals who were randomized in intervention or assigned to control and completed the baseline variables (Panel A of Supplement Table 1a), then for the subsample of individuals who stayed in the study at least up to the knowledge outcome (Panel B), then for the subsample of individuals who completed the link questions (Panel C), and finally for the subsample of people who completed the knowledge questions in the follow up survey (Panel D).

We use the function bal.tab() from the R package COBALT. We test for balance on marginal distribution of individual covariates, as well as for distribution of products of two covariates. We conduct these balance tests for all the intervention variations considered in our analysis: the group which receives intervention against the control group, the group which receives race concordant physician video against the group which does not, the group which receives an AMA statement acknowledging systemic racism against the group which receives an AMA placebo statement and the group which receives a message acknowledging increased incidence and mortality for Blacks against the group which received a standard message. For each comparison, we count the number of distributions that fail the balance test either by having a standardized mean difference greater than 0.1 or a Kolmogorov-Smirnov test p-value greater than 0.05, as recommended. Supplementary Table 1a report these counts for the full randomized sample, the sample who completed knowledge questions, the sample who completed links questions and the sample who completed knowledge questions in the follow up survey. We find no evidence of imbalance in all of these samples.

To account for attrition in the follow up survey which can potentially confound our estimates, we conduct Hainmueller's entropy balancing in models we present in the main text, following (1). This data preprocessing method is designed to achieve covariate balance in observational studies with binary interventions. It calibrates individual weights to ensure that reweighted intervention and control groups satisfy a large set of balance conditions on first and second moments of the covariate distributions. The list of baseline covariates that are used to implement this reweighting procedure is the following:

- Age
- Gender
- Party (democrat, republican)
- Stratum (which includes gender, an indicator for age below 44 years old, race, and self-reported Republican identification)
- Household income above 60k
- HS graduate
- The 4 safety practices (mask in, mask out, wash hands, distance)
- Prior belief "Blacks are more likely to die from COVID 19"

Subsequently, these weights are used in the models that we present in the main text. For completeness, we also present the results from unweighted regressions in Supplement Tables 5 and 6.

© 2021 Torres C et al. *JAMA Network Open.*

Section E. Regression Models for Additional Analyses

Section E.1. Controlling for more baseline covariates using Double Post LASSO

Here we describe how the Double Post Lasso method works for selecting additional control variables. We only use this method in additional analyses presented in Supplement Tables 3 and 4.

We do not include this approach in the primary analysis for simplicity and because it leads to very little precision gains in this application. We pre-specified to include baseline covariates chosen by a double-robust machine-learning algorithm (2). This was used because it is a procedure delivering consistent estimates of intervention effects while improving efficiency by selecting covariates that are relevant to avoid omitted variable bias but exclude those that likely do not have such a threat. Technical details are in (2). The procedure selects, in our case using LASSO, covariates that are correlated either with the outcome or with the intervention assignment. Notice both conditions can lead to omitted variable bias. So the LASSO procedure selects only relevant covariates that could have generated omitted variable bias (2).

Let Y denote the outcome variable, T the intervention variable and X a vector of covariates. Here are the three steps of the Double Post Lasso selection method.

- First, regress the intervention variable T on the covariates using a Lasso regression. Let S_T be the set of covariates which have a coefficient different from 0 in this regression.
- Then, regress the outcome variable Y on the covariates using a Lasso regression. Let S_Y be the set of covariates which have a coefficient different from 0 in this regression.
- Finally, fit the negative binomial regression models as described in the primary analysis where covariates in $S_Y \cup S_T$ are included as regressors. For example, the intervention regression model now writes:

$$\log(\mu_i) = \beta_0 + \beta_1 intervention_i + \beta_2^t stratum_i + \beta_3^t X_i$$

Where X_i is the vector of covariates from the set $S_Y \cup S_T$.

Lasso regressions were implemented using the functions *rlasso* from the R package *hdm* v0.3.1.

Section E.2. Regression models without weights

As detailed in Section D, to account for attrition which can potentially confound our estimates, we conduct Hainmueller's entropy balancing in models that we present in the main text, following (1). For completeness, we also perform unweighted regressions, and we present the results in Supplement Tables 5 and 6.

Section E.3. Regression models used for additional specifications and the analysis of secondary outcomes

© 2021 Torres C et al. *JAMA Network Open.*

Here we describe the regression models used for the analysis of secondary and additional outcomes presented in Section G and Supplement Tables 7 and 8.

In all of our regressions, we adapted the model to the distribution of the outcome. For count variables (our primary outcomes), we fit a negative binomial model using *glm.nb* from the *MASS* package, as presented in the main text. For the binary outcome (interest in the DIY mask video), we used logistic regression with the *glm* function from the *stats* package. For every other variables (Donation, Perceived norms around masks and video ratings), we used OLS regression with the *lm* function from the *stats* package. We present the regression equations for logistic regression and OLS below. Negative binomial models are presented in the main text.

In every regression, we control for strata. Below we show the regression equations.

In all regression equations, $stratum$ refers to a vector of length 12 that indicates the participant's stratum with a 1 on its corresponding coordinate and zeros on other coordinates.

- **Intervention Analysis:**

Sample: all intervention groups and control group. Participants who completed survey at least up to knowledge questions for our primary knowledge outcome. Participants who completed the entire survey for other outcomes.

The coefficient of interest for Intervention is β_1.

Binary outcomes - logistic regression equation:

$$\ln\left(\frac{p_i}{1-p_i}\right) = \beta_0 + \beta_1 intervention_i + \beta_2^T stratum_i$$

where Y_i is the outcome variable, $p_i = P(Y_i = 1)$.

Other outcomes - OLS regression equation:

$$Y_i = \beta_0 + \beta_1 intervention_i + \beta_2^T stratum_i$$

- **Tailoring Intervention Analysis:**

Sample: Participants who were assigned to an intervention group (i.e. not assigned to the control group). Participants who completed the survey at least up to knowledge questions for our primary knowledge outcome. Participants who completed the entire survey for other outcomes.

The coefficients of interest for Black Physician are β_1 and β_4.

© 2021 Torres C et al. *JAMA Network Open.*

Binary outcomes - logistic regression equation:

$$\ln\left(\frac{p_i}{1-p_i}\right) = \beta_0 + \beta_1 Black\ Physician_i + \beta_2 AMA\_Anti\_racism_i + \beta_3 intervention_i + \beta_4 Black\ Physician_i * intervention_i + \beta_5 AMA\_Anti\_racism_i * intervention_i + \beta_6 Mortality\_difference * intervention_i + \beta_7^T stratum_i$$

Other outcomes - OLS regression equation:

$$Y_i = \beta_0 + \beta_1 Black\ Physician_i + \beta_2 AMA\_Anti\_racism_i + \beta_3 intervention_i + \beta_4 Black\ Physician_i * intervention_i + \beta_5 AMA\_Anti\_racism_i * intervention_i + \beta_6 Mortality\_difference * intervention_i + \beta_7^T stratum_i$$

- **All Black treatments Analysis:**

Sample: Participants who were assigned to an intervention group (i.e. not assigned to the control group). Participants who completed the survey at least up to knowledge questions for our primary knowledge outcome. Participants who completed the entire survey for other outcomes.

In Supplement Table 12, we estimate the following equations.

Negative binomial regression equation:

$$\log(\mu_i) = \beta_0 + \beta_1 intervention_i * All\ black\ treatments_i + \beta_2 intervention_i + \beta_3 AMA\_Anti\_racism_i * Black\ Physician_i + \beta_4^T stratum_i$$

OLS regression equation

$$Y_i = \beta_0 + \beta_1 intervention_i * All\ black\ treatments_i + \beta_2 intervention_i + \beta_3 AMA\_Anti\_racism_i * Black\ Physician_i + \beta_4^T stratum_i$$

© 2021 Torres C et al. *JAMA Network Open.*

eAppendix 3. Robustness Checks and Subgroup Analysis

Figure 3.1. Equation 2 Residuals*

Knowledge gap score:

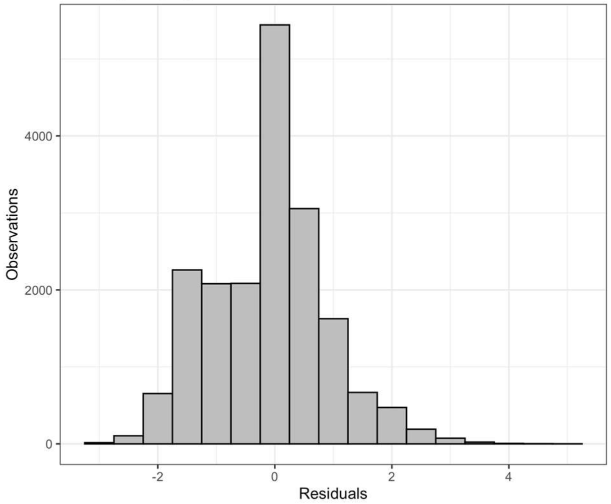

Information seeking behavior:

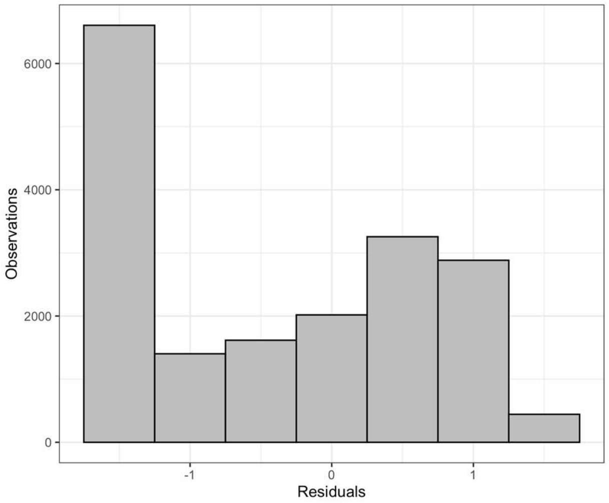

Safety gap score:

Knowledge gap follow up:

© 2021 Torres C et al. *JAMA Network Open.*

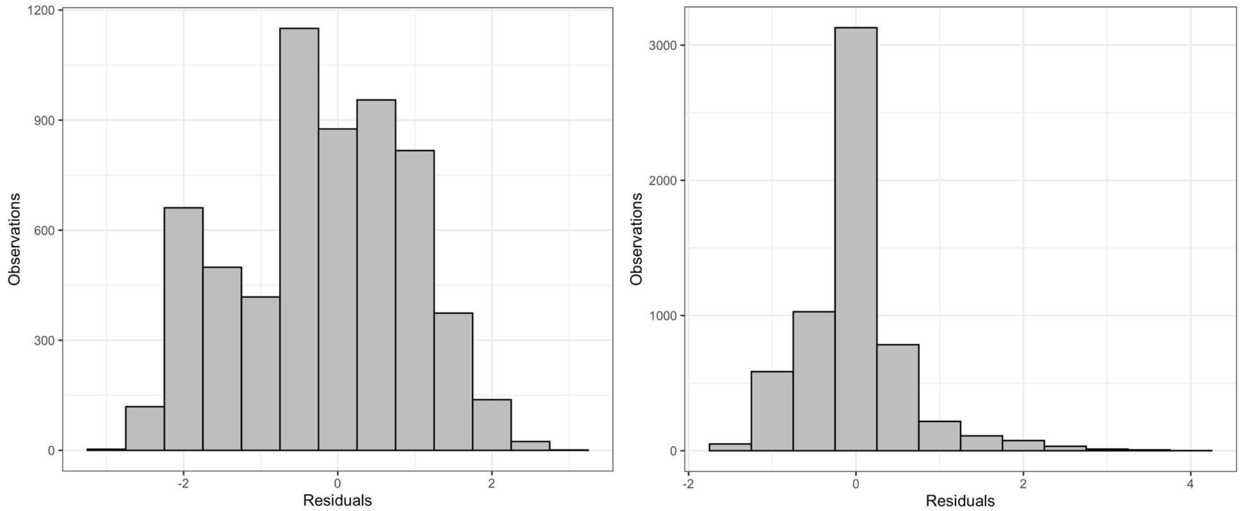

* This figure presents the histograms of the equation 2 residuals for the primary outcomes. The specifications of the regressions are the same as in Table 3 (with Panel = All participants). Incidence Rate Ratios (IRRs) for follow-up outcomes are calculated from estimates obtained by fitting a negative binomial regression model with units reweighted following Hainmueller's entropy-based reweighting to account for imbalances due to attrition (14).

Supplement Table 3.1. Effect of Any Message Intervention on Primary Outcomes, with Double Post Lasso Control Variables*

		IRR (CI 95%)	p-value	Observations
Panel A	Outcome			
All	Knowledge gap score	0.893 (0.871,0.915)	<0.001	18762
	Information seeking behavior	1.054 (1.007,1.103)	0.02	18223
	Safety gap score	0.974 (0.931,1.020)	0.27	6035
	Knowledge gap follow-up	0.959 (0.920,1.000)	0.05	6030
Black	Knowledge gap score	0.944 (0.916,0.973)	<0.001	9445
	Information seeking behavior	1.056 (0.997,1.118)	0.06	9168
	Safety gap score	0.962 (0.893,1.036)	0.31	2099
	Knowledge gap follow-up	0.977 (0.919,1.039)	0.47	2097

© 2021 Torres C et al. *JAMA Network Open.*

White	Knowledge gap score	0.800 (0.767,0.835)	<0.001	9317
	Information seeking behavior	1.058 (0.985,1.138)	0.12	9055
	Safety gap score	0.987 (0.931,1.047)	0.67	3936
	Knowledge gap follow-up	0.943 (0.891,0.999)	0.04	3933
Panel B	Outcome	**coefficient (CI 95%)**	**p-value**	**Observations**
All	**WTP Masks**	0.522 (0.195,0.849)	0.002	16759
Black	**WTP Masks**	0.480 (-0.018,0.977)	0.06	7725
White	**WTP Masks**	0.585 (0.158,1.011)	0.007	9034

*This table presents IRR (or OLS coefficient for WTP Masks) of the primary outcomes in Control and in Intervention groups. IRRs are estimated by fitting a negative binomial regression model (with units reweighted following Hainmueller's entropy-based reweighting to account for imbalances due to attrition (1) for follow-up outcomes), and control variables selected by the Double Post Lasso method (2). Coefficients for WTP Masks are obtained by fitting an OLS regression. CI = 95% confidence interval. WTP = Willingness To Pay.

Supplement Table 3.2. Effects of Tailoring Messages on Primary Outcomes: Incidence Rate Ratios Associated With Video Tailoring, with Double Post Lasso Control Variables*

		**Black Physician * Intervention**		**AMA anti-racism* Intervention**		**Doctor racial disc. * Intervention**		**Black physician**		**AMA anti-racism**		**Covid treatment**		
Panel A	Outcome	**IRR (CI 95%)**	**p-value**	**IRR (CI 95%)**	**p-value**	**IRR (CI 95%)**	**p-value**	**IRR (CI 95%)**	**p-value**	**IRR (CI 95%)**	**p-value**	**IRR (CI 95%)**	**p-value**	**Observations**
All	Knowledge gap score	1.021 (0.972,1.072)	0.41	0.992 (0.944,1.042)	0.74	1.002 (0.979,1.025)	0.89	0.987 (0.945,1.031)	0.56	1.001 (0.959,1.046)	0.951	0.887 (0.849,0.927)	0.000	18762
	Number of links	1.048 (0.958,1.148)	0.31	1.030 (0.941,1.128)	0.52	1.026 (0.986,1.068)	0.21	0.959 (0.884,1.040)	0.32	0.971 (0.895,1.054)	0.484	1.001 (0.923,1.086)	0.974	18223
	Safety gap score	1.058 (0.964,1.160)	0.23	0.995 (0.908,1.091)	0.92	0.994 (0.953,1.037)	0.78	0.961 (0.885,1.043)	0.34	0.987 (0.909,1.071)	0.753	0.953 (0.880,1.033)	0.244	6035
	Knowledge gap follow-up	0.991 (0.911,1.077)	0.82	0.987 (0.908,1.073)	0.76	1.006 (0.968,1.045)	0.76	1.015 (0.942,1.093)	0.70	0.989 (0.918,1.066)	0.778	0.967 (0.898,1.041)	0.368	6030

© 2021 Torres C et al. *JAMA Network Open.*

Black	Knowledge gap score	1.017 (0.957,1.080)	0.59	1.001 (0.942,1.063)	0.99	1.000 (0.973,1.028)	0.98	0.985 (0.934,1.039)	0.58	0.998 (0.946,1.053)	0.946	0.935 (0.886,0.987)	0.015	9445
	Number of links	1.038 (0.926,1.165)	0.52	1.019 (0.908,1.142)	0.75	1.022 (0.971,1.075)	0.40	0.975 (0.880,1.081)	0.63	0.998 (0.901,1.106)	0.973	1.015 (0.916,1.125)	0.774	9168
	Safety gap score	1.044 (0.900,1.212)	0.57	0.977 (0.842,1.135)	0.77	1.039 (0.970,1.112)	0.28	0.988 (0.865,1.128)	0.85	0.975 (0.854,1.114)	0.713	0.935 (0.822,1.063)	0.303	2099
	Knowledge gap follow-up	0.992 (0.877,1.122)	0.90	0.992 (0.877,1.122)	0.90	1.030 (0.975,1.089)	0.29	1.017 (0.911,1.136)	0.76	0.977 (0.875,1.090)	0.676	0.971 (0.871,1.082)	0.592	2097
White	Knowledge gap score	0.998 (0.916,1.087)	0.96	0.968 (0.889,1.055)	0.46	1.001 (0.959,1.043)	0.98	1.020 (0.947,1.099)	0.60	1.010 (0.938,1.089)	0.786	0.814 (0.753,0.879)	0.000	9317
	Number of links	1.064 (0.921,1.230)	0.40	1.044 (0.903,1.206)	0.56	1.032 (0.968,1.100)	0.34	0.938 (0.824,1.067)	0.33	0.947 (0.832,1.078)	0.409	0.990 (0.870,1.126)	0.878	9055
	Safety gap score	1.065 (0.947,1.199)	0.29	1.000 (0.890,1.125)	>0.99	0.969 (0.918,1.022)	0.25	0.943 (0.849,1.048)	0.28	1.000 (0.900,1.110)	0.994	0.972 (0.876,1.078)	0.589	3936
	Knowledge gap follow-up	0.979 (0.872,1.098)	0.72	0.987 (0.881,1.107)	0.83	0.984 (0.934,1.038)	0.56	1.018 (0.919,1.127)	0.74	0.995 (0.899,1.101)	0.916	0.966 (0.873,1.070)	0.508	3933
Panel B	Outcome	**Coefficient (CI 95%)**	**p-value**	**Coefficient (CI 95%)**	**p-value**	**Coefficient (CI 95%)**	**p-value**	**Coefficient (CI 95%)**	**p-value**	**Coefficient (CI 95%)**	**p-value**	**Coefficient (CI 95%)**	**p-value**	**Observations**
All	**WTP Masks**	-0.067 (-0.721,0.587)	0.84	-0.219 (-0.873,0.436)	0.51	0.109 (-0.184,0.401)	0.47	0.051 (-0.534,0.636)	0.86	0.301 (-0.284,0.886)	0.314	0.611 (0.025,1.198)	0.041	16759
Black	**WTP Masks**	0.169 (-0.825,1.163)	0.74	0.072 (-0.923,1.067)	0.89	0.008 (-0.438,0.453)	0.97	-0.050 (-0.939,0.839)	0.91	0.099 (-0.790,0.989)	0.827	0.352 (-0.540,1.245)	0.439	7725
White	**WTP Masks**	-0.216 (-1.070,0.637)	0.62	-0.480 (-1.334,0.374)	0.27	0.152 (-0.230,0.534)	0.44	0.123 (-0.641,0.887)	0.75	0.516 (-0.248,1.279)	0.186	0.856 (0.090,1.621)	0.028	9034

*This table presents incidence rate ratios (IRR) or OLS coefficients corresponding to the subgroups in Table 3. IRRs are estimated by fitting a negative binomial regression model (with units reweighted following Hainmueller's entropy-based reweighting to account for imbalances due to attrition (1) for follow-up outcomes), and control variables selected by the Double Post Lasso method (2). CI = 95% confidence interval. WTP = Willingness To Pay.

© 2021 Torres C et al. *JAMA Network Open.*

Supplement Table 3.3. Effect of Any Message Intervention on Primary Outcomes, without reweighting*

Panel	Outcome	IRR (CI 95%)	p-value	Observations
All	Safety gap score	0.967 (0.924,1.012)	0.15	6207
	Knowledge gap follow up	0.963 (0.923,1.004)	0.08	6071
Black	Safety gap score	0.942 (0.854,1.040)	0.24	2191
	Knowledge gap follow up	0.970 (0.905,1.039)	0.38	2121
White	Safety gap score	0.976 (0.925,1.031)	0.39	4016
	Knowledge gap follow up	0.959 (0.910,1.011)	0.12	3950

*This table presents mean incidence rates of the two primary outcomes in Control and in Intervention groups, and the estimated Incidence Rate Ratio (IRR). IRRs are estimated by fitting a negative binomial regression model without reweighting. CI = 95% confidence interval.

© 2021 Torres C et al. *JAMA Network Open.*

Supplement Table 3.4. Effects of Tailoring Messages on Primary Outcomes: Incidence Rate Ratios Associated With Video Tailoring, without reweighting*

		Black Physician * Intervention		AMA anti-racism* Intervention		Doctor racial disc. * Intervention		Black physician		AMA anti-racism		Covid treatment		
Panel	Outcome	IRR (CI 95%)	p-value	IRR (CI 95%)	p-value	IRR (CI 95%)	p-value	IRR (CI 95%)	p-value	IRR (CI 95%)	p-value	IRR (CI 95%)	p-value	Observations
All	Safety gap score	1.052 (0.960,1.153)	0.28	1.010 (0.922,1.106)	0.84	0.994 (0.953,1.036)	0.76	0.966 (0.891,1.048)	0.41	0.961 (0.886,1.043)	0.342	0.942 (0.869,1.020)	0.141	6207
	Knowledge gap follow up	0.984 (0.905,1.071)	0.71	0.975 (0.897,1.061)	0.56	1.006 (0.969,1.046)	0.74	1.013 (0.940,1.092)	0.73	0.994 (0.922,1.071)	0.879	0.979 (0.909,1.055)	0.582	6071
Black	Safety gap score	1.004 (0.824,1.223)	0.97	1.015 (0.833,1.236)	0.88	1.106 (1.011,1.210)	0.03	1.003 (0.842,1.196)	0.97	0.879 (0.737,1.048)	0.151	0.887 (0.748,1.052)	0.168	2191
	Knowledge gap follow up	0.968 (0.843,1.112)	0.65	0.978 (0.852,1.123)	0.75	1.046 (0.983,1.114)	0.16	1.026 (0.906,1.160)	0.69	0.987 (0.872,1.116)	0.830	0.974 (0.862,1.101)	0.672	2121
White	Safety gap score	1.074 (0.962,1.198)	0.20	1.010 (0.905,1.126)	0.86	0.947 (0.901,0.995)	0.03	0.950 (0.862,1.048)	0.30	0.996 (0.904,1.098)	0.940	0.965 (0.876,1.063)	0.472	4016
	Knowledge gap follow up	0.993 (0.893,1.104)	0.89	0.974 (0.877,1.083)	0.63	0.983 (0.937,1.032)	0.50	1.006 (0.916,1.106)	0.89	0.998 (0.909,1.097)	0.973	0.983 (0.895,1.080)	0.723	3950

*This table presents incidence rate ratios (IRR) corresponding to the subgroups in Table 3. IRRs are estimated by fitting a negative binomial regression model without reweighting and without Double Post Lasso control variables. CI = 95% confidence interval.

© 2021 Torres C et al. *JAMA Network Open.*

Supplement Table 3.5. AMA Specific Table*

		Intervention * AMA anti-racism * AMA Black		Intervention * anti-racism AMA * AMA white		Intervention* non anti-racism AMA * AMA Black		Intervention* non anti-racism AMA * AMA white		no Intervention * anti-racism AMA * AMA Black		no Intervention * anti-racism AMA * AMA white		no Intervention * non anti-racism AMA * AMA Black	
Panel A	Outcome	IRR (CI 95%)	p-value	IRR (CI 95%)	p-value	IRR (CI 95%)	p-value	IRR (CI 95%)	p-value	IRR (CI 95%)	p-value	IRR (CI 95%)	p-value	IRR (CI 95%)	p-value
All	Knowledge gap	0.903 (0.859,0.948)	<0.001	0.889 (0.846,0.935)	<0.001	0.904 (0.861,0.950)	<0.001	0.923 (0.878,0.970)	0.001	0.997 (0.937,1.061)	0.921	1.027 (0.966,1.092)	0.397	1.045 (0.983,1.111)	0.162
	Number of links	1.010 (0.920,1.109)	0.83	1.017 (0.926,1.116)	0.73	1.005 (0.916,1.103)	0.91	1.041 (0.949,1.143)	0.40	0.928 (0.824,1.046)	0.221	0.972 (0.863,1.094)	0.633	0.965 (0.857,1.087)	0.561
	Safety count variable	0.878 (0.761,1.013)	0.08	0.863 (0.748,0.995)	0.04	0.899 (0.780,1.036)	0.14	0.909 (0.789,1.047)	0.18	0.772 (0.639,0.932)	0.007	0.998 (0.835,1.193)	0.981	0.929 (0.775,1.114)	0.428
	Knowledge gap follow up	0.936 (0.862,1.017)	0.12	0.919 (0.845,0.998)	0.05	0.956 (0.880,1.038)	0.29	0.969 (0.892,1.052)	0.46	0.957 (0.862,1.064)	0.417	1.005 (0.905,1.115)	0.930	0.995 (0.896,1.104)	0.924
Black	Knowledge gap	0.969 (0.910,1.032)	0.33	0.949 (0.891,1.011)	0.11	0.959 (0.901,1.021)	0.19	0.986 (0.926,1.049)	0.65	1.016 (0.939,1.099)	0.693	1.024 (0.946,1.107)	0.562	1.082 (1.000,1.170)	0.049
	Number of links	1.006 (0.894,1.132)	0.92	1.010 (0.898,1.136)	0.87	0.977 (0.868,1.099)	0.70	1.037 (0.922,1.166)	0.55	0.938 (0.808,1.089)	0.403	0.950 (0.818,1.102)	0.496	0.920 (0.791,1.070)	0.278
	Safety count variable	0.859 (0.657,1.123)	0.27	0.787 (0.601,1.030)	0.08	0.919 (0.704,1.198)	0.53	0.838 (0.640,1.095)	0.20	0.666 (0.467,0.949)	0.025	0.963 (0.684,1.356)	0.827	0.938 (0.668,1.316)	0.711
	Knowledge gap follow up	0.961 (0.850,1.087)	0.53	0.924 (0.817,1.046)	0.21	0.987 (0.874,1.115)	0.83	0.981 (0.868,1.109)	0.76	0.937 (0.803,1.094)	0.413	1.034 (0.886,1.207)	0.672	1.018 (0.873,1.187)	0.817
White	Knowledge gap	0.779 (0.715,0.848)	<0.001	0.778 (0.715,0.848)	<0.001	0.802 (0.737,0.873)	<0.001	0.807 (0.741,0.878)	<0.001	0.964 (0.867,1.071)	0.493	1.036 (0.933,1.149)	0.510	0.978 (0.880,1.087)	0.677

© 2021 Torres C et al. *JAMA Network Open.*

	Number of links	1.015 (0.874,1.179)	0.85	1.023 (0.881,1.188)	0.77	1.038 (0.894,1.206)	0.63	1.046 (0.901,1.215)	0.56	0.916 (0.756,1.110)	0.369	0.997 (0.824,1.207)	0.977	1.018 (0.842,1.231)	0.853
	Safety count variable	0.881 (0.748,1.038)	0.13	0.904 (0.768,1.065)	0.23	0.877 (0.744,1.033)	0.12	0.948 (0.807,1.114)	0.52	0.840 (0.677,1.043)	0.114	1.006 (0.822,1.232)	0.952	0.915 (0.742,1.129)	0.410
	Knowledge gap follow up	0.915 (0.817,1.024)	0.12	0.914 (0.817,1.023)	0.12	0.929 (0.831,1.040)	0.20	0.959 (0.858,1.072)	0.47	0.978 (0.847,1.129)	0.760	0.981 (0.852,1.129)	0.789	0.975 (0.846,1.125)	0.732
Panel B	Outcome	**Coefficient (CI 95%)**	**p-value**	**Coefficient (CI 95%)**	**p-value**	**Coefficient (CI 95%)**	**p-value**	**Coefficient (CI 95%)**	**p-value**	**Coefficient (CI 95%)**	**p-value**	**Coefficient (CI 95%)**	**p-value**	**Coefficient (CI 95%)**	**p-value**
All	**WTP Masks**	0.851 (0.154,1.548)	0.02	0.452 (-0.244,1.149)	0.20	0.415 (-0.282,1.113)	0.24	0.818 (0.121,1.515)	0.02	0.453 (-0.432,1.337)	0.316	0.145 (-0.737,1.027)	0.747	-0.050 (-0.936,0.836)	0.911
Black	**WTP Masks**	0.942 (-0.173,2.057)	0.10	0.242 (-0.871,1.355)	0.67	0.431 (-0.685,1.547)	0.45	0.623 (-0.493,1.738)	0.27	0.091 (-1.319,1.501)	0.899	-0.031 (-1.145,1.084)	0.957	-0.204 (-1.316,0.908)	0.719
White	**WTP Masks**	0.772 (-0.103,1.648)	0.08	0.632 (-0.243,1.506)	0.16	0.400 (-0.475,1.276)	0.37	0.986 (0.111,1.861)	0.03	0.764 (-0.349,1.877)	0.179				

Panel A	Outcome	**p-value joint test Intervention = 0**	**p-value joint test Intervention*AMA racism = 0**	**p-value joint test Intervention*AMA White = Intervention*AMA Black**	**p-value Intervention*AMA racism = Intervention * AMA White**	**Observations**
All	Knowledge gap	<0.001	<0.001	0.32	0.18	18762
	Number of links	0.775	0.94	0.49	0.32	18223
	Safety count variable	0.322	0.12	0.91	0.48	6035
	Knowledge gap follow up	0.163	0.14	0.70	0.21	6030
Black	Knowledge gap	0.281	0.24	0.25	0.41	9445
	Number of links	0.644	0.98	0.29	0.43	9168
	Safety count variable	0.314	0.20	0.37	0.78	2099
	Knowledge gap follow up	0.452	0.39	0.62	0.61	2097
White	Knowledge gap	<0.001	<0.001	0.98	0.24	9317
	Number of links	0.956	0.95	0.97	0.53	9055

© 2021 Torres C et al. *JAMA Network Open.*

	Safety count variable	0.357	0.32	0.32	0.18	3936
	Knowledge gap follow up	0.385	0.26	0.69	0.21	3933
Panel B	Outcome	**p-value joint test Intervention = 0**	**p-value joint test Intervention*AMA racism = 0**	**p-value joint test Intervention*AMA White = Intervention*AMA Black**	**p-value Intervention*AMA racism = Intervention * AMA White**	**Observations**
All	**WTP Masks**	0.04	0.03	0.04	0.89	16759
Black	**WTP Masks**	0.12	0.22	0.11	0.46	9034
White	**WTP Masks**					

* This table presents estimated effects on primary outcomes in different groups: by race of the AMA speaker and nature of AMA message. Effects are estimated by ordinary least squares regressions for WTP Masks, and by binomial regression for the other outcomes, with rebalancing weights obtained by the entropy weighting method for the follow up outcome. CI = 95% confidence interval. WTP = Willingness To Pay.

Supplement Table 3.6. Effect of Any Message Intervention: Follow up Sample*

		**Mean Incidence rate**				**IRR (CI 95%)**	**p-value**	**Observations**
Panel	Outcome	**Control**	**Nb obs**	**Intervention**	**Nb obs**			
All	Knowledge gap score	0.175 (0.168,0.181)	1144	0.144 (0.141,0.148)	4554	0.825 (0.783,0.868)	<0.001	5698
	Information seeking behavior	0.308 (0.287,0.328)	1120	0.324 (0.313,0.334)	4457	1.055 (0.970,1.147)	0.21	5577
Black	Knowledge gap score	0.201 (0.189,0.214)	394	0.171 (0.165,0.177)	1589	0.842 (0.784,0.906)	<0.001	1983
	Information seeking behavior	0.374 (0.338,0.410)	386	0.396 (0.378,0.415)	1557	1.049 (0.941,1.170)	0.39	1943
White	Knowledge gap score	0.160 (0.152,0.169)	750	0.130 (0.126,0.134)	2965	0.806 (0.750,0.867)	<0.001	3715
	Information seeking behavior	0.273 (0.248,0.298)	734	0.284 (0.272,0.297)	2900	1.058 (0.931,1.202)	0.39	3634
Panel	Outcome	**Mean**				**coefficient (CI 95%)**	**p-value**	**Observations**
All	**WTP Masks**	13.286 (12.780,13.792)	1115	13.564 (13.303,13.825)	4446	0.155 (-0.432,0.742)	0.61	5561
Black	**WTP Masks**	13.865 (12.995,14.735)	385	13.889 (13.422,14.355)	1554	0.075 (-0.970,1.120)	0.89	1939
White	**WTP Masks**	12.981 (12.360,13.601)	730	13.389 (13.076,13.703)	2892	0.218 (-0.478,0.915)	0.54	3622

* Sample = respondents who finished knowledge questions in follow up survey. This table presents mean values of the primary outcomes in Control and in Any Message Intervention groups, and the effect estimated by ordinary least squares regressions for WTP Masks, and by binomial regression for the other outcomes. Every regression is weighted with the Hainmueller weights. Nb obs = number of observations. WTP = Willingness To Pay.

Supplement Table 3.7. Impact of Tailoring Messages: Average Incidence Rates by Intervention*

			Black Physician		AMA anti-racism		Doctor racial discrepancy in COVID	
Panel	Outcome		**No**	**Yes**	**No**	**Yes**	**No**	**Yes**
All	**Knowledge gap**	**Mean IR**	0.215 (0.211,0.219)	0.213 (0.209,0.218)	0.217 (0.212,0.221)	0.212 (0.208,0.216)	0.214 (0.210,0.218)	0.215 (0.210,0.219)
		**Observations**	7506	7493	7494	7505	7527	7472
	**Number of links**	**Mean IR**	0.338 (0.329,0.346)	0.338 (0.330,0.347)	0.340 (0.331,0.348)	0.336 (0.328,0.345)	0.335 (0.327,0.343)	0.341 (0.332,0.349)
		**Observations**	7281	7288	7277	7292	7327	7242
	**Safety gap**	**Mean IR**	0.455 (0.441,0.469)	0.446 (0.432,0.460)	0.457 (0.443,0.471)	0.444 (0.430,0.457)	0.450 (0.436,0.464)	0.450 (0.437,0.464)
		**Observations**	2370	2453	2431	2392	2400	2423
	**Knowledge gap follow up**	**Mean IR**	0.241 (0.236,0.245)	0.241 (0.236,0.245)	0.245 (0.240,0.249)	0.237 (0.233,0.241)	0.240 (0.236,0.244)	0.241 (0.237,0.246)
		**Observations**	2369	2450	2430	2389	2398	2421
Black	**Knowledge gap**	**Mean IR**	0.297 (0.290,0.303)	0.297 (0.290,0.303)	0.299 (0.292,0.305)	0.294 (0.288,0.301)	0.295 (0.289,0.302)	0.298 (0.291,0.305)
		**Observations**	3798	3755	3784	3769	3801	3752
	**Number of links**	**Mean IR**	0.399 (0.387,0.412)	0.402 (0.389,0.414)	0.400 (0.388,0.413)	0.401 (0.388,0.413)	0.397 (0.384,0.409)	0.405 (0.392,0.417)
		**Observations**	3685	3643	3667	3661	3699	3629
	**Safety gap**	**Mean IR**	0.381 (0.356,0.406)	0.378 (0.354,0.401)	0.400 (0.375,0.424)	0.359 (0.336,0.382)	0.358 (0.335,0.382)	0.401 (0.376,0.426)
		**Observations**	809	874	846	837	848	835
	**Knowledge gap follow up**	**Mean IR**	0.259 (0.252,0.267)	0.257 (0.250,0.264)	0.262 (0.255,0.270)	0.254 (0.247,0.261)	0.252 (0.245,0.259)	0.264 (0.256,0.272)
		**Observations**	811	870	844	837	847	834
White	**Knowledge gap**	**Mean IR**	0.132 (0.128,0.136)	0.130 (0.126,0.133)	0.133 (0.129,0.137)	0.129 (0.125,0.132)	0.131 (0.127,0.135)	0.131 (0.127,0.134)
		**Observations**	3708	3738	3710	3736	3726	3720
	**Number of links**	**Mean IR**	0.274 (0.263,0.285)	0.275 (0.264,0.286)	0.278 (0.267,0.289)	0.271 (0.260,0.282)	0.272 (0.261,0.283)	0.277 (0.266,0.288)
		**Observations**	3596	3645	3610	3631	3628	3613

© 2021 Torres C et al. *JAMA Network Open.*

	**Safety gap**	**Mean IR**	0.493 (0.476,0.510)	0.483 (0.467,0.500)	0.487 (0.471,0.504)	0.489 (0.472,0.506)	0.500 (0.483,0.517)	0.476 (0.460,0.493)
		**Observations**	1561	1579	1585	1555	1552	1588
	**Knowledge gap follow up**	**Mean IR**	0.231 (0.226,0.236)	0.232 (0.227,0.237)	0.235 (0.230,0.240)	0.228 (0.222,0.233)	0.233 (0.228,0.239)	0.230 (0.225,0.235)
		**Observations**	1558	1580	1586	1552	1551	1587
All	**WTP Masks**	**Mean**	14.562 (14.332,14.791)	14.598 (14.371,14.825)	14.557 (14.327,14.786)	14.603 (14.375,14.830)	14.556 (14.330,14.781)	14.604 (14.372,14.836)
		**Observations**	6705	6694	6671	6728	6749	6650
Black	**WTP Masks**	**Mean**	16.120 (15.758,16.482)	16.135 (15.771,16.499)	16.084 (15.719,16.449)	16.171 (15.810,16.532)	16.133 (15.773,16.492)	16.122 (15.755,16.489)
		**Observations**	3117	3058	3069	3106	3133	3042
White	**WTP Masks**	**Mean**	13.207 (12.922,13.493)	13.305 (13.027,13.584)	13.255 (12.972,13.539)	13.258 (12.978,13.538)	13.190 (12.914,13.465)	13.324 (13.036,13.612)
		**Observations**	3588	3636	3602	3622	3616	3608

*This table presents number of observations and average incidence rates of knowledge gaps, information seeking behavior and safety gaps in the sample of participants who received intervention, split by whether they received a particular intervention or not. For instance, the first column shows the average incidence rate (and number of observations) for all participants that received the video message from a white physician, and the second column shows the average incidence rates (and number of observations) for all participants that received the video messages from a Black physician. 95% CI in parentheses. WTP = Willingness To Pay.

Supplement Table 3.8 Effects of Tailoring Messages on Primary Outcomes: Incidence Rate Ratios Associated With Video Tailoring, with q-values*

		**Black Physician * Intervention**		**AMA anti-racism* Intervention**		**Doctor racial disc. * Intervention**		**Black physician**		**AMA anti-racism**		**Covid treatment**		
Panel A	Outcome	**IRR (CI 95%)**	**q-value**	**IRR (CI 95%)**	**q-value**	**IRR (CI 95%)**	**q-value**	**IRR (CI 95%)**	**q-value**	**IRR (CI 95%)**	**q-value**	**IRR (CI 95%)**	**q-value**	**Observations**
All	Knowledge gap score	1.011 (0.963,1.062)	0.79	0.991 (0.943,1.041)	0.79	1.005 (0.982,1.029)	0.79	0.994 (0.952,1.038)	0.80	0.990 (0.948,1.034)	0.79	0.886 (0.848,0.927)	<0.001	18762
	Number of links	1.040 (0.946,1.142)	0.79	1.025 (0.933,1.126)	0.79	1.023 (0.981,1.066)	0.79	0.963 (0.885,1.048)	0.79	0.966 (0.888,1.051)	0.79	1.010 (0.928,1.098)	0.82	18223
	Safety gap score	1.037 (0.945,1.139)	0.79	1.016 (0.925,1.116)	0.79	1.008 (0.965,1.051)	0.79	0.968 (0.890,1.052)	0.79	0.946 (0.871,1.029)	0.79	0.931 (0.858,1.010)	0.79	6035
	Knowledge gap follow-up	0.981 (0.902,1.066)	0.79	0.980 (0.902,1.065)	0.79	1.014 (0.976,1.053)	0.79	1.014 (0.941,1.092)	0.79	0.983 (0.913,1.059)	0.79	0.967 (0.899,1.041)	0.79	6030

© 2021 Torres C et al. *JAMA Network Open.*

Black	Knowledge gap score	1.005 (0.944,1.069)	0.99	1.007 (0.946,1.071)	0.99	1.009 (0.980,1.038)	0.99	0.996 (0.943,1.053)	0.99	0.980 (0.927,1.036)	0.99	0.928 (0.878,0.981)	0.13	9445
	Number of links	1.035 (0.919,1.165)	0.99	1.019 (0.905,1.147)	0.99	1.021 (0.968,1.076)	0.99	0.972 (0.874,1.081)	0.99	0.983 (0.884,1.093)	0.99	1.020 (0.918,1.135)	0.99	9168
	Safety gap score	1.008 (0.846,1.202)	0.99	1.033 (0.867,1.232)	0.99	1.121 (1.035,1.214)	0.13	0.979 (0.837,1.145)	0.99	0.868 (0.742,1.015)	0.58	0.880 (0.756,1.023)	0.58	2099
	Knowledge gap follow-up	0.973 (0.861,1.100)	0.99	0.985 (0.872,1.113)	0.99	1.051 (0.994,1.111)	0.58	1.016 (0.911,1.133)	0.99	0.974 (0.873,1.086)	0.99	0.963 (0.865,1.072)	0.99	2097
White	Knowledge gap score	1.027 (0.942,1.118)	0.91	0.957 (0.879,1.043)	0.91	0.997 (0.956,1.039)	0.95	0.988 (0.917,1.064)	0.95	1.011 (0.938,1.089)	0.95	0.804 (0.745,0.869)	<0.001	9317
	Number of links	1.046 (0.899,1.217)	0.91	1.033 (0.888,1.201)	0.95	1.026 (0.959,1.097)	0.91	0.952 (0.831,1.090)	0.91	0.947 (0.827,1.084)	0.91	0.997 (0.871,1.141)	0.96	9055
	Safety gap score	1.058 (0.941,1.189)	0.91	1.008 (0.897,1.133)	0.95	0.939 (0.890,0.991)	0.21	0.960 (0.865,1.066)	0.91	0.996 (0.897,1.105)	0.96	0.962 (0.868,1.067)	0.91	3936
	Knowledge gap follow-up	0.986 (0.880,1.105)	0.95	0.976 (0.871,1.094)	0.95	0.982 (0.932,1.035)	0.91	1.012 (0.915,1.120)	0.95	0.992 (0.896,1.097)	0.95	0.972 (0.878,1.075)	0.91	3933
Panel B	Outcome	**Coefficient (CI 95%)**	**q-value**	**Coefficient (CI 95%)**	**q-value**	**Coefficient (CI 95%)**	**q-value**	**Coefficient (CI 95%)**	**q-value**	**Coefficient (CI 95%)**	**q-value**	**Coefficient (CI 95%)**	**q-value**	**Observations**
All	**WTP Masks**	-0.211 (-0.912,0.489)	0.79	-0.291 (-0.992,0.409)	0.79	0.072 (-0.241,0.386)	0.79	0.174 (-0.453,0.800)	0.79	0.324 (-0.303,0.950)	0.79	0.713 (0.085,1.342)	0.39	16759
Black	**WTP Masks**	0.036 (-1.079,1.150)	0.99	-0.091 (-1.206,1.023)	0.99	0.001 (-0.498,0.501)	0.99	-0.013 (-1.009,0.984)	0.99	0.153 (-0.844,1.150)	0.99	0.445 (-0.555,1.446)	0.99	7725
White	**WTP Masks**	-0.425 (-1.309,0.459)	0.91	-0.464 (-1.348,0.420)	0.91	0.133 (-0.263,0.529)	0.91	0.334 (-0.457,1.124)	0.91	0.471 (-0.319,1.262)	0.91	0.944 (0.151,1.736)	0.21	9034

*This table presents incidence rate ratios (IRR) or OLS coefficients corresponding to the subgroups in Table 3. IRRs are estimated by fitting a negative binomial regression model (with units reweighted following Hainmueller's entropy-based reweighting to account for imbalances due to attrition (1) for follow-up outcomes). q-values are reported accounting for the different outcomes and coefficients in each panel. CI = 95% confidence interval. WTP = Willingness To Pay.

© 2021 Torres C et al. *JAMA Network Open.*

eFigure 1. Full Study Flowchart

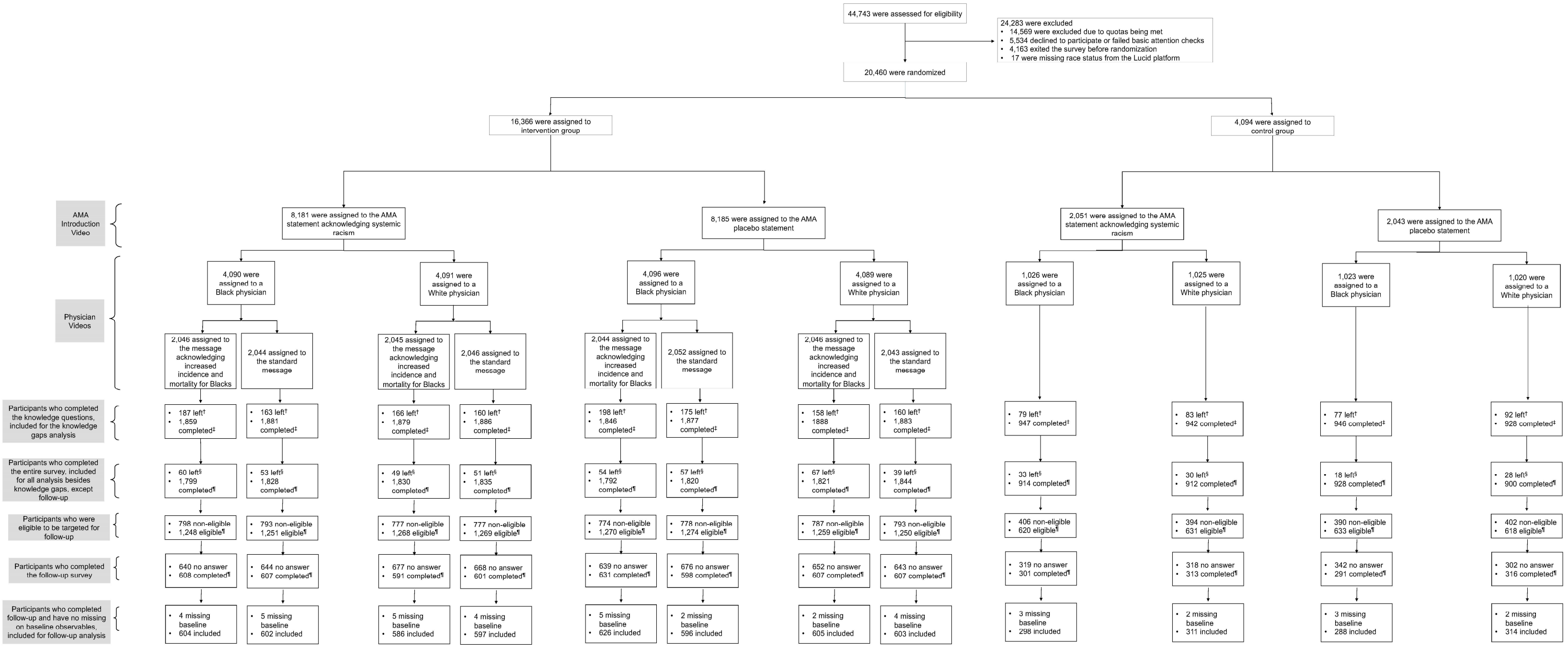

© 2021 Torres C et al. *JAMA Network Open.*

eFigure 2. Distribution of the Safety Gap Score in the Control and Intervention Groups[a]

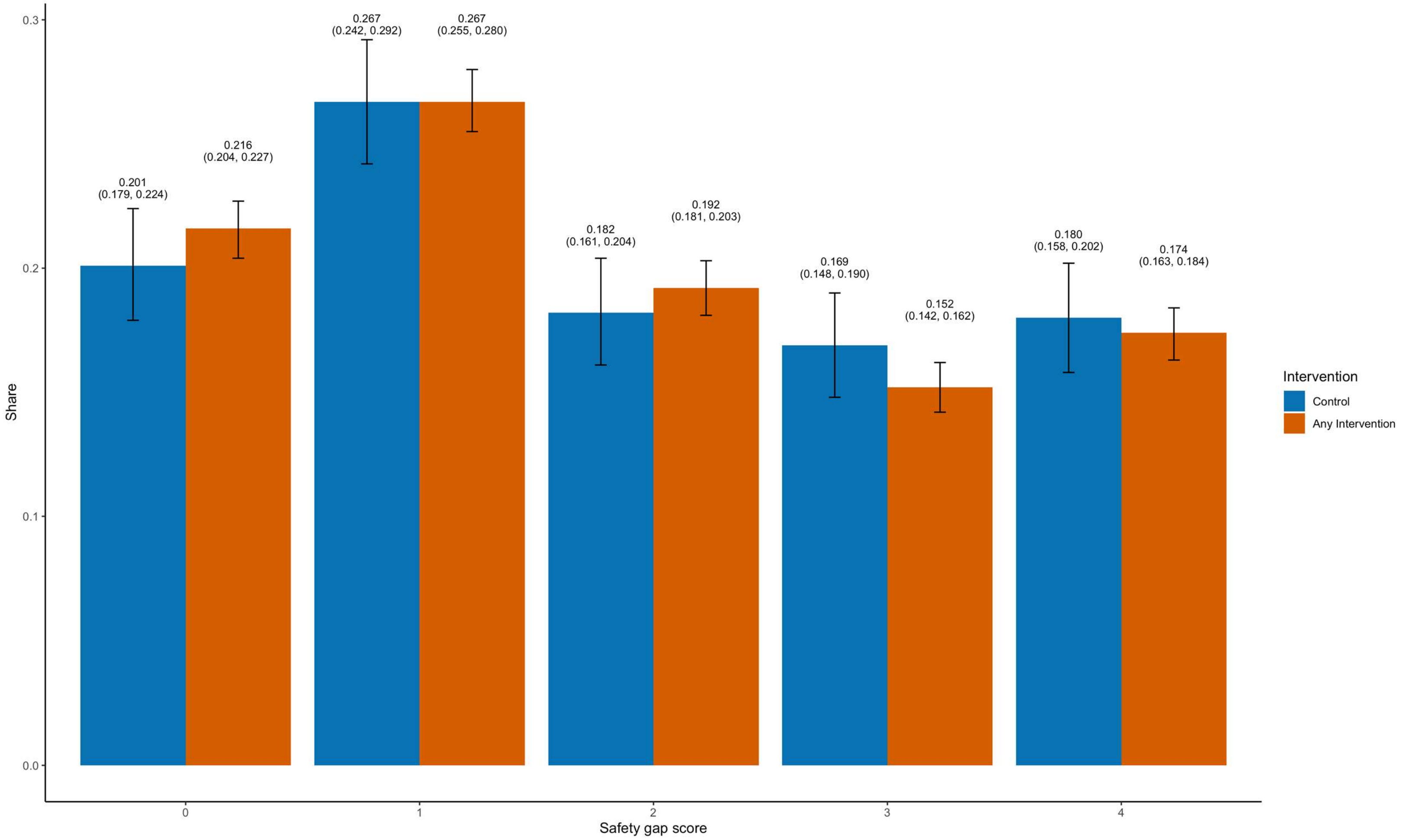

[a] Figure 2 shows the fraction of participants with a safety gap score of 0 (perfect answers), 1, 2, 3, or 4 (with confidence intervals in parentheses below each fraction) in the control group and in the intervention group. A Kolmogorov Smirnov test of the equality of the distribution has a value of 0.023486 (p=0.6593). This outcome was measured a few days after the initial intervention, for a subsample that was eligible for follow up and could be tracked. Participants were asked about how often they engaged in four behaviors of interest (1. If they wore a mask indoors, 2. If they wore a mask outdoors, 3. If they washed their hands, and 4. If they followed social distancing guidelines). Failure to answer “always” to each of these practices added one point to the safety gap count. The safety gap count is an integer that can have values from 0 (all safety practices) to 4 (no safety practice).

© 2021 Torres C et al. *JAMA Network Open.*

eTable 1. Balance and Attrition

eTable 1a. Balance Table*

	Intervention		Black physician		AMA anti-racism		Doctor racial discrepancy in COVID-19	
	**Mean diff. adjusted**	**KS p-value adjusted**	**Mean diff. adjusted**	**KS p-value adjusted**	**Mean diff. adjusted**	**KS p-value adjusted**	**Mean diff. adjusted**	**KS p-value adjusted**
**Panel A**								
Total balanced	375	375	375	375	375	375	375	375
Total unbalanced	0	0	0	0	0	0	0	0
Control size	3544		8850		8838		10602	
Treatment size	14145		8839		8851		7087	
**Panel B**								
Total balanced	375	375	375	375	375	375	375	375
Total unbalanced	0	0	0	0	0	0	0	0
Control size	3239		8082		8039		9696	
Treatment size	12876		8033		8076		6419	
**Panel C**								
Total balanced	375	375	375	375	375	375	375	375
Total unbalanced	0	0	0	0	0	0	0	0
Control size	3135		7824		7785		9391	
Treatment size	12447		7758		7797		6191	
**Panel D**								
Total balanced	375	375	375	375	375	375	375	375
Total unbalanced	0	0	0	0	0	0	0	0
Control size	1215		3018		3038		3618	
Treatment size	4829		3026		3006		2426	

*This table presents balance diagnostics performed using the R package COBALT. The standardized mean difference and the p-value of Kolmogorov Smirnov tests are computed for each baseline covariate and each interaction between two baseline covariates. Intervention column tests for balance between the group

© 2021 Torres C et al. *JAMA Network Open.*

that received the intervention and the control group. Other columns test for balance between the group that received messages tailored in a specific way relative to the group which did not receive the tailoring under consideration. Mean diff. adjusted = Mean difference adjusted. KS p-value adjusted = Kolmogorov Smirnov p-value adjusted. Total balanced refers to the number of variables and interactions of two covariates which are found to be balanced by the criteria for mean and KS test p-value. Total balanced refers to the number of variables and interactions of two covariates found to be imbalanced. Panel A includes all participants who were randomized and answered all baseline questions. Panel B includes the subsample of participants who completed the knowledge questions. Panel C includes the subsample of participants who completed links questions. Panel D includes the subsample of participants who completed the knowledge questions in the follow up survey. The 375 variables include: baseline variables, interactions between baseline variables and propensity score.

Supplement Table 1b. Attrition Table*

	Intervention	Control	Difference (KS-test p-value)	AMA anti-racism	AMA Placebo	Difference (KS-test p-value)
Sample size at first randomization	16366	4094		10232	10228	
Number attrited by knowledge questions	1349	327	1.00	822	854	1.00
% attrited	8.2%	8.0%		8.0%	8.3%	
Number attrited by links	1797	440	1.00	1114	1123	1.00
% attrited	11.0%	10.7%		10.9%	11.0%	
	Intervention	Control	Difference (KS-test p-value)	AMA anti-racism	AMA Placebo	Difference (KS-test p-value)
Number of people we attempted to follow up with	10089	2502		6287	6304	
Number attrited by knowledge questions	5229	1277	1.00	3258	3248	1.00
% attrited	51.8%	51.0%		51.8%	51.5%	

*This table presents attrition diagnostics to compare attrition in the Intervention group vs attrition in the control group and also attrition in the group which received the AMA Racism Statement vs attrition in the group which received the placebo statement. It compares the attrition rates by different stages of the survey: by knowledge questions, by links and by knowledge questions in the follow up survey. The columns “Difference (KS-test) p-value” give the p-value of the Kolmogorov-Smirnov test between the attrition distributions of both groups (Intervention vs Control or AMA Racism Statement vs AMA Placebo).

© 2021 Torres C et al. *JAMA Network Open.*

eTable 2. Outcomes by Subgroup

Supplement Table 2a. Analyses of Outcomes by Sex*

	Control		Intervention		Male*Intervention		Female*Intervention		Difference	
Outcome	**Male IR**	**Female IR**	**Male IR**	**Female IR**	**IRR (CI 95%)**	**p-value**	**IRR (CI 95%)**	**p-value**	**p-value**	No obs
Knowledge gap score	0.247 (0.238,0.255)	0.236 (0.228,0.244)	0.221 (0.216,0.225)	0.209 (0.205,0.213)	0.891 (0.858,0.924)	<0.001	0.889 (0.860,0.919)	<0.001	0.93	18762
Information seeking behavior	0.331 (0.313,0.350)	0.311 (0.296,0.327)	0.351 (0.342,0.361)	0.328 (0.320,0.336)	1.058 (0.986,1.136)	0.12	1.050 (0.986,1.118)	0.13	0.87	18223
Safety gap score	0.517 (0.488,0.545)	0.418 (0.392,0.444)	0.505 (0.491,0.520)	0.400 (0.387,0.413)	0.968 (0.906,1.035)	0.35	0.949 (0.889,1.013)	0.12	0.67	6035
Knowledge gap follow up	0.260 (0.250,0.270)	0.240 (0.233,0.248)	0.254 (0.249,0.259)	0.229 (0.225,0.232)	0.962 (0.905,1.022)	0.21	0.950 (0.897,1.006)	0.08	0.77	6030
Outcome	**Male mean**	**Female mean**	**Male mean**	**Female mean**	**Coefficient (CI 95%)**	**p-value**	**Coefficient (CI 95%)**	**p-value**	**p-value**	**Observations**
**WTP Masks**	14.018 (13.523,14.513)	14.115 (13.722,14.509)	14.229 (13.978,14.481)	14.857 (14.647,15.067)	0.189 (-0.338,0.715)	0.48	0.741 (0.272,1.211)	0.002	0.13	16759
**Donation to COVID Charity**	512.765 (500.311,525.219)	522.990 (512.613,533.367)	534.013 (527.602,540.423)	541.514 (536.256,546.773)	21.130 (7.610,34.651)	0.002	18.123 (6.044,30.202)	0.003	0.75	16634
**Donation to Black Charity**	425.873 (413.705,438.042)	444.541 (434.707,454.374)	446.896 (440.850,452.941)	453.075 (447.998,458.152)	20.552 (7.931,33.173)	0.001	8.788 (-2.473,20.049)	0.13	0.17	16783
Outcome	**Male Odds**	**Female Odds**	**Male Odds**	**Female Odds**	**Odds ratio (CI 95%)**	**p-value**	**Odds ratio (CI 95%)**	**p-value**	**p-value**	**Observations**
**Trust in Federal response**	0.259 (0.237,0.281)	0.172 (0.155,0.188)	0.247 (0.236,0.258)	0.187 (0.178,0.195)	0.935 (0.820,1.067)	0.32	1.123 (0.981,1.286)	0.09	0.06	17994
**Trust in Local response**	0.365 (0.342,0.389)	0.312 (0.292,0.332)	0.368 (0.356,0.380)	0.318 (0.308,0.328)	1.012 (0.901,1.137)	0.84	1.027 (0.922,1.144)	0.63	0.86	18132

* This table presents incidence rates (or means/Odds) and incidence rate ratios (or coefficients/Odds ratios) from the intervention, disaggregated by sex. P-values correspond to a test that the IRR/Odds ratios for female and male participants is equal to 1 (or 0 for OLS coefficients), and to a test that the IRR (or coefficients/Odds ratios) for female is different from the IRR (or coefficients/Odds ratios) for male, in order to test for heterogeneous effects. IRRs are estimated by fitting a negative binomial regression model (with units reweighted following Hainmueller's entropy-based reweighting to account for imbalances due to attrition for follow up outcomes) (1). Odds ratio are estimated by fitting a logistic regression. Coefficients for WTP Masks and Donation outcomes are obtained by fitting an OLS regression. CI = 95% confidence interval. WTP = Willingness To Pay.

© 2021 Torres C et al. *JAMA Network Open.*

Supplement Table 2b. Analyses of Outcomes by Education*

	Control		Intervention		not HS*Intervention		HS*Intervention		Difference	
Outcome	**not HS IR**	**HS IR**	**not HS IR**	**HS IR**	**IRR (CI 95%)**	**p-value**	**IRR (CI 95%)**	**p-value**	**p-value**	**Observations**
Knowledge gap score	0.281 (0.265,0.297)	0.234 (0.228,0.241)	0.272 (0.263,0.281)	0.206 (0.202,0.209)	0.988 (0.929,1.052)	0.71	0.873 (0.850,0.896)	<0.001	<0.001	18762
Information seeking behavior	0.403 (0.371,0.434)	0.308 (0.295,0.320)	0.400 (0.384,0.416)	0.329 (0.322,0.335)	0.995 (0.878,1.127)	0.94	1.065 (1.013,1.121)	0.02	0.32	18223
Safety gap score	0.481 (0.422,0.540)	0.462 (0.441,0.483)	0.453 (0.424,0.482)	0.450 (0.439,0.460)	0.932 (0.829,1.047)	0.24	0.964 (0.916,1.015)	0.16	0.60	6035
Knowledge gap follow up	0.290 (0.270,0.309)	0.243 (0.237,0.250)	0.266 (0.256,0.276)	0.237 (0.234,0.240)	0.897 (0.813,0.990)	0.03	0.969 (0.926,1.015)	0.19	0.16	6030
Outcome	**not HS mean**	**HS mean**	**not HS mean**	**HS mean**	**Coefficient (CI 95%)**	**p-value**	**Coefficient (CI 95%)**	**p-value**	**p-value**	**Observations**
**WTP Masks**	15.914 (15.029,16.799)	13.768 (13.439,14.097)	16.749 (16.275,17.222)	14.224 (14.053,14.394)	0.914 (-0.013,1.841)	0.05	0.430 (0.054,0.807)	0.03	0.34	16759
**Donation to COVID Charity**	528.375 (507.876,548.874)	516.807 (508.132,525.482)	533.383 (522.626,544.140)	538.986 (534.576,543.396)	4.071 (-19.836,27.978)	0.74	22.055 (12.330,31.780)	<0.001	0.17	16634
**Donation to Black Charity**	459.013 (439.953,478.073)	432.534 (424.151,440.916)	481.730 (471.351,492.109)	445.212 (441.017,449.406)	20.515 (-1.886,42.916)	0.07	12.936 (3.873,22.000)	0.005	0.54	16783
Outcome	**not HS Odds**	**HS Odds**	**not HS Odds**	**not HS Odds**	**Odds ratio (CI 95%)**	**p-value**	**Odds ratio (CI 95%)**	**p-value**	**p-value**	**Observations**
**Trust in Federal response**	0.275 (0.235,0.315)	0.200 (0.186,0.214)	0.263 (0.243,0.283)	0.205 (0.198,0.212)	0.952 (0.754,1.204)	0.68	1.039 (0.937,1.152)	0.47	0.51	17994
**Trust in Local response**	0.328 (0.286,0.370)	0.336 (0.320,0.353)	0.343 (0.321,0.364)	0.339 (0.331,0.347)	1.070 (0.862,1.330)	0.54	1.012 (0.930,1.102)	0.78	0.64	18132

* This table presents incidence rates (or means/Odds) and incidence rate ratios (or coefficients/Odds ratios) from the intervention, disaggregated by education. P-values correspond to tests that the IRR/Odds ratios for low education (i.e. participants with less than high school education) and high education (i.e. participants

© 2021 Torres C et al. *JAMA Network Open.*

with at least high school education) is equal to 1 (or 0 for OLS coefficients), and to a test that the IRR (or coefficients/Odds ratios) for participants with low education is different from the IRR (or coefficients/Odds ratios) for participants with high education levels, in order to test for heterogeneous effects. IRRs are estimated by fitting a negative binomial regression model (with units reweighted following Hainmueller's entropy-based reweighting to account for imbalances due to attrition for follow up outcomes) (1). Odds ratio are estimated by fitting a logistic regression. Coefficients for WTP Masks and Donation outcomes are obtained by fitting an OLS regression. CI = 95% confidence interval. WTP = Willingness To Pay.

Supplement Table 2c. Analyses of Outcomes by Race*

	**Control**		**Intervention**		**White*Intervention**		**Black*Intervention**		**Difference**	
Outcome	**White IR**	**Black IR**	**White IR**	**Black IR**	**IRR (CI 95%)**	**p-value**	**IRR (CI 95%)**	**p-value**	**p-value**	**Observations**
Knowledge gap score	0.165 (0.159,0.170)	0.316 (0.307,0.324)	0.131 (0.128,0.133)	0.297 (0.292,0.301)	0.796 (0.763,0.831)	<0.001	0.938 (0.910,0.967)	<0.001	<0.001	18762
Information seeking behavior	0.261 (0.245,0.277)	0.378 (0.361,0.396)	0.275 (0.267,0.282)	0.401 (0.392,0.409)	1.049 (0.978,1.124)	0.18	1.058 (0.993,1.128)	0.08	0.85	18223
Safety gap score	0.501 (0.477,0.525)	0.395 (0.361,0.429)	0.488 (0.476,0.500)	0.379 (0.362,0.396)	0.963 (0.907,1.022)	0.21	0.952 (0.883,1.027)	0.20	0.82	6035
Knowledge gap follow up	0.242 (0.234,0.249)	0.265 (0.254,0.277)	0.231 (0.228,0.235)	0.258 (0.253,0.263)	0.945 (0.893,1.001)	0.05	0.967 (0.910,1.028)	0.28	0.59	6030
Outcome	**White mean**	**Black mean**	**White mean**	**Black mean**	**Coefficient (CI 95%)**	**p-value**	**Coefficient (CI 95%)**	**p-value**	**p-value**	**Observations**
**WTP Masks**	12.676 (12.285,13.066)	15.703 (15.222,16.184)	13.257 (13.057,13.456)	16.128 (15.871,16.384)	0.567 (0.089,1.044)	0.02	0.417 (-0.099,0.933)	0.11	0.68	16759
**Donation to COVID Charity**	515.532 (504.095,526.968)	521.871 (510.840,532.902)	533.816 (528.027,539.605)	543.363 (537.670,549.056)	17.788 (5.531,30.045)	0.004	21.511 (8.227,34.795)	0.002	0.69	16634
**Donation to Black Charity**	393.098 (382.313,403.882)	486.046 (475.641,496.451)	403.688 (398.353,409.024)	504.359 (498.965,509.754)	10.266 (-1.212,21.745)	0.08	18.346 (6.015,30.678)	0.004	0.35	16783
Outcome	**White Odds**	**Black Odds**	**White Odds**	**Black Odds**	**Odds ratio (CI 95%)**	**p-value**	**Odds ratio (CI 95%)**	**p-value**	**p-value**	**Observations**
**Trust in Federal response**	0.277 (0.256,0.297)	0.142 (0.126,0.158)	0.280 (0.270,0.291)	0.144 (0.136,0.152)	1.026 (0.911,1.157)	0.67	1.021 (0.876,1.189)	0.79	0.95	17994

© 2021 Torres C et al. *JAMA Network Open.*

Trust in Local response	0.402 (0.380,0.425)	0.268 (0.248,0.289)	0.418 (0.406,0.429)	0.262 (0.251,0.272)	1.064 (0.958,1.183)	0.25	0.965 (0.857,1.087)	0.56	0.23	18132

*This table presents incidence rates (or means/Odds) and incidence rate ratios (or coefficients/Odds ratios) from the intervention, disaggregated by race. P-values correspond to tests that the IRR/Odds ratio for Black and white is equal to 1 (0 for OLS coefficients), and to a test that the IRR (or coefficient/Odds ratio) for Black participants is different from the IRR (or coefficient/Odds ratio) for white participants, in order to test for heterogeneous effects. IRRs are estimated by fitting a negative binomial regression model (with units reweighted following Hainmueller's entropy-based reweighting to account for imbalances due to attrition for follow up outcomes) (1). Odds ratio are estimated by fitting a logistic regression. Coefficients for WTP Masks and Donation outcomes are obtained by fitting an OLS regression. CI = 95% confidence interval. WTP = Willingness To Pay.

Supplement Table 2d. Analyses of Outcomes by Prior Belief about the ratio of black mortality to white mortality from COVID-19*

	Control		Intervention		(Prior belief <4)*Intervention		(Prior belief >=4)*Intervention		Difference	
Outcome, Panel = All	**IR Prior belief<4**	**IR Prior belief>= 4**	**IR Prior belief<4 IR**	**IR Prior belief>= 4**	**IRR (CI 95%)**	**p-value**	**IRR (CI 95%)**	**p-value**	**p-value**	**Observations**
Knowledge gap score	0.250 (0.244,0.257)	0.213 (0.203,0.223)	0.227 (0.223,0.231)	0.181 (0.176,0.186)	0.902 (0.877,0.928)	<0.001	0.856 (0.813,0.900)	<0.001	0.08	18762
Information seeking behavior	0.309 (0.295,0.323)	0.352 (0.329,0.374)	0.323 (0.315,0.330)	0.378 (0.367,0.389)	1.042 (0.986,1.102)	0.14	1.075 (0.983,1.175)	0.11	0.57	18223
Safety gap score	0.483 (0.459,0.507)	0.423 (0.390,0.457)	0.475 (0.463,0.487)	0.394 (0.377,0.410)	0.977 (0.925,1.031)	0.40	0.908 (0.829,0.994)	0.04	0.17	6035
Knowledge gap follow up	0.255 (0.247,0.262)	0.238 (0.228,0.248)	0.244 (0.241,0.248)	0.232 (0.227,0.238)	0.948 (0.903,0.996)	0.03	0.975 (0.900,1.055)	0.53	0.57	6030
Outcome, Panel = All	**Mean Prior belief<4**	**Mean Prior belief>=4**	**Mean Prior belief<4**	**Mean Prior belief>=4**	**Coefficient (CI 95%)**	**p-value**	**Coefficient (CI 95%)**	**p-value**	**p-value**	**Observations**
**WTP Masks**	13.589 (13.222,13.955)	15.295 (14.722,15.869)	14.182 (13.988,14.376)	15.524 (15.233,15.815)	0.583 (0.169,0.998)	0.006	0.243 (-0.411,0.898)	0.47	0.39	16759
**Donation to COVID Charity**	506.127 (496.507,515.748)	549.164 (535.057,563.271)	523.174 (518.229,528.118)	573.446 (566.368,580.525)	17.004 (6.355,27.654)	0.002	23.881 (7.167,40.595)	0.005	0.50	16634

**Donation to Black Charity**	422.973 (413.570,432.375)	469.883 (457.037,482.728)	438.246 (433.473,443.019)	479.226 (472.665,485.788)	14.974 (5.057,24.892)	0.003	10.200 (-5.486,25.885)	0.20	0.61	16783
Outcome, Panel = All	**Odds Prior belief<4**	**Odds Prior belief>=4**	**Odds Prior belief<4**	**Odds Prior belief>=4**	**Odds ratio (CI 95%)**	**p-value**	**Odds ratio (CI 95%)**	**p-value**	**p-value**	**Observations**
**Posterior belief < 4**	0.736 (0.707,0.765)	0.148 (0.126,0.171)	0.589 (0.576,0.603)	0.197 (0.184,0.209)	0.510 (0.434,0.599)	<0.001	1.405 (1.154,1.711)	0.001	<0.001	10688
**Posterior belief >= 4**	0.264 (0.235,0.293)	0.852 (0.829,0.874)	0.411 (0.397,0.424)	0.803 (0.791,0.816)	1.961 (1.671,2.302)	<0.001	0.712 (0.584,0.867)	0.001	<0.001	10688
**Trust in Federal response**	0.226 (0.211,0.242)	0.163 (0.140,0.187)	0.237 (0.229,0.245)	0.150 (0.139,0.161)	1.071 (0.962,1.192)	0.21	0.895 (0.734,1.093)	0.87	0.12	17994
**Trust in Local response**	0.326 (0.309,0.344)	0.358 (0.328,0.389)	0.330 (0.321,0.339)	0.363 (0.348,0.378)	1.020 (0.929,1.119)	0.68	1.014 (0.872,1.179)	0.86	0.95	18132
Outcome, Panel = Black	**IR Prior belief<4**	**IR Prior belief>=4**	**IR Prior belief<4**	**IR Prior belief>=4**	**IRR (CI 95%)**	**p-value**	**IRR (CI 95%)**	**p-value**	**p-value**	**Observations**
Knowledge gap score	0.333 (0.323,0.343)	0.268 (0.252,0.284)	0.317 (0.311,0.322)	0.244 (0.235,0.252)	0.949 (0.917,0.982)	0.003	0.907 (0.851,0.966)	0.002	0.22	9445
Information seeking behavior	0.373 (0.352,0.394)	0.393 (0.363,0.423)	0.387 (0.376,0.397)	0.437 (0.421,0.452)	1.036 (0.967,1.111)	0.32	1.112 (0.994,1.244)	0.06	0.30	9168
Safety gap score	0.388 (0.346,0.431)	0.410 (0.354,0.466)	0.391 (0.369,0.412)	0.356 (0.328,0.384)	0.999 (0.898,1.111)	0.98	0.850 (0.726,0.994)	0.04	0.09	2099
Knowledge gap follow up	0.272 (0.258,0.285)	0.252 (0.231,0.273)	0.263 (0.257,0.270)	0.247 (0.239,0.255)	0.961 (0.894,1.034)	0.29	0.980 (0.876,1.097)	0.73	0.78	2097
Outcome, Panel = Black	**Mean Prior belief<4**	**Mean Prior belief>=4**	**Mean Prior belief<4**	**Mean Prior belief>=4**	**Coefficient (CI 95%)**	**p-value**	**Coefficient (CI 95%)**	**p-value**	**p-value**	**Observations**
**WTP Masks**	15.501 (14.918,16.083)	16.151 (15.299,17.004)	15.896 (15.581,16.212)	16.623 (16.183,17.063)	0.407 (-0.265,1.079)	0.24	0.434 (-0.563,1.431)	0.39	0.97	7725
**Donation to COVID Charity**	515.721 (502.168,529.275)	535.127 (516.235,554.019)	535.897 (528.855,542.939)	558.954 (549.349,568.558)	20.221 (4.965,35.477)	0.009	23.753 (1.423,46.083)	0.04	0.80	7627

© 2021 Torres C et al. *JAMA Network Open.*

**Donation to Black Charity**	483.659 (470.807,496.512)	491.372 (473.811,508.933)	499.788 (493.118,506.459)	514.296 (505.204,523.387)	16.146 (1.749,30.543)	0.03	22.992 (1.540,44.444)	0.04	0.60	7781
Outcome, Panel = Black	**odds Prior belief<4**	**odds Prior belief>= 4**	**odds Prior belief<4 IR**	**odds Prior belief>= 4**	**Odds ratio (CI 95%)**	**p-value**	**Odds ratio (CI 95%)**	**p-value**	**p-value**	**Observations**
**Posterior belief < 4**	0.674 (0.632,0.717)	0.197 (0.161,0.233)	0.598 (0.578,0.617)	0.224 (0.205,0.242)	0.712 (0.577,0.879)	0.002	1.170 (0.909,1.507)	0.22	0.003	5178
**Posterior belief >= 4**	0.326 (0.283,0.368)	0.803 (0.767,0.839)	0.402 (0.383,0.422)	0.776 (0.758,0.795)	1.404 (1.137,1.734)	0.002	0.855 (0.664,1.100)	0.22	0.003	5178
**Trust in Federal response**	0.156 (0.136,0.175)	0.105 (0.078,0.132)	0.156 (0.146,0.166)	0.114 (0.100,0.127)	1.003 (0.843,1.193)	0.98	1.096 (0.791,1.518)	0.58	0.64	8943
**Trust in Local response**	0.261 (0.237,0.285)	0.288 (0.248,0.328)	0.250 (0.238,0.262)	0.291 (0.271,0.311)	0.941 (0.817,1.084)	0.40	1.021 (0.817,1.276)	0.85	0.55	9081
Outcome, Panel = White	**IR Prior belief<4**	**IR Prior belief>= 4**	**IR Prior belief<4 IR**	**IR Prior belief>= 4**	**IRR (CI 95%)**	**p-value**	**IRR (CI 95%)**	**p-value**	**p-value**	**Observations**
Knowledge gap score	0.168 (0.161,0.174)	0.156 (0.145,0.166)	0.135 (0.132,0.139)	0.119 (0.114,0.123)	0.808 (0.770,0.849)	<0.001	0.762 (0.699,0.830)	<0.001	0.24	9317
Information seeking behavior	0.245 (0.227,0.262)	0.308 (0.275,0.340)	0.257 (0.248,0.267)	0.319 (0.304,0.334)	1.050 (0.961,1.147)	0.28	1.032 (0.895,1.191)	0.66	0.85	9055
Safety gap score	0.530 (0.502,0.559)	0.431 (0.389,0.473)	0.518 (0.504,0.532)	0.417 (0.396,0.437)	0.965 (0.903,1.031)	0.29	0.955 (0.846,1.078)	0.46	0.88	3936
Knowledge gap follow up	0.246 (0.237,0.256)	0.230 (0.219,0.240)	0.235 (0.230,0.239)	0.224 (0.217,0.230)	0.937 (0.877,1.001)	0.05	0.969 (0.866,1.085)	0.59	0.62	3933
Outcome, Panel = White	**Mean Prior belief<4**	**Mean Prior belief>=4**	**Mean Prior belief<4**	**Mean Prior belief>=4**	**Coefficient (CI 95%)**	**p-value**	**Coefficient (CI 95%)**	**p-value**	**p-value**	**Observations**
**WTP Masks**	12.065 (11.614,12.516)	14.417 (13.660,15.174)	12.799 (12.565,13.033)	14.447 (14.071,14.823)	0.724 (0.210,1.237)	0.006	0.027 (-0.831,0.886)	0.95	0.17	9034
**Donation to COVID Charity**	498.573 (485.098,512.047)	563.738 (542.774,584.702)	513.108 (506.234,519.982)	587.743 (577.389,598.097)	14.464 (-0.327,29.255)	0.06	23.356 (-1.365,48.077)	0.06	0.55	9007

© 2021 Torres C et al. *JAMA Network Open.*

Donation to Black Charity	373.841 (360.965,386.718)	447.753 (429.162,466.344)	387.823 (381.391,394.255)	444.893 (435.682,454.104)	14.042 (0.397,27.687)	0.04	-3.658 (-26.445,19.128)	0.75	0.19	9002
Outcome, Panel = White	**odds Prior belief<4**	**odds Prior belief>= 4**	**odds Prior belief<4 IR**	**odds Prior belief>= 4**	**Odds ratio (CI 95%)**	**p-value**	**Odds ratio (CI 95%)**	**p-value**	**p-value**	**Observations**
**Posterior belief < 4**	0.805 (0.767,0.843)	0.100 (0.073,0.127)	0.582 (0.563,0.601)	0.171 (0.154,0.187)	0.330 (0.256,0.425)	<0.001	1.853 (1.341,2.561)	<0.001	<0.001	5510
**Posterior belief >= 4**	0.195 (0.157,0.233)	0.900 (0.873,0.927)	0.418 (0.399,0.437)	0.829 (0.813,0.846)	3.033 (2.352,3.912)	<0.001	0.540 (0.391,0.746)	<0.001	<0.001	5510
**Trust in Federal response**	0.295 (0.271,0.319)	0.225 (0.187,0.262)	0.316 (0.304,0.329)	0.186 (0.169,0.203)	1.114 (0.973,1.277)	0.12	0.790 (0.614,1.017)	0.07	0.02	9051
**Trust in Local response**	0.391 (0.365,0.418)	0.433 (0.388,0.478)	0.411 (0.397,0.424)	0.436 (0.414,0.457)	1.083 (0.957,1.225)	0.21	1.012 (0.825,1.241)	0.91	0.58	9051

*This table presents incidence rates (or means/Odds) and incidence rate ratios (or coefficients/Odds ratios) from the intervention, disaggregated by prior belief ("Black people are less than 4 times as likely to die from COVID 19" or "Black people are more than 4 times as likely to die from COVID 19). P-values correspond to tests that the IRR/Odds ratio for coefficient is equal to 1 (0 for OLS coefficients), and to a test that the IRR (or coefficients/Odds ratios) for both groups is different, in order to test for heterogeneous effects. IRRs are estimated by fitting a negative binomial regression model (with units reweighted following Hainmueller's entropy-based reweighting to account for imbalances due to attrition for follow up outcomes) (1). CI = 95% confidence interval. Odds ratio are estimated by fitting a logistic regression. Coefficients for WTP Masks and Donation outcomes are obtained by fitting an OLS regression. WTP = Willingness To Pay.

Supplement Table 2e. Analyses of Outcomes by Party*

	**Control**			**Intervention**		
Outcome	**Rep IR**	**Dem IR**	**Indep IR**	**Rep IR**	**Dem IR**	**Indep IR**
Knowledge gap score	0.179 (0.169,0.188)	0.142 (0.133,0.152)	0.167 (0.157,0.177)	0.138 (0.134,0.142)	0.122 (0.117,0.127)	0.130 (0.126,0.135)
Information seeking behavior	0.236 (0.211,0.260)	0.326 (0.294,0.358)	0.238 (0.213,0.262)	0.245 (0.233,0.258)	0.331 (0.316,0.347)	0.259 (0.246,0.272)
Safety gap score	0.512 (0.472,0.551)	0.424 (0.385,0.463)	0.562 (0.519,0.605)	0.546 (0.528,0.565)	0.395 (0.374,0.415)	0.504 (0.483,0.526)
Knowledge gap follow up	0.249 (0.237,0.261)	0.228 (0.216,0.240)	0.245 (0.232,0.259)	0.240 (0.234,0.246)	0.222 (0.216,0.228)	0.230 (0.223,0.236)
Outcome	**Rep mean**	**Dem mean**	**Indep mean**	**Rep mean**	**Dem mean**	**Indep mean**

© 2021 Torres C et al. *JAMA Network Open.*

**WTP Masks**	12.126 (11.499,12.753)	13.554 (12.841,14.266)	12.584 (11.896,13.272)	12.898 (12.570,13.226)	14.435 (14.069,14.801)	12.680 (12.339,13.021)
**Donation to COVID Charity**	486.828 (466.702,506.953)	571.367 (552.549,590.184)	503.071 (484.037,522.105)	497.107 (486.913,507.301)	592.532 (582.595,602.470)	525.374 (516.012,534.735)
**Donation to Black Charity**	342.487 (324.009,360.964)	449.686 (431.479,467.894)	403.572 (385.690,421.454)	353.883 (344.444,363.322)	460.742 (451.862,469.621)	410.529 (401.908,419.150)
Outcome	**Rep Odds**	**Dem Odds**	**Indep Odds**	**Rep Odds**	**Dem Odds**	**Indep Odds**
**Trust in Federal response**	0.430 (0.392,0.467)	0.117 (0.089,0.146)	0.237 (0.204,0.270)	0.437 (0.418,0.456)	0.104 (0.091,0.117)	0.256 (0.239,0.273)
**Trust in Local response**	0.402 (0.365,0.439)	0.421 (0.377,0.465)	0.388 (0.350,0.426)	0.434 (0.415,0.453)	0.406 (0.384,0.427)	0.410 (0.390,0.429)

	**Rep*Intervention**		**Dem*Intervention**		**Indep*Intervention**		**Difference**			
Outcome	**IRR (CI 95%)**	**p-value**	**IRR (CI 95%)**	**p-value**	**IRR (CI 95%)**	**p-value**	**p-value dem = rep**	**p-value rep = indep**	**p-value dem = indep**	**Observations**
Knowledge gap score	0.772 (0.722,0.826)	<0.001	0.860 (0.788,0.938)	0.001	0.782 (0.728,0.840)	<0.001	0.056	0.82	0.10	9317
Information seeking behavior	1.032 (0.911,1.170)	0.62	1.016 (0.884,1.167)	0.82	1.095 (0.962,1.246)	0.17	0.869	0.52	0.44	9055
Safety gap score	1.042 (0.948,1.145)	0.39	0.933 (0.823,1.058)	0.28	0.903 (0.823,0.991)	0.03	0.168	0.03	0.68	3936
Knowledge gap follow up	0.948 (0.865,1.040)	0.26	0.976 (0.871,1.094)	0.68	0.921 (0.839,1.012)	0.09	0.695	0.67	0.44	3933
Outcome	**Coefficient (CI 95%)**	**p-value**	**Coefficient (CI 95%)**	**p-value**	**Coefficient (CI 95%)**	**p-value**	**p-value dem = rep**	**p-value rep = indep**	**p-value dem = indep**	**Observations**
**WTP Masks**	0.764 (0.045,1.483)	0.04	0.854 (0.012,1.696)	0.05	0.097 (-0.649,0.842)	0.80	0.874	0.21	0.19	9034
**Donation to COVID Charity**	9.973 (-10.759,30.704)	0.35	20.445 (-3.817,44.707)	0.10	22.466 (1.033,43.898)	0.04	0.520	0.41	0.90	9007
**Donation to Black Charity**	11.641 (-7.489,30.772)	0.23	11.635 (-10.760,34.030)	0.31	6.674 (-13.154,26.501)	0.51	1.000	0.72	0.75	9002
Outcome	**Odds ratio (CI 95%)**	**p-value**	**Odds ratio (CI 95%)**	**p-value**	**Odds ratio (CI 95%)**	**p-value**	**p-value dem = rep**	**p-value rep = indep**	**p-value dem = indep**	**Observations**
**Trust in Federal response**	1.033 (0.871,1.224)	0.71	0.871 (0.639,1.186)	0.38	1.110 (0.905,1.361)	0.32	0.34	0.59	0.20	9051
**Trust in Local response**	1.140 (0.960,1.353)	0.14	0.926 (0.757,1.132)	0.45	1.102 (0.921,1.319)	0.29	0.12	0.79	0.21	9051

© 2021 Torres C et al. *JAMA Network Open.*

*This table presents incidence rates (or means/Odds) and incidence rate ratios (or coefficients/Odds ratios) from the intervention for Whites, disaggregated by political category (Republican, Democrat or Independent). P-values correspond to tests that the IRR/Odds ratio for Republicans, Democrats and Independent is equal to 1 (0 for OLS coefficients), and to a test that the IRR (or coefficients/Odds ratios) are different between two out of the three political categories, in order to test for heterogeneous effects. IRRs are estimated by fitting a negative binomial regression model (with units reweighted following Hainmueller's entropy-based reweighting to account for imbalances due to attrition for follow up outcomes) (1). Odds ratio are estimated by fitting a logistic regression. Coefficients for WTP Masks and Donation outcomes are obtained by fitting an OLS regression. CI = 95% confidence interval. WTP = Willingness To Pay.

Supplement Table 2f. Analyses of Outcomes by Age*

	**Control**		**Intervention**		**Young*Intervention**		**Old*Intervention**		**Difference**	
Outcome, Panel = All	**Young IR**	**Old IR**	**Young IR**	**Old IR**	**IRR (CI 95%)**	**p-value**	**IRR (CI 95%)**	**p-value**	**p-value**	**Observations**
Knowledge gap score	0.224 (0.217,0.231)	0.263 (0.253,0.272)	0.195 (0.191,0.199)	0.239 (0.234,0.244)	0.872 (0.842,0.902)	<0.001	0.909 (0.878,0.942)	<0.001	0.09	18762
Information seeking behavior	0.321 (0.306,0.337)	0.319 (0.301,0.337)	0.347 (0.339,0.355)	0.327 (0.318,0.336)	1.080 (1.014,1.149)	0.02	1.020 (0.950,1.095)	0.59	0.24	18223
Safety gap score	0.494 (0.466,0.522)	0.431 (0.404,0.458)	0.469 (0.455,0.483)	0.429 (0.415,0.442)	0.945 (0.892,1.001)	0.05	0.985 (0.909,1.068)	0.72	0.41	6035
Knowledge gap follow up	0.265 (0.255,0.274)	0.232 (0.225,0.240)	0.254 (0.249,0.259)	0.226 (0.222,0.229)	0.953 (0.905,1.003)	0.07	0.959 (0.893,1.030)	0.25	0.89	6030
Outcome, Panel = All	**Young mean**	**Old mean**	**Young mean**	**Old mean**	**Coefficient (CI 95%)**	**p-value**	**Coefficient (CI 95%)**	**p-value**	**p-value**	**Observations**
**WTP Masks**	13.477 (13.073,13.882)	15.004 (14.526,15.481)	13.978 (13.770,14.187)	15.494 (15.240,15.748)	0.500 (0.050,0.951)	0.03	0.471 (-0.087,1.029)	0.10	0.94	16759
**Donation to COVID Charity**	511.884 (501.990,521.778)	528.023 (514.622,541.424)	526.859 (521.763,531.956)	555.635 (548.882,562.387)	14.948 (3.387,26.508)	0.01	27.159 (12.775,41.542)	<0.001	0.20	16634
**Donation to Black Charity**	453.523 (443.986,463.059)	410.381 (397.634,423.128)	464.437 (459.562,469.313)	429.087 (422.696,435.477)	10.923 (0.112,21.733)	0.05	18.152 (4.786,31.517)	0.008	0.41	16783
Outcome, Panel = All	**Young Odds**	**Old Odds**	**Young Odds**	**Old Odds**	**Odds ratio (CI 95%)**	**p-value**	**Odds ratio (CI 95%)**	**p-value**	**p-value**	**Observations**
**Trust in Federal response**	0.233 (0.215,0.251)	0.179 (0.160,0.198)	0.229 (0.220,0.238)	0.192 (0.182,0.201)	0.974 (0.866,1.094)	0.66	1.119 (0.955,1.312)	0.17	0.17	17994
**Trust in Local response**	0.343 (0.323,0.364)	0.324 (0.301,0.348)	0.349 (0.338,0.359)	0.327 (0.315,0.339)	1.023 (0.924,1.133)	0.66	1.012 (0.893,1.146)	0.86	0.89	18132
Outcome, Panel = Black	**Young IR**	**Old IR**	**Young IR**	**Old IR**	**IRR (CI 95%)**	**p-value**	**IRR (CI 95%)**	**p-value**	**p-value**	**Observations**
Knowledge gap score	0.256 (0.246,0.265)	0.415 (0.401,0.430)	0.235 (0.230,0.240)	0.398 (0.390,0.406)	0.918 (0.879,0.959)	<0.001	0.958 (0.917,1.002)	0.06	0.18	9445
Information seeking behavior	0.373 (0.352,0.394)	0.388 (0.358,0.418)	0.398 (0.387,0.409)	0.404 (0.389,0.420)	1.068 (0.990,1.151)	0.09	1.042 (0.947,1.146)	0.40	0.69	9168

© 2021 Torres C et al. *JAMA Network Open.*

Safety gap score	0.412 (0.372,0.452)	0.342 (0.280,0.404)	0.405 (0.385,0.426)	0.301 (0.272,0.331)	0.968 (0.879,1.067)	0.52	0.873 (0.709,1.074)	0.20	0.37	2099
Knowledge gap follow up	0.269 (0.257,0.282)	0.252 (0.228,0.275)	0.266 (0.259,0.272)	0.235 (0.227,0.242)	0.977 (0.912,1.045)	0.49	0.928 (0.808,1.067)	0.30	0.52	2097
Outcome, Panel = Black	**Young mean**	**Old mean**	**Young mean**	**Old mean**	**Coefficient (CI 95%)**	**p-value**	**Coefficient (CI 95%)**	**p-value**	**p-value**	**Observations**
**WTP Masks**	14.316 (13.761,14.871)	19.354 (18.486,20.223)	15.049 (14.752,15.347)	18.917 (18.435,19.399)	0.731 (0.076,1.387)	0.03	-0.437 (-1.495,0.621)	0.42	0.07	7725
**Donation to COVID Charity**	518.233 (505.557,530.910)	530.836 (508.624,553.048)	541.320 (534.797,547.844)	548.816 (537.254,560.378)	23.119 (8.336,37.902)	0.002	17.971 (-6.205,42.147)	0.15	0.72	7627
**Donation to Black Charity**	486.301 (474.485,498.118)	486.184 (464.817,507.550)	504.653 (498.490,510.816)	503.550 (492.638,514.462)	18.373 (4.250,32.496)	0.01	17.367 (-5.141,39.876)	0.13	0.94	7781
Outcome, Panel = Black	**Young Odds**	**Old Odds**	**Young Odds**	**Old Odds**	**Odds ratio (CI 95%)**	**p-value**	**Odds ratio (CI 95%)**	**p-value**	**p-value**	**Observations**
**Trust in Federal response**	0.207 (0.183,0.230)	0.030 (0.017,0.043)	0.202 (0.190,0.213)	0.046 (0.038,0.054)	0.964 (0.820,1.134)	0.66	1.552 (0.959,2.511)	0.07	0.07	8943
**Trust in Local response**	0.336 (0.309,0.363)	0.156 (0.129,0.183)	0.334 (0.320,0.348)	0.142 (0.129,0.155)	0.990 (0.862,1.137)	0.89	0.894 (0.708,1.128)	0.35	0.46	9081
Outcome, Panel = White	**Young IR**	**Old IR**	**Young IR**	**Old IR**	**IRR (CI 95%)**	**p-value**	**IRR (CI 95%)**	**p-value**	**p-value**	**Observations**
Knowledge gap score	0.184 (0.175,0.193)	0.145 (0.139,0.152)	0.145 (0.141,0.149)	0.116 (0.113,0.119)	0.791 (0.747,0.837)	<0.001	0.802 (0.752,0.856)	<0.001	0.74	9317
Information seeking behavior	0.257 (0.235,0.280)	0.265 (0.244,0.286)	0.282 (0.271,0.294)	0.267 (0.256,0.277)	1.097 (0.986,1.220)	0.09	1.000 (0.898,1.114)	>0.99	0.23	9055
Safety gap score	0.572 (0.534,0.610)	0.450 (0.421,0.480)	0.531 (0.512,0.550)	0.458 (0.443,0.473)	0.922 (0.853,0.997)	0.04	1.015 (0.929,1.108)	0.74	0.11	3936
Knowledge gap follow up	0.260 (0.247,0.274)	0.228 (0.221,0.236)	0.243 (0.236,0.249)	0.223 (0.219,0.228)	0.922 (0.853,0.997)	0.04	0.970 (0.893,1.054)	0.47	0.39	3933
Outcome, Panel = White	**Young mean**	**Old mean**	**Young mean**	**Old mean**	**Coefficient (CI 95%)**	**p-value**	**Coefficient (CI 95%)**	**p-value**	**p-value**	**Observations**
**WTP Masks**	12.450 (11.867,13.033)	12.918 (12.401,13.436)	12.668 (12.386,12.950)	13.849 (13.568,14.129)	0.218 (-0.404,0.840)	0.49	0.907 (0.278,1.535)	0.005	0.13	9034
**Donation to COVID Charity**	504.094 (488.495,519.694)	526.700 (509.986,543.414)	509.135 (501.125,517.145)	558.767 (550.467,567.066)	4.924 (-13.036,22.884)	0.59	31.460 (13.315,49.605)	0.001	0.04	9007
**Donation to Black Charity**	413.284 (398.161,428.408)	372.778 (357.510,388.047)	415.170 (407.685,422.655)	392.169 (384.574,399.764)	1.781 (-14.761,18.323)	0.83	18.541 (1.778,35.303)	0.03	0.16	9002
Outcome, Panel = White	**Young Odds**	**Old Odds**	**Young Odds**	**Old Odds**	**Odds ratio (CI 95%)**	**p-value**	**Odds ratio (CI 95%)**	**p-value**	**p-value**	**Observations**
**Trust in Federal response**	0.266 (0.237,0.294)	0.288 (0.259,0.318)	0.262 (0.248,0.277)	0.299 (0.284,0.314)	0.984 (0.832,1.164)	0.85	1.069 (0.902,1.267)	0.44	0.50	9051
**Trust in Local response**	0.353 (0.322,0.384)	0.453 (0.421,0.486)	0.367 (0.351,0.382)	0.468 (0.452,0.485)	1.064 (0.914,1.238)	0.42	1.061 (0.916,1.229)	0.43	0.98	9051

*This table presents incidence rates (or means/Odds) and incidence rate ratios (or coefficients/Odds ratios) from the intervention, disaggregated by age category (<45 years old or >= 45 years old). P-values correspond to tests that the IRR/Odds ratio is equal to 1 (0 for OLS coefficients), and to a test that the IRR (or

© 2021 Torres C et al. *JAMA Network Open.*

coefficients/Odds ratios) are different between two out of two age categories, in order to test for heterogeneous effects. IRRs are estimated by fitting a negative binomial regression model (with units reweighted following Hainmueller's entropy-based reweighting to account for imbalances due to attrition for follow up outcomes) (1). Odds ratio are estimated by fitting a logistic regression. Coefficients for WTP Masks and Donation outcomes are obtained by fitting an OLS regression. CI = 95% confidence interval. WTP = Willingness To Pay.

Supplement Table 2g. Analyses of Outcomes by Household Income*

	**Control**		**Intervention**		**Low income*Intervention**		**High income*Intervention**		**Difference**	
Outcome	**Low income IR**	**High income IR**	**Low income IR**	**High income IR**	**IRR (CI 95%)**	**p-value**	**IRR (CI 95%)**	**p-value**	**p-value**	**Observations**
Knowledge gap score	0.193 (0.187,0.198)	0.228 (0.215,0.241)	0.160 (0.157,0.162)	0.206 (0.199,0.213)	0.830 (0.802,0.859)	<0.001	0.903 (0.855,0.954)	<0.001	0.01	16135
Information seeking behavior	0.307 (0.292,0.321)	0.331 (0.306,0.356)	0.322 (0.314,0.329)	0.361 (0.348,0.374)	1.044 (0.986,1.105)	0.14	1.095 (0.992,1.208)	0.07	0.41	15611
Safety gap score	0.454 (0.432,0.476)	0.507 (0.465,0.549)	0.446 (0.435,0.457)	0.466 (0.445,0.487)	0.974 (0.923,1.029)	0.35	0.914 (0.833,1.002)	0.06	0.24	6035
Knowledge gap follow up	0.250 (0.243,0.257)	0.247 (0.233,0.262)	0.244 (0.240,0.247)	0.229 (0.222,0.235)	0.968 (0.923,1.015)	0.18	0.916 (0.841,0.997)	0.04	0.27	6030
Outcome	**Low income mean**	**High income mean**	**Low income mean**	**High income mean**	**Coefficient (CI 95%)**	**p-value**	**Coefficient (CI 95%)**	**p-value**	**p-value**	**Observations**
**WTP Masks**	12.892 (12.546,13.238)	15.357 (14.673,16.042)	13.386 (13.204,13.568)	16.030 (15.677,16.383)	0.478 (0.067,0.888)	0.02	0.682 (-0.039,1.403)	0.06	0.63	15471
**Donation to COVID Charity**	524.869 (515.534,534.203)	507.280 (489.786,524.774)	541.503 (536.743,546.262)	537.629 (528.634,546.624)	16.134 (5.453,26.815)	0.003	30.646 (11.832,49.459)	0.001	0.19	15432
**Donation to Black Charity**	438.473 (429.519,447.427)	415.834 (399.082,432.586)	448.682 (444.131,453.234)	440.535 (432.067,449.004)	9.879 (-0.063,19.821)	0.05	25.087 (7.607,42.567)	0.005	0.14	15435
Outcome	**Low income Odds**	**High income Odds**	**Low income Odds**	**High income Odds**	**Odds ratio (CI 95%)**	**p-value**	**Odds ratio (CI 95%)**	**p-value**	**p-value**	**Observations**
**Trust in Federal response**	0.223 (0.207,0.240)	0.275 (0.244,0.307)	0.229 (0.221,0.238)	0.262 (0.246,0.277)	1.047 (0.936,1.171)	0.42	0.914 (0.760,1.100)	0.34	0.22	15580
**Trust in Local response**	0.376 (0.356,0.395)	0.381 (0.346,0.415)	0.382 (0.372,0.392)	0.384 (0.367,0.401)	1.023 (0.931,1.124)	0.64	1.021 (0.867,1.202)	0.81	0.98	15593

*This table presents incidence rates (or means/Odds) and incidence rate ratios (or coefficients/Odds ratios) from the intervention, disaggregated by household income category (<60k or >= 60k). P-values correspond to tests that the IRR/Odds ratio is equal to 1 (0 for OLS coefficients), and to a test that the IRR (or coefficients/Odds ratios) are different between two out of two income categories, in order to test for heterogeneous effects. IRRs are estimated by fitting a negative binomial regression model (with units reweighted following Hainmueller's entropy-based reweighting to account for imbalances due to attrition for follow up outcomes) (1). Odds ratio are estimated by fitting a logistic regression. Coefficients for WTP Masks and Donation outcomes are obtained by fitting an OLS regression. CI = 95% confidence interval. WTP = Willingness To Pay.

© 2021 Torres C et al. *JAMA Network Open.*

eTable 3. Effects of Any Message Intervention: Effects on Additional Outcomes*

		Mean Odds				Odds ratio (CI 95%)	p-value	Observations
Panel A	Outcome	**Control**	**Nb obs**	**Intervention**	**Nb obs**			
All	**Trust in Federal response**	0.210 (0.196,0.223)	3612	0.213 (0.206,0.219)	14382	1.024 (0.932,1.125)	0.62	17994
	**Trust in Local response**	0.335 (0.320,0.350)	3639	0.339 (0.332,0.347)	14493	1.020 (0.942,1.104)	0.63	18132
Black	**Trust in Federal response**	0.142 (0.126,0.158)	1798	0.144 (0.136,0.152)	7145	1.021 (0.876,1.189)	0.79	8943
	**Trust in Local response**	0.268 (0.248,0.289)	1827	0.262 (0.251,0.272)	7254	0.965 (0.857,1.087)	0.56	9081
White	**Trust in Federal response**	0.277 (0.256,0.297)	1814	0.280 (0.270,0.291)	7237	1.026 (0.911,1.157)	0.67	9051
	**Trust in Local response**	0.402 (0.380,0.425)	1812	0.418 (0.406,0.429)	7239	1.064 (0.958,1.183)	0.25	9051
Panel B	Outcome	**Mean**				**coefficient (CI 95%)**	**p-value**	**Observations**
All	**Donation to COVID Charity**	518.449 (510.457,526.441)	3346	538.190 (534.109,542.270)	13288	19.501 (10.493,28.509)	<0.001	16634
	**Donation to Black Charity**	436.260 (428.569,443.951)	3368	450.343 (446.448,454.237)	13415	14.004 (5.601,22.406)	0.001	16783
Black	**Donation to COVID Charity**	521.871 (510.840,532.902)	1540	543.363 (537.670,549.056)	6087	21.511 (8.904,34.118)	0.001	7627
	**Donation to Black Charity**	486.046 (475.641,496.451)	1564	504.359 (498.965,509.754)	6217	18.346 (6.389,30.304)	0.003	7781
White	**Donation to COVID Charity**	515.532 (504.095,526.968)	1806	533.816 (528.027,539.605)	7201	17.788 (5.025,30.551)	0.006	9007
	**Donation to Black Charity**	393.098 (382.313,403.882)	1804	403.688 (398.353,409.024)	7198	10.266 (-1.504,22.037)	0.09	9002

* This table presents mean values of secondary outcomes in Control and in Any Message Intervention groups, and the effect estimated by ordinary least squares regressions for Donations, and by logistic regression for Trust outcomes. Nb obs = number of observations.

© 2021 Torres C et al. *JAMA Network Open.*

eTable 4. Effects of Tailoring Messages on Additional Outcomes

Supplement Table 4a. Effects of Tailoring Messages on Additional Outcomes*

		Black Physician * Intervention		AMA anti-racism* Intervention		Doctor racial disc. * Intervention		Black physician		AMA anti-racism		Covid treatment		
Panel A	Outcome	**Odds ratio (CI 95%)**	**p-value**	**Odds ratio (CI 95%)**	**p-value**	**Odds ratio (CI 95%)**	**p-value**	**Odds ratio (CI 95%)**	**p-value**	**Odds ratio (CI 95%)**	**p-value**	**Odds ratio (CI 95%)**	**p-value**	**Observations**
All	**Trust in Federal response**	1.135 (0.940,1.370)	0.19	1.014 (0.840,1.225)	0.88	0.986 (0.906,1.072)	0.74	0.853 (0.721,1.010)	0.07	0.969 (0.818,1.147)	0.712	0.962 (0.814,1.137)	0.653	17994
	**Trust in Local response**	0.942 (0.804,1.104)	0.46	0.864 (0.738,1.013)	0.07	0.909 (0.847,0.976)	0.008	1.034 (0.897,1.191)	0.65	1.184 (1.027,1.364)	0.020	1.186 (1.028,1.369)	0.019	18132
Black	**Trust in Federal response**	0.983 (0.724,1.334)	0.91	0.924 (0.680,1.254)	0.61	1.074 (0.937,1.232)	0.30	0.922 (0.701,1.212)	0.56	1.072 (0.815,1.410)	0.618	1.034 (0.786,1.359)	0.813	8943
	**Trust in Local response**	0.952 (0.750,1.208)	0.68	0.857 (0.675,1.088)	0.21	0.963 (0.864,1.072)	0.49	1.030 (0.833,1.275)	0.78	1.265 (1.022,1.566)	0.031	1.093 (0.880,1.359)	0.421	9081
White	**Trust in Federal response**	1.241 (0.977,1.577)	0.08	1.075 (0.846,1.366)	0.55	0.935 (0.840,1.040)	0.22	0.813 (0.656,1.008)	0.06	0.910 (0.734,1.127)	0.386	0.921 (0.745,1.137)	0.444	9051
	**Trust in Local response**	0.934 (0.756,1.154)	0.53	0.876 (0.709,1.082)	0.22	0.870 (0.792,0.956)	0.004	1.038 (0.859,1.254)	0.70	1.118 (0.926,1.351)	0.246	1.261 (1.042,1.524)	0.017	9051
Panel B	Outcome	**Coefficient (CI 95%)**	**p-value**	**Coefficient (CI 95%)**	**p-value**	**Coefficient (CI 95%)**	**p-value**	**Coefficient (CI 95%)**	**p-value**	**Coefficient (CI 95%)**	**p-value**	**Coefficient (CI 95%)**	**p-value**	**Observations**
All	**Donation to COVID Charity**	0.385 (-17.630,18.401)	0.97	15.878 (-2.137,33.893)	0.08	4.549 (-3.532,12.630)	0.27	2.360 (-13.742,18.462)	0.77	-11.221 (-27.322,4.881)	0.172	9.109 (-7.026,25.245)	0.269	16634
	**Donation to Black Charity**	-12.444 (-29.244,4.357)	0.15	-5.773 (-22.574,11.028)	0.50	9.027 (1.500,16.555)	0.02	15.757 (0.736,30.778)	0.04	7.157 (-7.863,22.178)	0.350	18.723 (3.644,33.802)	0.015	16783
Black	**Donation to COVID Charity**	-8.567 (-33.776,16.641)	0.51	32.702 (7.493,57.912)	0.01	9.231 (-2.098,20.559)	0.11	7.163 (-15.358,29.683)	0.53	-25.387 (-47.908,-2.865)	0.027	4.679 (-17.858,27.217)	0.684	7627
	**Donation to Black Charity**	-20.863 (-44.770,3.044)	0.09	-14.436 (-38.347,9.476)	0.24	12.748 (2.027,23.469)	0.02	20.518 (-0.852,41.888)	0.06	13.120 (-8.254,34.493)	0.229	29.872 (8.393,51.350)	0.006	7781

© 2021 Torres C et al. *JAMA Network Open.*

White	**Donation to COVID Charity**	7.870 (-17.662,33.401)	0.55	1.584 (-23.947,27.115)	0.90	0.563 (-10.870,11.997)	0.92	-1.546 (-24.375,21.284)	0.89	0.827 (-22.002,23.655)	0.943	12.784 (-10.116,35.685)	0.274	9007
	**Donation to Black Charity**	-4.931 (-28.474,18.612)	0.68	1.437 (-22.106,24.979)	0.91	5.724 (-4.816,16.264)	0.29	11.359 (-9.693,32.412)	0.29	2.256 (-18.796,23.307)	0.834	9.237 (-11.874,30.349)	0.391	9002

* This table presents estimated effects on secondary outcomes from specific tailorings of the video messages, within the Intervention Group. Effects are estimated by ordinary least squares regressions for Donation outcomes, and by logistic regression for Trust outcomes. CI = 95% confidence interval.

Supplement Table 4b. Effects of Tailoring Messages on Additional Outcomes: Average Incidence Rates*

			**Black Physician**		**AMA anti-racism**		**Doctor racial disc. in COVID-19**	
Panel	Outcome		**No**	**Yes**	**No**	**Yes**	**No**	**Yes**
All	**Trust in Federal response**	**Mean Odds**	0.216 (0.206,0.225)	0.210 (0.200,0.219)	0.213 (0.204,0.223)	0.212 (0.203,0.221)	0.213 (0.204,0.223)	0.212 (0.203,0.222)
		**Observations**	7192	7190	7180	7202	7228	7154
	**Trust in Local response**	**Mean Odds**	0.338 (0.327,0.349)	0.341 (0.330,0.352)	0.337 (0.326,0.348)	0.342 (0.331,0.353)	0.349 (0.338,0.360)	0.330 (0.319,0.341)
		**Observations**	7246	7247	7237	7256	7285	7208
Black	**Trust in Federal response**	**Mean Odds**	0.150 (0.138,0.162)	0.138 (0.127,0.150)	0.144 (0.133,0.156)	0.144 (0.133,0.156)	0.140 (0.129,0.152)	0.148 (0.136,0.160)
		**Observations**	3599	3546	3574	3571	3602	3543
	**Trust in Local response**	**Mean Odds**	0.263 (0.249,0.278)	0.260 (0.245,0.274)	0.254 (0.239,0.268)	0.270 (0.255,0.284)	0.265 (0.251,0.279)	0.258 (0.243,0.272)
		**Observations**	3652	3602	3629	3625	3658	3596
White	**Trust in Federal response**	**Mean Odds**	0.281 (0.267,0.296)	0.279 (0.265,0.294)	0.282 (0.267,0.297)	0.279 (0.264,0.293)	0.285 (0.271,0.300)	0.275 (0.261,0.290)
		**Observations**	3593	3644	3606	3631	3626	3611
	**Trust in Local response**	**Mean Odds**	0.413 (0.397,0.430)	0.422 (0.406,0.438)	0.420 (0.404,0.437)	0.415 (0.399,0.431)	0.434 (0.418,0.450)	0.401 (0.385,0.417)
		**Observations**	3594	3645	3608	3631	3627	3612
All	**Donation to COVID Charity**	**Mean**	540.199 (534.434,545.965)	536.184 (530.408,541.960)	535.902 (530.111,541.693)	540.464 (534.714,546.213)	536.060 (530.306,541.814)	540.351 (534.565,546.138)

© 2021 Torres C et al. *JAMA Network Open.*

		**Observations**	6637	6651	6624	6664	6694	6594
	**Donation to Black Charity**	**Mean**	452.611 (447.141,458.081)	448.074 (442.529,453.619)	449.496 (443.947,455.045)	451.181 (445.714,456.648)	446.475 (441.000,451.950)	454.276 (448.736,459.815)
		**Observations**	6709	6706	6673	6742	6763	6652
Black	**Donation to COVID Charity**	**Mean**	544.094 (536.175,552.013)	542.625 (534.439,550.810)	539.691 (531.773,547.609)	547.001 (538.821,555.180)	538.818 (530.851,546.785)	548.034 (539.898,556.170)
		**Observations**	3059	3028	3029	3058	3085	3002
	**Donation to Black Charity**	**Mean**	504.598 (496.942,512.254)	504.117 (496.515,511.719)	505.059 (497.483,512.635)	503.673 (495.992,511.353)	498.076 (490.520,505.631)	510.855 (503.156,518.555)
		**Observations**	3136	3081	3081	3136	3160	3057
White	**Donation to COVID Charity**	**Mean**	536.869 (528.592,545.147)	530.801 (522.704,538.898)	532.710 (524.382,541.037)	534.920 (526.874,542.965)	533.703 (525.485,541.921)	533.931 (525.774,542.087)
		**Observations**	3578	3623	3595	3606	3609	3592
	**Donation to Black Charity**	**Mean**	406.982 (399.529,414.436)	400.441 (392.808,408.074)	401.838 (394.171,409.505)	405.531 (398.108,412.955)	401.219 (393.666,408.772)	406.163 (398.625,413.702)
		**Observations**	3573	3625	3592	3606	3603	3595

*This table presents number of observations and average incidence rates for additional outcomes in the sample of participants who received intervention, split by whether they received a particular intervention or not. For instance, the first column shows the average incidence rate (and number of observations) for all participants that received the video message from a white physician, and the second column shows the average incidence rates (and number of observations) for all participants that received the video messages from a Black physician. 95% CI in parentheses.

© 2021 Torres C et al. *JAMA Network Open.*

eTable 5. Effects of All Black Treatments on Outcomes*

		Intervention * All Black treatments		Intervention		AMA anti-racism * Black Physician		
Panel	Outcome	IRR (CI 95%)	p-value	IRR (CI 95%)	p-value			Observations
All	Knowledge gap score	1.016 (0.973,1.060)	0.48	0.888 (0.866,0.911)	<0.001	0.988 (0.960,1.017)	0.41	18762
	Information seeking behavior	0.998 (0.923,1.079)	0.96	1.054 (1.005,1.106)	0.03	0.999 (0.947,1.053)	0.96	18223
	Safety gap score	1.028 (0.948,1.114)	0.51	0.956 (0.911,1.003)	0.07	0.939 (0.889,0.992)	0.03	6035
	Knowledge gap follow up	1.005 (0.935,1.079)	0.90	0.955 (0.915,0.997)	0.03	0.968 (0.922,1.016)	0.18	6030
Black	Knowledge gap score	1.014 (0.961,1.069)	0.61	0.936 (0.907,0.966)	<0.001	0.992 (0.957,1.028)	0.67	9445
	Information seeking behavior	0.992 (0.899,1.095)	0.88	1.059 (0.997,1.125)	0.06	1.008 (0.943,1.078)	0.82	9168
	Safety gap score	1.118 (0.960,1.302)	0.15	0.938 (0.858,1.026)	0.16	0.828 (0.746,0.919)	<0.001	2099
	Knowledge gap follow up	1.015 (0.915,1.127)	0.77	0.965 (0.907,1.027)	0.26	0.952 (0.887,1.021)	0.17	2097
White	Knowledge gap score	1.020 (0.944,1.102)	0.62	0.794 (0.760,0.830)	<0.001	0.978 (0.929,1.030)	0.40	9317
	Information seeking behavior	1.005 (0.886,1.139)	0.94	1.048 (0.970,1.132)	0.24	0.987 (0.906,1.076)	0.77	9055
	Safety gap score	0.974 (0.881,1.076)	0.60	0.966 (0.910,1.025)	0.25	1.014 (0.948,1.085)	0.69	3936
	Knowledge gap follow up	0.995 (0.901,1.098)	0.92	0.946 (0.893,1.003)	0.06	0.982 (0.918,1.050)	0.59	3933
	Outcome	coefficient (CI 95%)	p-value	coefficient (CI 95%)	p-value			Observations
All	**WTP Masks**	-0.073 (-0.659,0.514)	0.81	0.507 (0.149,0.865)	0.006	0.217 (-0.182,0.615)	0.29	16759
	**Donation to Black Charity**	23.764 (9.688,37.839)	0.001	11.078 (2.500,19.656)	0.01	-3.543 (-13.082,5.996)	0.47	16783
Black	**WTP Masks**	0.249 (-0.687,1.184)	0.60	0.388 (-0.181,0.957)	0.18	0.167 (-0.462,0.796)	0.60	7725
	**Donation to Black Charity**	15.716 (-4.386,35.817)	0.13	16.463 (4.257,28.669)	0.008	0.200 (-13.267,13.668)	0.98	7781
White	**WTP Masks**	-0.343 (-1.081,0.395)	0.36	0.609 (0.158,1.061)	0.008	0.260 (-0.246,0.765)	0.31	9034
	**Donation to Black Charity**	30.598 (10.930,50.266)	0.002	6.437 (-5.583,18.456)	0.29	-6.946 (-20.405,6.513)	0.31	9002

© 2021 Torres C et al. *JAMA Network Open.*

* This table presents estimated effects on primary outcomes from All Black treatments (Black physician, AMA anti-racism and Doctor racial discrepancy in COVID-19), interacted with Intervention. IRRs for follow up outcomes are estimated by fitting a negative binomial regression model (with units reweighted following Hainmueller's entropy-based reweighting to account for imbalances due to attrition (1) for follow-up outcomes). The regression equations are detailed in Section E.3. CI = 95% confidence interval. WTP = Willingness To Pay.

© 2021 Torres C et al. *JAMA Network Open.*

eReferences

1. Hainmueller J. Entropy balancing for causal effects: A multivariate reweighting method to produce balanced samples in observational studies. Political analysis. 2012:25-46.
2. Chernozhukov V, Chetverikov D, Demirer M, Duflo E, Hansen C, Newey W, et al. Double/debiased machine learning for treatment and structural parameters. The Econometrics Journal. 2018;21(1):C1-C68.

© 2021 Torres C et al. *JAMA Network Open.*
