# Supplementary material for: Effect of Physician-Delivered COVID-19 Public Health Messages and Messages Acknowledging Racial Inequity on Black and White Adults’ Knowledge, Beliefs, and Practices Related to COVID-19: A Randomized Clinical Trial
Source: JAMA Netw Open. 2021 Jul 14;4(7):e2117115. doi: 10.1001/jamanetworkopen.2021.17115 (PMC8280971; doi:10.1001/jamanetworkopen.2021.17115)
Supplement: Supplement 2. — Pre-analysis Plan and Trial Protocol [file jamanetwopen-e2117115-s002.pdf]

(Round 2) Pre-Analysis Plan

COVID-19 Health Messaging to Underserved Communities

This Draft: September 4, 2020

1. Introduction

The aim of this study is to build on results from our first experiment and test how acknowledging institutional racial injustice affects informational messaging. In particular, we will investigate how well study participants retain knowledge and update beliefs and behavior with respect to COVID-19. We will also test the effect of race concordance of providers with recipients, and whether highlighting the unequal burden of the disease has additional effects on knowledge, beliefs and behavior regarding COVID-19.

Compared to the first study, there are four additional changes to the experimental design of note: 1) the sample will include white respondents in addition to African American respondents;[1] 2) the group that does not view videos about COVID-19 will watch a set of placebo videos about non-COVID health topics; 3) we are collecting follow-up behavioral data from a subset of participants;[2] 4) this study does not include a few of the treatment variants from the first – specifically, there is no acknowledgment by doctors in the videos discussing health behaviors, and there is no treatment arm to alter perceptions of whether mask-wearing is socially-acceptable.

2. Treatments, and Experimental Protocols

2.1 Treatments

Each subject receives one AMA statement and then watches three videos pertaining to health. The AMA statement either addresses racial injustice (treatment [RI]) or drug pricing (placebo [DP]). The videos pertaining to health behavior either discuss COVID-19 and prevention (treatment) or other non-COVID-19 related issues (control).

- AMA Statement:

  RI: Racial Injustice

  DP: Drug Pricing

- Treatment Videos

[1] We continue to focus our sample on individuals with less than college education.

[2] We are also collecting baseline measures of these behaviors.

1. Video T1:
    - Introduction
    - Discussion of symptoms
2. Video T2:
    - Information about social distancing and hygiene
    - Sub-treatment T2A:
        * Acknowledgment of racial disparities in contagion and mortality.
3. Video T3:
    - Information about masks

- Placebo Videos

1. Video C1:
    - Information about fitness routines
2. Video C2:
    - Information about sleep hygiene
3. Video C3:
    - Information about sugar intake

We are varying five aspects of the videos across treatments:

1. AMA statement regarding racial injustice or transparency in drug pricing.
2. Racial concordance with AMA spokespeople in the videos.
3. Racial concordance with partner doctors in the videos.
4. Informative videos about COVID-19 or non-COVID-19 issues.
5. Video T2 content:
    - Standard message about hygiene and social distancing
    - Previous message, plus acknowledgment of racial disparities in contagion and mortality.

All of the video scripts can be found in Appendix Section A. Figure 1 shows how these different treatments are incorporated in the randomization design.

2.2 Recruitment and Sampling

We are using Lucid to recruit a sample and to compensate subjects for their participation. Our total target sample size is 20,000 subjects, 10,000 African American respondents, and 10,000 White American respondents. We require all participants to be age 18 or older, and we are targeting individuals who have *not* completed a college degree.

Figure 1: Treatment Design

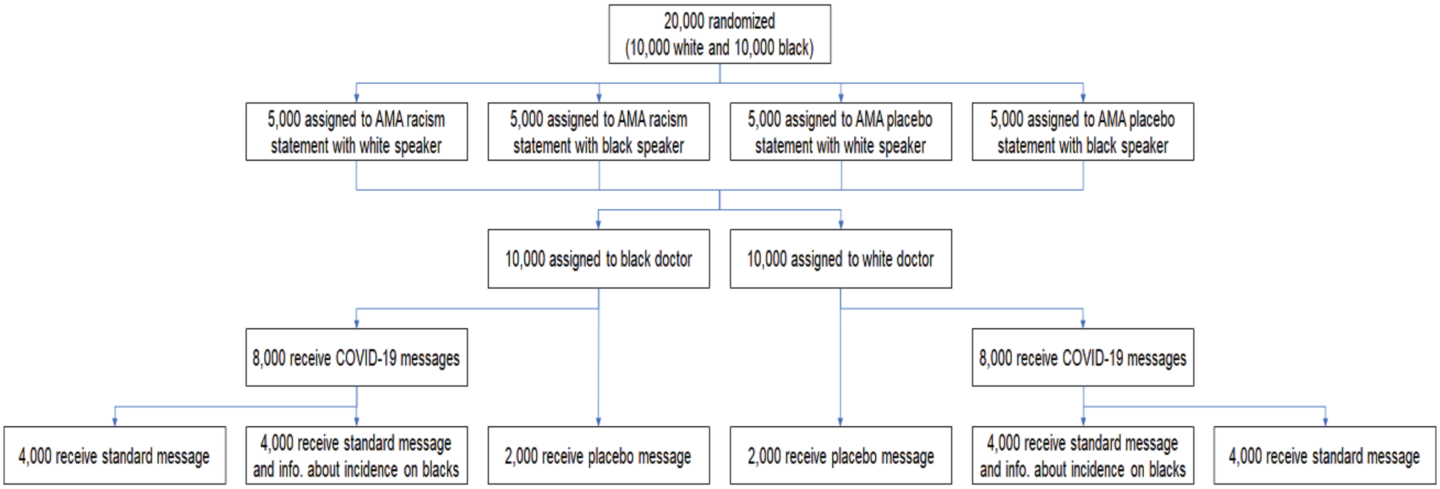

Note: There are 5 randomization instances summarized in this diagram. For the fully extended tree with all 24 final individual cells, see Figure 2.

2.3 Experimental Protocols and Treatments

Our experiment has the following structure:

1. Recruitment and Baseline survey
   (a) Recruit target sample via Lucid
   (b) Collect demographic information, political views, and mask ownership. Several demographics variables are being collected directly by Lucid.
2. Randomize subjects to treatment
   - Treatment scripts are tailored to each racial group
   - Within racial group, treatment is stratified on age, gender and geographic region.
   - Randomization is at the individual level according to Figure 1.
   - There will also be a control group who will not receive COVID-19 videos, but will receive information at the very end of the survey.
3. Video delivery for all individuals.
   (a) After each of the three videos, respondents evaluate the video's usefulness, and trustworthiness, along with the respondents intention to follow the advice in the video and share information from the video.
4. Endline survey
5. Debrief script

6. Follow-up survey
   (a) We will follow up with participants three days post-treatment.
   (b) The chief purpose would be to measure health-seeking and health-preserving behaviors.

3. General Hypotheses to be Tested

This section lists the general hypotheses we want to test. For those, we are planning to run broadly pooled treatments (and specific interactions specified below). Main hypotheses:

1. Baseline treatment effect: Does any COVID-19 message affect knowledge/indended behavior/priorities in donations/actual behavior?
   (a) Compare the effectiveness of any doctor treatment video vs. any doctor placebo video.
   - We will test this in the full sample and in the white and African American samples, separately.
2. Concordance effect: Does the concordance of the doctor in the COVID-19 video affect the impacts of the message?
   (a) Within the treatment group receiving a COVID-19 doctor video, compare the effectiveness of the video if the respondent is randomized to a doctor of concordant vs. discordant race.
   - We will test this hypothesis separately in the white and African American samples. If the effects go in the same direction for both populations, we will also test this hypothesis in the full sample.
   - An alternate way to define concorance is whether the subject views a racially concordant messenger both (or either) in the COVID-19 doctor video and in an AMA statement of any kind. We will also test the effectiveness of the messaging under this alternate measure.
3. AMA acknowledgement: Does the AMA message mediate the impacts of the COVID-19 messaging or of the racial concordance of the messenger?
   (a) Compare baseline treatment effect [1] for AMA acknowledgement vs AMA placebo.
   (b) Compare concordance effect [2] for AMA acknowledgment vs AMA placebo. Is the concordance of the doctor in the COVID-19 video more or less important when there is an AMA message vs. the AMA placebo?
   - We will test these hypotheses separately for white and African American respondents.
4. Does the racial concordance of the AMA messenger matter?
   (a) Compare whether AMA acknowledgement [3a] is more effective if the AMA messenger is racially concordant.

(b) Compare AMA acknowledgement effect [3b] by racial concordance of AMA messenger. Is the concordance of the doctor in the COVID-19 video more or less important when there is a racially concordant AMA message vs. a non-racially concordant AMA message?

- We will test these hypotheses separately for white and African American respondents.

5. Content of message: does the mention of the racial disparities (video 2RD) in disease burden affect knowledge/intended behavior/ priorities in donation/actual behavior?

   (a) Within the COVID video treatment group, compare videos with RD information vs. no RD information.

   (b) Within the COVID video treatment group, how does information about RD interact with the racial concordance of the doctor messenger? Does information about RD make racial concodance more important, especially among African American respondents?

   (c) Within the COVID video treatment group, how does information about RD interact with the AMA racial injustice message? Does information about RD make the AMA acknowledgement more effective, especially among African American respondents?

   (d) We are also interested in how information about RD mediates Hypotheses 3b, 4a, 4b, and 4c.

   - In addition to the standard outcomes, we are also particularly interested in impacts on donations to a COVID fund that is specifically for African Americans vs. a general COVID fund. We also are interested in how this information changes perceptions of the policy responses of state and federal governments.
   - For 5a, we are interested in the white and African American groups, separately, and also the full, pooled sample. For 5b and 5c, we are mainly interested in the effects separately for white and African American respondents.
   - RD should have the largest impacts for individuals who have incorrect beliefs. Moreover, the impacts of the information should be different for individuals with priors about relative impacts on African Americans that are too low versus too high. (See Section 5.4, below).

4. Data Collection and Outcomes

We will run our experiment beginning on August 7, 2020. We are recruiting individuals that fit our screening criteria from an online survey firm. The data will be collected from respondent surveys. There is a short baseline module before individuals are exposed to the videos. Following the final video, there is an endline outcome module that all participants complete. Respondents also evaluate each video immediately after watching it. We also plan to follow up with individuals a few days after the treatment delivery for an additional survey to measure whether the videos changed behaviors. Prior to treatment, we ask all participants whether they would be willing to answer the follow-up survey. We allow individuals to enroll in the study, independent of their follow-up availability. We plan to control for willingness to answer a follow-up in all regressions. All of the survey responses will be downloaded in a .csv file for cleaning and analysis in R.

4.1 Baseline Survey Variables

The baseline information for all respondents includes the following demographic characteristics:

- Education
- Age
- Location
- Sex
- Political Views

We elicit additional health information at baseline, but participants may choose not to answer these questions:

- Mask Ownership
- Public Excursions
- Health Behavior

See Section 5, below for a summary of our key endline survey variables.

4.2 Data quality checks

We have several questions in the survey that capture respondent attention. We use some of these questions to screen out low-attention survey takers before assigning treatment. We further plan to exclude those that take very little time on the survey overall and/or on the videos.

5. Empirical Analysis

5.1 Balance Checks

We will conduct a series of balance tests across treatment arms to ensure that there are no chance differences between subjects in the various arms. We will regress characteristics measured pre-treatment on indicators for the arms and test their individual and joint significance. Balance tests will be conducted using all of the variables measured in the baseline survey.
We will also test for balance in attrition rates (see Section 6.2, below).

5.2 Key Outcomes

We intend to measure the treatment effects on the following set of outcomes:

1. The number of participants with knowledge of COVID related symptoms and transmission as assessed by a questionnaire we've developed specifically relevant to the intervention videos we are using for the project. Specifically, we define the following three outcomes, measured both at first contact and followup:

- Knowledge of COVID symptoms: participants are asked to identify 4 symptoms of COVID from a list of 9. We will define an indicator for whether participants select the 3 most common symptoms (fever, cough, difficulty breathing).
- Knowledge of COVID prevention: participants are asked to identify 3 COVID prevention behaviors from a list of 7. We will define an indicator for whether participants select the 3 behaviors emphasized in the videos (staying outside and 6ft from others; washing hands when going and coming from home; mask wearing).
- Knowledge of asymptomatic infection: an indicator for whether participants correctly answer that COVID can spread asymptomatically.

2. Behavioral outcome 1: Number of participants who report behavior change related to messages provided in the intervention videos; measured via a specific questionnaire instrument we've developed to correspond to the intervention. Specific behaviors include physical distancing, mask wearing, and hand hygiene. Since we ask about several safety behaviors, we will define a standardized index to combine them into one measure, calculated at both first contact and endline. The key outcome is the within-person change in this safety index from first contact to endline.

3. Behavioral outcome 2: Revealed preference estimate of willingness to pay for masks. The subjects will trade-off the willingness to get a pair of masks or an amazon gift card when participating in a strategy-proof lottery.

4. Behavioral outcome 3: Number of links people click on for additional information on the COVID-19 behaviors. Links will include testing locations, state public health hotline, and symptom tracker.

5. Behavioral outcome 4: Donations to a COVID-related charity. After providing information on the number of weekly COVID cases, we are measuring the willingness to donate to a COVID-related charity vs. a generic helath-related charity.

6. Behavioral outcome 5: Donations to African-American COVID-19 fund. After providing information on the disproportionate burden on African-American communities, we are measuring the willingness to donate to a COVID relief fund that focuses on African-American communities vs. one that generically provides relief to disadvantaged individuals.

7. Evaluation of state and local COVID policy. We are measuring how how well participants think their federal and state governments managed to balance opening the economy and limiting the health impacts of Covid-19. We believe that information about racial disparities, in particular, may change these perceptions, especially for people who believed that racial disparities did not exist or were less severe at baseline.

5.3 Regression Analysis

We will perform different regression analyses to test the hypotheses listed above. Because our data contains many possible control variables, we will use a double-lasso procedure to select regression controls. We will also include a control for whether the participant's availability to participate in a

follow up survey (as indicted at baseline). These control variables are denoted as X in the regression specifications below.

Unless otherwise noted, we will examine treatment effects on knowledge of COVID symptoms, knowledge of COVID transmission, intended donation to a COVID-related charity, willingness to pay for masks, changes in reported behavior. In what follows, we present the minimal regressions to test each of the hypotheses, restricting to the smallest subset of treatments. However, we could also execute the same tests in the full sample, but with more treatment interactions.[3]

Note that the "COVID" indicator covers cases when doctors mention COVID plus racism, or COVID alone. In text, we refer to this group as the treatment group.

1. **Baseline treatment effect: does any of the COVID-19 messaging affect knowledge, intended behavior, priorities in donation, or actual behavior?**

   - Question: does COVID messaging from doctors have any effect?
     Samples: separate analysis for all respondents, black respondents, and white respondents.
     Regression:

$$Y = \beta \cdot \text{COVID Video} + X'\gamma + \epsilon$$

2. **Does racial concordance of the doctor in the COVID-19 video change the effectiveness for messaging?**

   - Question: Bolded, immediately above.
     Samples: COVID video groups. Separate analysis for black and white respondents.
     Regression:

$$Y = \beta \cdot \text{DocConcord} + X'\gamma + \epsilon$$

     Alternate Specification: To allow, for a level effect of doctor concordance, we can also run the following modified regression, including both the COVID video and control groups.

$$Y = \beta \cdot \text{DocConcord} \cdot \text{COVID Video} + \alpha \cdot \text{COVID Video} + \delta \cdot \text{DocConcord} + X'\gamma + \epsilon$$

   - Question: Bolded above, but using an alternate definition of concordance. We'll require a) all messengers or b) any messenger to concord with the respondent's race.

   For any outcomes where β is of the same sign for white and black respondents, we will estimate the above equation again in the full sample.

3. **Does the AMA racism acknowledgment affect the impact of messaging on any outcomes? Does it change the concordance effect?**

[3] In some cases, especially when including controls, these full-sample tests may be preferrable to these stripped-down regressions.

- Question: Does the AMA racism messaging heighten or dull COVID messaging from doctors?
  Samples: COVID video groups. Separate analysis for all respondents, black respondents, and white respondents.
  Regression:

  $$Y = \beta \cdot \text{AMARacism} + X'\gamma + \epsilon$$

  Alternate Specification: To allow, for a level effect of the AMA racism message, we can also run the following modified regression, including both the COVID video and control groups.[4]

  $$Y = \delta \cdot \text{AMARacism} \cdot \text{COVID Video} + \alpha \cdot \text{COVID Video} + \beta \cdot \text{AMARacism} + X'\gamma + \epsilon$$

- Question: Does the AMA racism acknowledgment heighten or dull any doctor concordance effects?
  Samples: COVID video groups. Separate analysis for black and white respondents.
  Regression:

  $$Y = \delta \cdot \text{AMARacism} \cdot \text{DocConcord} + \alpha \cdot \text{AMARacism} + \beta \cdot \text{DocConcord} + X'\gamma + \epsilon$$

  Alternate Specification: Again, we can also run the following modified regression, including both the COVID video and control groups.

  $$\begin{aligned} Y = \delta \cdot \text{DocConcord} \cdot \text{AMARacism} \cdot \text{COVID Video} + \lambda \cdot \text{AMARacism} \cdot \text{COVID Video} \\ +\phi \cdot \text{DocConcord} \cdot \text{COVID Video} + \rho \cdot \text{AMARacism} \cdot \text{DocConcord} + \alpha \cdot \text{COVID Video} \\ +\beta \cdot \text{AMARacism} + \psi \cdot \text{DocConcord} + X'\gamma + \epsilon \end{aligned}$$

4. **Does concordance of the AMA messenger matter?**

- Question: Does a race-concordant AMA messenger delivering a message about racial injustice make COVID messaging from doctors more or less effective, relative to a race-discordant AMA messenger?
  Samples: Individuals receiving an AMA message about racial injustice and a treatment message about COVID. Separate analysis for black and white respondents.
  Regression:

  $$Y = \beta \cdot \text{AMAConcord} + X'\gamma + \epsilon$$

  Alternate Specification: We can also run the following modified regression, the COVID video and control groups, with both types of AMA messages.

  $$\begin{aligned} Y = \delta \cdot \text{COVID Video} \cdot \text{AMAConcord} \cdot \text{AMARacism} + \lambda \cdot \text{AMAConcord} \cdot \text{AMARacism} \\ +\rho \cdot \text{AMAConcord} \cdot \text{COVID Video} + \psi \cdot \text{COVID Video} \cdot \text{AMARacism} \\ +\alpha \cdot \text{AMAConcord} + \beta \cdot \text{AMARacism} + +\phi \cdot \text{COVID Video} + X'\gamma + \epsilon \end{aligned}$$

[4] Ex ante, we think that an AMA level effect in the control group is more likely than a doctor concordance level effect. However, we include all alternates for completeness. These alternate specifications including the control group are relevant in the presence of a level effect.

- Question: Does a race-concordant AMA messenger make COVID messaging from a race-concordant doctor more or less effective, when delivering the AMA message about racial injustice?
  Samples: COVID video groups receiving the AMA message about racism. Separate analysis for black and white respondents.
  Regression:

  $$Y = \delta \cdot \text{AMAConcord} \cdot \text{DocConcord} + \alpha \cdot \text{AMAConcord} + \beta \cdot \text{DocConcord} + X'\gamma + \epsilon$$

  Alternate Specification: We can also run the following modified regression, the COVID video and control groups, with both types of AMA messages.

  $$\begin{aligned} Y = \delta \cdot \text{AMAConcord} \cdot \text{AMARacism} \cdot \text{DocConcord} \cdot \text{Covid Video} \\ +\alpha_1 \cdot \text{DocConcord} \cdot \text{AMARacism} \cdot \text{COVID} + \alpha_2 \cdot \text{DocConcord} \cdot \text{AMARacism} \cdot \text{AMAConcord} \\ +\alpha_3 \cdot \text{DocConcord} \cdot \text{COVID} \cdot \text{AMAConcord} + \alpha_4 \cdot \text{AMARacism} \cdot \text{COVID} \cdot \text{AMAConcord} \\ +\beta_1 \cdot \text{DocConcord} \cdot \text{AMARacism} + \beta_2 \cdot \text{DocConcord} \cdot \text{COVID} + \beta_3 \cdot \text{DocConcord} \cdot \text{AMAConcord} \\ +\beta_4 \cdot \text{AMARacism} \cdot \text{COVID} + \beta_5 \cdot \text{AMARacism} \cdot \text{AMAConcord} + \beta_6 \cdot \text{COVID} \cdot \text{AMAConcord} \\ +\rho_1 \cdot \text{DocConcord} + \rho_2 \cdot \text{AMARacism} + \rho_3 \cdot \text{AMAConcord} + \rho_4 \cdot \text{COVID} + X'\gamma + \epsilon \end{aligned}$$

5. **What are the effects of acknowledging racial disparities in COVID incidence?** In addition to the typical set of outcomes, we will also analyze effects on allocated donations to black-specific versus race-agnostic COVID-related charities.

   - Question: what is the main effect of acknowledging racial disparities of COVID incidence?
     Samples: treated respondents only. Separate analysis for black, white, and all respondents.
     Regression:

     $$Y = \beta \cdot \text{Vid2RacialDisp} + X'\gamma + \epsilon$$

   - Question: are race-concordant doctors more effective messengers about racial disparity?
     Samples: treated respondents only. Separate analysis for black, white, and all respondents.
     Regression:

     $$Y = \delta \cdot \text{DocConcord} \cdot \text{Vid2RacialDisp} + \alpha \cdot \text{Vid2RacialDisp} + \beta \cdot \text{DocConcord} + X'\gamma + \epsilon$$

   - Question: does acknowledging widespread racism alter the effectivness of later discussing racial disparities in COVID?
     Samples: treated respondents only. Separate analysis for black, white, and all respondents.
     Regression:

     $$Y = \delta \cdot \text{AMARacism} \cdot \text{Vid2RacialDisp} + \alpha \cdot \text{Vid2RacialDisp} + \beta \cdot \text{AMARacism} + X'\gamma + \epsilon$$

- Question: does a race-concordant AMA messenger's prefacing statement have a different amplifying effect than a race discordant AMA messenger? That is, if doctors are going to discuss racial disparities in COVID and we're going to preface this with the AMA racism statement, do we expect different results if the statement comes from a concordant versus discordant AMA messenger?

  Samples: Must have seen *both* a COVID video *and* an AMA racism video. Separate analysis for black and white respondents.

  Regression:

  $$Y = \delta \cdot \text{AMAConcord} \cdot \text{Vid2RacialDisp} + \alpha \cdot \text{Vid2RacialDisp} + \beta \cdot \text{AMAConcord} + X'\gamma + \epsilon$$

  Alternate specification: restrict sample to those seeing a COVID video, include both types of AMA messages. Regression:

  $$\begin{aligned} Y = \delta \cdot \text{AMARacism} \cdot \text{AMAConcord} \cdot \text{Vid2RacialDisp} + \lambda \cdot \text{AMAConcord} \cdot \text{Vid2RacialDisp} \\ + \rho \cdot \text{AMAConcord} \cdot \text{AMARacism} + \phi \cdot \text{AMARacism} \cdot \text{Vid2RacialDisp} \\ + \alpha \cdot \text{Vid2RacialDisp} + \beta \cdot \text{AMAConcord} + \psi \cdot \text{AMARacism} + X'\gamma + \epsilon \end{aligned}$$

5.4 Heterogeneous Effects

We plan to conduct several heterogeneity tests that we believe are of central importance: respondent race, respondent political afiliation (within the white sample), prior beliefs about racial disparities and COVID-19, and the timing of participation vis a vis the events of Kenosha, WI.
We are very interested in studying how the impacts of our treatments vary by the race of the respondent. This is central to our research design. Specifically,

- Is the impact of racial concordance different by respondent race?
- Is the impact of a statement addressing racial injustice different by respondent race?
- Is the impact of information about racial disparities in the COVID-19 burden different by respondent race?

Moreover, within the white respondent population, we predict that there may be substantial heterogeneity by the respondent's political beliefs:

- Is the impact of racial concordance different for white republicans versus white democrats?
- Is the impact of a statement addressing racial injustice different for white republicans versus white democrats?
- Is the impact of information on racial disparities in disease burden different for white republicans versus white democrats?

We predict that the impacts of information on racial disparities in COVID burden should depend on individuals' prior beliefs:

- The information about RD should cause individuals who initially believed at baseline that there were small or non-existant racial disparities to update in the opposite direction of individuals who believed that the racial disparities were larger than they actually are.
- Thus, for all of our tests involving RD, we will interact the regressions with indicators for whether the priors were smaller or larger than the number we give in the videos.
- Moreover, there may be bigger impacts for individuals whose priors were less accurate, so we can also interact by the size of the gap between the informaiton and the prior, separately for those with priors that were too low versus too high. This can also help us measure whether the information had an impact even for people with accurate priors, possibly through a salience effect.

We intend to compare the results of our hypothesis tests separately for the time period before the Jacob Blake police shooting in Kenosha, WI on August 23, 2020, and the time period after. We propose to do this for two reasons. First, we launched the study at a time when the large-scale protests from earlier in the summer following the murder of George Floyd had somewhat ebbed. So the events in Kenosha may bring issues of racial injustice to the fore. Second, and perhaps more importantly, polarization surrounding the narrative of the protests has markedly increased following the events of Kenosha. Several speakers at the Republican National Convention explicitly discussed the violent component of the protests, for example, and both candidates for the US presidency are making trips to Kenosha. This increased polarization is likely to have the most relevance for the white respondents in our study, and may enhance any differential response we find by political affiliation. We propose to split the sample into the period before August 23, 2020 and the period following August 26, 2020. We will omit surveys collected on days in the interim when Americans were only coming to learn about the events of Kenosha, WI.

We are also interested in secondary analysis exploring heterogeneity on the following categories of traits/characteristics:

- Age
- Level of baseline knowledge and health-preserving behaviors
- Place of residence (correlated with political affiliation, COVID-19 policies and phased reopenings, and socio-economic characteristics)

Given the many ways to cut the data for this secondary analysis, we will follow the methodology of Chernozhukov et al (2019) for this latter set of potential heterogeneous treatment effects.[5]

6. Robustness

6.1 Threats to Interpretation

We would like to assume that differences across videos come from either differences in the racial identity of the doctors in the video or from differences in the content of the messages, rather than from other chance differences across videos. Because we are including both white and black respondents who will be watching the same exact videos, we will be able to include video fixed effects in some specifications.

[5]Chernozhukov, Victor, Mert Demirer, Esther Duflo, and Ivan Fernandez-Val (2019). Generic machine learning inference on heterogenous treatment effects in randomized experiments. No. w24678. National Bureau of Economic Research.

6.2 Attrition

We have two separate endline surveys. The first will take place immediately after treatment delivery. The type of attrition that might arise here is through dropping out of the online session before completing all of the survey questions. To try to limit differential attrition, we are showing placebo videos to the control group to fill approximately the same amount of time.

Our second set of endline outcomes will take place a few days after the main survey. Lucid, the survey firm, will try to recontact a specified list of initial participants. We are only expecting modest recontact rates, and therefore high levels of attrition. Importantly, to try to limit differential attrition, all individuals will be recontacted with the exact same message, and it will not be made salient that the survey is a direct follow-up to the previous study.

We plan to test for differential attrition at both endlines across our key comparison groups.

7. Funding and Human Subjects Review

Funding is provided by the National Science Foundation RAPID-2029880 for Covid-19 research, and RAI Italian Broadcasting corporation (via an unrestricted gift to J-PAL that we attributed to this project). The IRB at MIT is serving as the primary institution of record and has entered into a reliance agreement with Harvard, Massachusetts General Hospital, and Yale. We have also received IRB approval from Stanford.

Appendix

A. Scripts

All respondents will receive either [Statement RI or Statement DP] + one set of [Treatment or Control] videos.

A.1 Statements

Each respondent is assigned to one of the following statements. All respondents will see the statement presented via video.

A.1.1 Treatment Statement RI (Racial Justice):

- The American Medical Association recognizes that racism in its systemic, structural, institutional, and interpersonal forms is an urgent threat to public health, the advancement of health equity, and a barrier to excellence in the delivery of medical care.
- The American Medical Association opposes all forms of racism.
- The American Medical Association denounces police brutality and all forms of racially-motivated violence.
- The American Medical Association will actively work to dismantle racist and discriminatory policies and practices across all of health care.

A.1.2 Placebo Statement DP (Drug Pricing):

- The American Medical Association believes in transparency in prescription drug pricing, and we are pleased the House Ways & Means Committee moved the issue forward.
- Patients and their physicians want to be armed with more information, yet the current situation is opaque if not impenetrable.
- The committee is rightfully determined to expose factors that lead to high drug prices, and we look forward to continuing our efforts in that regard.

A.2 Treatment Videos about COVID-19

A full set of treatment videos includes T1 + T2 + [T2A or nothing] + T3

Video T1:

Hello, I'm Dr [YOUR LAST NAME HERE] from [YOUR INSTITUTIONAL AFFILIATION HERE], and I?d like to tell you a little about Coronavirus or COVID-19. COVID-19 is a new virus that can infect the respiratory tract and lungs. Although many people who get sick from COVID will get better, some people who get it become very ill and some even die.

Although there's no cure, there are ways medical professionals have found to protect you and your community from COVID. I hope that this message can give you information that will help you protect you or someone you love from COVID infection.
First, I would like to tell you about the symptoms of COVID-19. The most common symptoms of COVID-19 are cough, fever, and trouble breathing. Another odd symptom some people have is loss of taste or smell. A large number of people who have COVID-19 actually don't show any symptoms at all. Unfortunately, people can still spread the disease to others even with no symptoms. The next video will provide you with more information on how you can protect yourself and others.

Video T2:

Hello, I'm Dr [YOUR LAST NAME HERE] from [YOUR INSTITUTIONAL AFFILIATION HERE],
You may be looking for ways to resume some activities as safely as possible.
However, COVID-19 remains contagious and shows no signs of disappearing. In fact, during the week of July 6 there were 58,000 new COVID cases per day diagnosed in the United States.
[ONLY FOR ACKNOWLEDGMENT SUB-TREATMENT T2A] *Black Americans and other minority groups are three times as likely to get and, when you account for age, four times as likely to die from COVID as white Americans.*
Without a safe and effective vaccine or therapy, our only option is to continue taking precautionary measures to protect ourselves, our communities, and the most vulnerable among us.
While there is no way to ensure zero risk of infection from COVID-19, observing these three practices will help to protect you and others.
First, continue to practice social distancing whenever possible: Try to stay outdoors, and to the maximum extent possible, please stay 6 feet apart. If you must be indoors, use visual reminders—like signs, chair arrangements, markings on the floor, or arrows—to help remind you to keep your distance from others, and maintain physical barriers whenever possible.
Second, continue to wash your hands often for at least 20 seconds with soap and water, especially before going out, and every time you return home.
Third, wear a mask when in public at all times, especially when indoors or when it is difficult to stay 6 feet away. The next video will tell you a bit more about masks.

Video T3:

Hello, I am doctor [YOUR LAST NAME HERE] from [YOUR INSTITUTIONAL AFFILIATION HERE], and I will tell you a bit more about masks. Wearing a mask is a key way to prevent the spread of COVID-19. You are not just protecting yourself but also your grandma and your community, just in case you have COVID-19 but don't know it.
Even if wearing a mask may sometimes put you in a difficult situation, it is important to protect you and the community from COVID 19 disease. As medical professionals, I am committed to delivering the best care I can to every patient. My goal is to make sure that you and everyone you love survives this COVID-19 pandemic. Thank you for listening to these messages.

A.3 Control Videos about non-COVID-19 Health Behaviors

A full set of control videos includes C1 + C2 + C3

Video C1:

Most adults need to sleep between 6 and 8 hours a night. Now, there are some people who get five hours a night and they are fine, so there is some variation across people. But for most adults, we need 6 to 8 hours in order to function well the next day. If you feel sleep deprived you might not be able to function as well as you would normally like.
It's important to have something called sleep hygiene which is a routine you follow at bedtime and can help you fall asleep. Things that can disrupt sleep hygiene include caffeine or alcohol too close to bedtime. Eating late at night can also cause indigestion. So keep a routine and trying to get 6-8 hours is important.

Video C2:

Sugar is found in many different food items. Natural sugars are those that can be found in fruits, vegetables and dairy products like milk. Sugars like these that are natural are not really problematic because they are coming alongside lots of other vitamins and minerals.
There are other sugars, though, that are processed and added to a food item. These are called additive sugars. A good rule of thumb is to eat foods with less than than 5g of sugar per serving. Avoid buying products where one of the first five products is a sugar.
And it can be better to buy an unsweetened product like an unsweetened cereal or oatmeal and then add a teaspoon of sugar to it if you need the sweetness than to buy a heavily sweetened product, like a sugar cereal which can have several teaspoons of sugar per serving.

Video C3:

New fitness guidelines can be summed up as follows: just move and anything counts. Sneaking in a few minutes of physical activity throughout the day adds up in the long run. The guidelines are trying to make it easier for individuals to be fit and drop the rule that activity must be in 10 minute blocks of time. In a nutshell, activity has benefits even if it's for a short amount of time. Taking the stairs instead of the elevator, parking your car far away from the entrance to a store or walking your dog around the block can all help you be fit. The guidelines still call for at least 150 minutes a week of moderately intense aerobic exercise and two weekly sessions of muscle training activity, like lifting weights or yoga.

Figure 2: Fully extended randomization tree

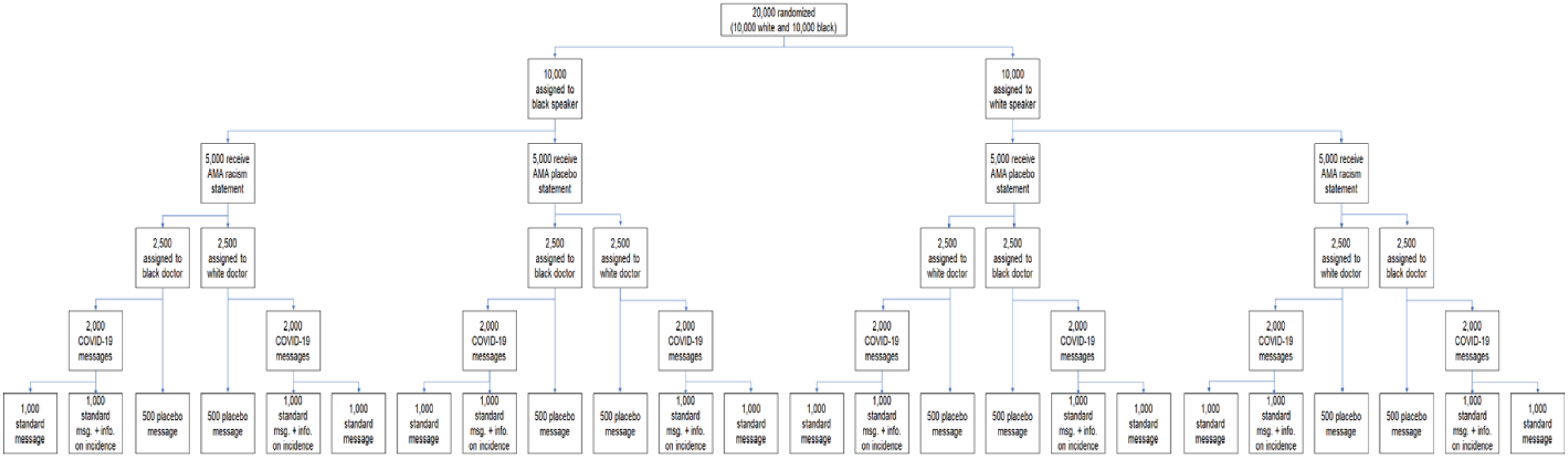

	Massachusetts Institute of Technology<br>Committee on the Use of<br>Humans as Experimental Subjects<br>COUHES	**COUHES Protocol #**<br>2003000118<br><br>Updated July 13, 2020

APPLICATION FOR COMPREHENSIVE REVIEW

Please complete all questions and provide sufficient detail. Indicate 'N/A' if a question does not pertain to your research. An incomplete application will be rejected and returned for completion.

I. BASIC INFORMATION

**1. Title of Study**	
Using Social Media to Spread Public Health Messages for COVID19	
**2. Principal Investigator**	
Name: Benjamin Olken	Building and Room #: E52-542
Title: Professor of Economics	Email: bolken@mit.edu
Department: Economics	Phone: 617-253-6833

3. Funding

If the research is funded by an outside sponsor, please enclose one copy of the research proposal with your application. A draft of the research proposal is acceptable.

Do not leave this section blank. If your project is not funded, check No Funding in section C.

A. Sponsored Project Funding:

☐ Current Proposal Grant/Proposal # ________________________
Sponsor _____
Title _____

☐ Current Award Grant/Account # _______________
Sponsor ___ JPAL Innovation in Governance Initiative ___

Title _____ Using Social Media to Spread Public Health Messages for COVID19

X Current Award Grant/Account # _______________
Sponsor ___ NSF ___
Title: RAPID: Covid-19 Information Campaigns for Vulnerable populations

B. Institutional Funding:

☐ Gift ☐ Departmental Resources
X Other (explain) _Esther Duflo MIT discretionary account____

C. No Funding

☐ This protocol will not be funded

4. Statement of Financial Interest

A. Does the investigator, study personnel, or their Family have a financial interest in a company or other organization involved in this study?
☐ Yes X No

B. Could the work contemplated in this project reasonably appear to affect a company or other organization in which the investigator, study personnel, or their Family have a financial interest?
☐ Yes X No

C. Does this study contemplate:

i. Receiving or using any data (e.g., proprietary data sets, data sets, confidential information) from a company or other entity organization in which the investigator, study personnel, or their Family have a financial interest
☐ Yes X No

ii. Receiving or using any materials (e.g., drugs, devices, biological agents, investigational medical devices) from a company or other entity organization in which the investigator, study personnel, or their Family have a financial interest
☐ Yes X No

iii. Granting subawards to a company or other entity organization in which the investigator, study personnel, or their Family have a financial interest
☐ Yes X No

iv. Making purchases from a company or other entity organization in which the investigator, study personnel, or their Family have a financial interest
☐ Yes X No

If 'yes' was checked for any of the questions above, then attach a **Supplement for Disclosure of Financial Interest** for each individual with an interest. This supplement and detailed guidance are available on the COUHES website under Policies & Procedures in the Financial Conflicts of Interest section.

5. Anticipated Dates of Research	
Start Date: 3/16/2020	Completion Date: 12/31/2024

6. Collaborating Institutions
If you are collaborating with another institution(s), then you must obtain approval from that institution's institutional review board (IRB) and forward the approval to COUHES.

Harvard University
Yale University
Stanford University
Massachusetts General Hospital
(MIT will be prime IRB, and we will request these other institutions cede to MIT)

7. Location of Research

If on the MIT campus, indicate where on campus. If you plan to use the facilities of the Clinical Research Center you will need to obtain approval of the MIT Clinical Research Center.

Location of the research will depend on the country site. All US research will take place online. Moreover, any time we use a social media platform such as Twitter, YouTube, or Instagram, the research will all be online.

For the India component of the research targeting rural areas or low income urban neighborhoods, the research will be done all by phone. This is because in much of India, the main social media platform is WhatsApp, which does not have a centralized platform. Instead individuals form size-limited groups and forward information to group members. None of this information is public. So we will use phone surveys to measure the outcomes of the research.

8. International

Research conducted outside the United States may be subject to additional requirements.

A. Are you collecting or receiving identifiable data from subjects within the European Union (EU) and/or European Economic Area (EEA)?

☐ Yes X No

B. Is the project in, related to, or funded by a person or entity from China (including Hong Kong), Russia or Saudi Arabia?

☐Yes X No

If yes, additional review and approval is required. Please see *Additional Review* *for additional information.*

II. STUDY INFORMATION

1. Purpose of Study

Provide a concise statement of the background, nature and reasons for the proposed study. Use non-technical language that can be understood by non-scientists.

Given the developing global coronavirus pandemic, we propose to expand our previous research in Indonesia (COUHES Protocol # 1406006433), which shows that celebrities have an important role in public health messaging, to encouraging COVID19 preventative health measures. 1) We plan to recruit a new set of social media influencers in the U.S. and other countries in a similar fashion to what we did in Indonesia, but this time with all of the messaging being about coronavirus (e.g., social distancing, hygiene). 2) We also plan to conduct surveys with a random sample of followers of the social media influencers participating in the Covid-19 awareness campaign. The purpose of this is to allow us to understand which messages work in real time and continually improve them. We are including new recruitment scripts for both the influencers and the followers with the consent scripts.

We are also expanding the initial research idea to focus on how the medical community can best reach vulnerable populations of color through short online videos (US Doctors Videos).
2) We have teamed up with physicians from Massachusetts General Hospital (MGH) to design video messages to reach communities of color and to speak to their specific concerns.
3) Because we know that video messages cannot be too long and we do not know which types of message content reaches the target population in the most impactful, sincere and trustworthy way, we are testing many different variants of the basic message. Experimental manipulations include a) varying the racial and ethnic identity of the doctor in the video; b) varying whether the medical professional addresses the so-called "elephant in the room" pertaining to historical inequitable treatment and lack of

trust in the medical community or pertaining to deportation fears when seeking healthcare; c) specifically for masks – whether we provide information from a nationally representative group of respondents on perceptions of people of color wearing masks.

Drawing on the initial round of US Doctors Videos research, we intend to launch a phase 2 project (US Doctors Round 2) to be able to answer an additional set of related questions:
4) Do the impacts of concordance look different during Summer 2020, post-George Floyd? The country has moved very quickly on some issues involving race in America. In order to be able to advise governments and public health organizations on messaging for minority communities, it is important that we re-run a component of our study in the current moment.
5) What are the impacts of racial concordance on white subjects?
6) Following the widespread protests of June 2020, many organizations have issued statements about racial injustice. The American Medical Association is one organization that has done this. We would like to understand how these types of statements affect the impact of whatever messaging follows (in our case, information about Covid-19). Does the impact of these statements vary by the race of the messenger, the race of the viewer, or the political affiliation of the viewer?
7) An article in the July 6 2020 New York Times documents that African Americans are 3 times more likely to contract Covid-19 than white Americans. How does acknowledging this fact affect the impact of messaging on Covid-19. Again, does the impact of hearing this information vary by the race of the messenger, the race of the viewer, the political affiliation of the viewer, and prior beliefs about Covid-19 incidence?

We are also expanding our research to India where the difference in social media usage requires a different research strategy:
48) We plan to recruit celebrities to record video messages about Covid19.
9) We will then distribute the messages to individuals that we have identified as information hubs in local communities. We will send the videos to these information hubs and ask them to share the messages on WhatsApp with others in the community. We will tell them not to share the messages via face-to-face interactions.
10) We plan to conduct surveys with others in the study communities to understand a) if they have seen the messages and b) measure their beliefs about Covid19 their practice of social distancing and good hygiene, and their financial situations.
11) Because of the importance of frontline health workers (FHWs) in the official, rural response, we are also planning to conduct complementary surveys in the study areas. This will help us understand the impacts of the information intervention.
12) We plan to also launch a "light-touch" intervention with the FHWs themselves. This will include setting up WhatsApp groups comprised of FHWs from other villages in the same state. We would encourage them to support one another and to share information about their own experiences. We will also study how well the groups work if we additionally: i) give information about how confusing and fast-paced the landscape is (giving a license to ask to FHWs) or ii) give information about common rumors that our household surveys uncover or that the media reports in the local areas. (Health Worker Script)

2. Study Plan

This section determines if the study plan meets the Federal definition of a clinical trial. COUHES will assist with any additional requirements based on the responses below. For more information available on COUHES website for Clinical Trials: http://couhes.mit.edu/clinical-trials-mit

A. Are the participants prospectively assigned to an intervention?
X Yes ☐ No

B. Is the study designed to evaluate the effect of the intervention on the participants?
X Yes ☐ No

C. Is the effect being evaluated a health-related biomedical or behavioral outcome?
X Yes ☐ No

3. Experimental Procedures

Provide an outline of your experimental procedures with a detailed description of your proposed study. When applicable, include copies of any questionnaires or standardized tests.

Do not attach or copy sections of a grant application.

When applicable, include a detailed description of the experimental devices or procedures, detailed information on the exact dosages of drugs or chemicals to be used, total quantity of blood samples to be used, and descriptions of any special diets.

Provide sufficient information for effective review by non-scientists. Define all abbreviations and use simple words. This section should not exceed 5 pages unless justification is provided for additional length.

The following procedures are for sites where Twitter, Instagram, or YouTube are the relevant social media platforms. This pertains to the US, for example.

We will recruit influencers -- i.e. people with large social media followings -- and encourage them to share public health guidance about COVID19. The influencers have full discretion about what they post on social media, but we plan to give randomized recommendations about the content of each message to understand which messages are most effective.

All of the recommended message content will be vetted by the MD on our study team. All of the messages will be consistent with the recommendations of the CDC or equivalent public health authorities in other countries. We are attaching a set of sample instructions for the influencers consistent with this design.

The different treatments will vary the emphasis of the message in four different dimensions to understand which types of messages are most effective, so that we can encourage more of the most effective message types:

1. Target behavior: Messages will be requested to emphasize either social distancing or hygiene.
2. Rationale for the behavior: Messages will either emphasize the internality (the benefits accrue to the person doing that behavior) or the externality (the benefits accrue to others in the community)
3. Timing of the messages: We will vary the order and the variety of the content that we request people to tweet about.

4. Numbers versus narrative: We will emphasize the way the message is delivered in terms of its emphasis on the numbers versus the human narrative behind that feature.

We will also include a fifth randomized nudge:

5. Video vs. written message. We will give the influencer full discretion over this choice.

We are also planning to conduct *online* surveys with a random set of followers of each of the participating influencers. We would like to survey the followers repeatedly. This information will be the key outcomes that allow us to understand the impacts of the messages, so that we can dynamically adjust the messages to focus on those messages which appear to be most effective. The survey questions are attached.

US Doctors Study (Round 1)

After receiving approval in our last IRB amendment, we quickly ran a nationally representative pilot survey to understand current knowledge gaps about covid and also to measure people's perceptions of others, especially those belonging to communities of color, when wearing masks.

After running this pilot, we would like to launch a video messaging intervention aimed at communities of color. In the past few weeks, data has shown that covid19 is disproportionately infecting and killing African Americans and Latinx people. We have teamed up with physicians from MGH to develop video messages targeted at these groups. The doctors bring their expertise care providers for a diverse patient population.

The following procedures will be followed. The research subjects will participate online.

1. Doctors from MGH who express interest in participating will record messages according to our scripts (**US Doctors Script African American, US Doctors Script Hispanic**). They will be instructed to film several different versions each.
2. We will recruit a sample of Hispanic and African American study participants from across the country through Lucid, an online survey firm that has access to a large subject pool.
3. Participants will first read a consent script and give us their informed consent (**US Doctors Messaging Consent**)
4. Participants will then navigate through the following steps
    a. Brief demographics survey questions (US Doctors Messaging Survey)
    b. Videos 1 and 2: Introduction, Elephant in the Room, Social Distancing (**US Doctors Script African American, US Doctors Script Hispanic**). Individuals in the control group will only see the introduction portion of the videos.
    c. Beliefs survey questions (US Doctors Messaging Survey)
    d. Video 3: Masks (**US Doctors Script African American, US Doctors Script Hispanic**). Again, individuals in the control group will not see a video here.
    e. Main outcomes survey (**US Doctors Messaging Survey**)
    f. Control group is shown a version of all 3 videos (**US Doctors Script African American, US Doctors Script Hispanic**).
5. The video messages each participant in one of the treatment groups sees will be randomized on the following dimensions. The different versions are clearly demarcated in the US Doctors Scripts document.

a. Racial or ethnic identity of the doctor delivering the messages: concordant vs. discordant identity to the subject.
b. Whether the message includes an acknowledgment of "elephant in the room" issues for each target group: trust in the medical system or fear of deportation.
c. Whether the social distancing component of the message is delivered by Dr. Birx of the CDC or recorded by the MGH physicians.
d. Whether individuals are given information about how representative individuals perceive mask wearers of color. This information comes from results of our nationally representative pilot survey.
e. Some individuals in a control group will only see messages after all surveying has been completed.

US Doctors Study (Round 2)

After completing the initial US Doctors Study, we learned that racial concordance is extremely important for African American viewers. We would like to run a similar video messaging study with very similar protocols. Our goal is to better understand the impacts in the context of July 2020. The past 6 weeks have seen large scale protests and rapid social change. Do the strategies that worked in early May still work now? We would also like to study new messaging variants – namely 1) what is the role of statements addressing racial justice? 2) what is the role of making racial disparities in the incidence of Covid-19 salient in the videos?

The following procedures will be used:

1. Doctors from MGH who express interest in participating will record several new messages according to our scripts (**US Doctors Script Round 2**).
2. We will recruit a sample of white and African American study participants from across the country through Lucid, an online survey firm that has access to a large subject pool. We will stratify treatments on age, education, geographical location and political leaning.
3. Main study session
    a. Participants will first read a consent script and give us their informed consent (**US Doctors Messaging Consent Round 2**). Consent will pertain to the main study session and to the follow-up survey activities. The consent text will also inform individuals that if they provide their consent, they might be recontacted in the future.
    b. Participants will then answer a short set of baseline questions
    c. Treatment videos are shown (see below)
    d. After each video, respondents give evaluations (see **US Doctors Survey Round 2**)
    e. Participants answer a set of Endline questions (**US Doctors Survey Round 2**)
4. Several days after the main study session, Lucid will attempt to recontact all of the initial participants for follow-up activities.
5. Follow-up session
    a. Follow-up survey (**US Doctors Survey Round 2**)

Treatment variants: All respondents (including the control) will initially view one of two messages with content developed by the AMA

- Racial injustice (treatment of interest) or drug pricing (placebo)
    - The race of the doctor in the video message will be randomized

- Some respondents will see a video of the physician speaking. Others will only hear that physician's voice.

All respondents will then be shown the main video messages

- Control: One meaningful difference in round 2 compared to round 1 is the protocol for the control group. The control group will be shown video messages with content developed by the AMA, the Mayo Clinic or Mount Sinai Hospital on topics about health that do not directly relate to Covid-19.
    - The race of the doctor in the video will be randomized across participants
- Treatment: Subjects will watch three videos about Covid-19 in short succession.
    - Content
        - i. Video 1: introduction to Covid-19 and its symptoms. We will use the same videos here that were recorded for the prior iteration of the study.
        - ii. Video 2: social distancing and hygiene. We are recording new videos here because now many states have reopened and the formal guidance is different.
            1. We will randomize from two different versions of this video. There will be a baseline version and a version that additionally emphasizes that Black Americans and other minority groups are three times as likely to get Covid-19 as white Americans.
        - iii. Video 3: masks. Again, we will use videos that were recorded for the prior iteration of the study.

The racial concordance between the doctor and the viewer will be randomized. The race of the doctor will be the same across all three videos.

The following procedures are for sites where WhatsApp is the relevant social media platforms (e.g., rural and low income urban India):

The messages have been approved by Marcella Alsan MD from Harvard University and by Abhijit Chowdhury, MD from Birbhum Health and Demographic Survey System, India.

1. Recruit major celebrities: Given the context, we do not need many celebrities. In fact, we only require a minimum of one celebrity, though we certainly welcome more than one.
2. Record messages: Ask each celebrity to record a "bank" of 16 messages.
    a. See attachment (India Celebrity Guidance)
3. Treatments: We will have 3 dimensions of treatment differences:
    a. Internality vs Externality
    b. Hygiene vs Social Distance
    c. Type 1 vs. Type 2 Error (Type 1 error: individuals with symptoms need not have covid, so it's okay to report. Type 2 error: individuals without any symptoms may still have covid, so it's even more important to practice hygiene and social distancing.)
4. Selection of information hub: contact individuals in each study village and ask them to name the person that they would recommend for spreading trusted information, who also has a smartphone (Initial Contact Script and Survey).
5. Intervention: in every period we

a. Choose a set of villages to receive a(nother) message (villages will receive many messages over time.)
b. Seed the gossip with one of the videos in the message bank. See attachment (Seeding Script)
c. Make the info drop "common knowledge" among our outcome sample individuals.

6. Surveys: In each survey round, we contact a randomly chosen subset of all sampled individuals in each village and conduct the survey over the phone. See attachment (IRB India Phone Survey). Surveyors will use tablets to record answers. However, if we run out of tablets, or the tablets malfunction, they will use paper surveys.

4. Drugs and Devices

If the research involves the administration of a novel drug not approved by the Food and Drug Administration (FDA) for the use outlined in the protocol, then the principal investigator (or sponsor) must obtain an Investigational New Drug (IND) approval from the FDA. If the study involves the use of an approved drug in an unapproved way, the investigator (or sponsor) must submit an application for an IND approval. If applicable, include a copy of the IND approval (new drug) or application (new use).

If the study involves the use of a novel medical device and the device poses significant risk to human subjects, the investigator (or sponsor) must obtain an Investigational Device Exemption (IDE) approval from the FDA.

COUHES may determine an IDE or IND is appropriate during review.

A. Will drugs or biological agents requiring an IND be used? ☐ Yes X No
If yes, please provide details:

B. Will an investigational medical device be used? ☐ Yes X No
If yes, please provide details:

5. Radiation

Research involving the use of radiation or radioactive materials may require review from MIT's Environment, Health, and Safety (EHS) Office. COUHES may determine EHS review is appropriate.

A. Will radiation or radioactive materials be used? ☐ Yes X No
If yes, please provide details:

B. Will any type of lasers be used? ☐ Yes X No
If yes, please provide details:

6. Diets

A. Will special diets be used? ☐ Yes X No ,*If yes, please provide details:*

III. PERSONNEL

Fill out the personnel list at the end of this form.

IV. HUMAN SUBJECTS

1. Subjects
The number of subjects must corresponded with the maximum number of subjects investigators will consent for the study.

A. Maximum number of subjects:	B. Specify age range(s):
Adults: 112,500 Minors:<br><br>India: We are planning to recruit subjects in 5000 villages. This will mean 50,000 total participants in the India site, including initial contacts, seeds, and outcome survey participants.<br><br>5,000 frontline health workers<br><br>We would like to run a pilot online survey in the US with 1,500 individuals.<br><br>We would like to conduct the two phases of the US Doctors messaging intervention with 26,000 individuals in total. (Round 1 recruited approximately 11,000 respondents. We hope to target an additional 15,000 respondents in Round 2)	Adults: 18+ Minors:

C. Inclusion and exclusion criteria:

i. What are the criteria for inclusion or exclusion?
Twitter, YouTube, Instagram sites: Adults who are followers of influencers who join our campaign.

For our pilot survey for the MGH doctor's project, we plan to survey 1,500 individuals using a sample provided to us by Lucid. We would like to oversample Hispanics and African Americans with High School education or below. We believe that these groups face the largest information gaps while also shouldering a disproportionate burden from covid19.

For the main US Doctors messaging study (Round 1) we will recruit a sample from Lucid of African Americans and Latinos with High School education below. This is the group that we believe are hardest to reach with current public health messaging campaigns and who bear the most disproportionate impact from covid19.

For the main US Doctors messaging study (Round 2) we will try to recruit a sample from Lucid of African Americans and white Americans with below college education. We plan to achieve balance in the white sample on political ideology. Basic demographics about the sample will be shared with us by Lucid.

WhatsApp sites: WhatsApp does not allow us to view any information about users, publicly. Also, in the current climate it is not appropriate to make face-to-face contact with participants. Therefore, we need to recruit individuals in rural villages or urban neighborhoods whom we can contact through by phone through any of the following methods:

1. Contact individuals who participated in prior studies involving this study's investigators and who shared their phone numbers with us.
2. Contact beneficiaries of partner NGOs such as Pratham India who are willing to share their information with us.
3. Contact local elected politicians (ward members). The phone numbers of ward members are publicly available.
4. Ask individuals we contact through all of the above channels to give us contact information of other people they know, building out a so-called "snowball" sample.

FHW activities: In each study village, we will seek to enroll the government front-line health worker in the study.

ii. Are any inclusion or exclusion criteria based on age, gender, or race/ethnic origin? (*Investigator must explain why and provide justification.)* No.

iii. Explain the inclusion of any vulnerable population(s) (e.g. children, cognitively impaired persons, educationally disadvantaged persons, non-English speakers, MIT students) and why. COVID-19 is a global problem. We are interested in social media campaigns in the US, but we also think that the methodology and perhaps lessons from the study can immediately be deployed in other places where the spread is in an earlier stage, including Indonesia and India. We will conduct surveys in the local languages.

2. Subject Recruitment

Identification and recruitment of subjects must be ethically, legally acceptable, and free of coercion. Describe below what methods will be used to identify and recruit subjects. Include copies of recruitment documents (i.e. flyers, e-mails, advertisements, etc.).

Twitter, YouTube, Instagram sites:

We are planning to conduct *online* surveys with a random set of followers of each of the participating influencers. Lists of followers on the social media platforms we plan to study are public, and we will send direct messages on the social media platform to recruit participants. We are including a sample recruitment email as an attachment.

MGH Doctors project (baseline survey): We will use a sample provided to us by Lucid.

MGH Doctors messages experiment (Rounds 1 and 2): We will use a sample provided to us by Lucid, specified to our target demographics, above.

WhatsApp sites:

WhatsApp is not a centralized platform. Instead, individuals join groups (which cap the number of members) and then frequently share video content across these groups. Our unit of recruitment will be the village or the urban neighborhood (we often use the term village, but in urban settings this refers to neighborhood). We will identify one person of high network centrality within each village through phone surveys. Then this individual will be "seeded" with a video created under our guidance by a celebrity.

Given the WhatsApp context, we need to recruit villages, "seed" individuals, and outcome survey respondents ourselves, as there is no clear set of social media followers to study. Moreover, we are not able to do door-to-door recruitment during the current public health environment. Therefore all recruitment must be done over the phone. We propose to recruit participants in four ways:

1. Contact individuals who participated in prior studies involving this study's investigators and who shared their phone numbers with us.
2. Contact beneficiaries of partner NGOs such as Pratham India who are willing to share their information with us.
3. Contact local elected politicians (ward members). The phone numbers of ward members are publicly available.
4. Ask individuals we contact through all of the above channels to give us contact information of other people they know, building out a so-called "snowball" sample.

Once we have received any contact information for a given village we plant to recruit:

1. At least one "seed" individual who will be given the video message by text directly from a surveyor. He/she will be asked to spread the message using WhatsApp. (We have several messages instructing individuals to only share the information via Social media, not through face-to-face interactions or phone sharing. This individual will be selected by asking the phone contacts we have access to the following question: Can you please think of all of the people in this village who you know with smartphones. Which of these people would be best at informing the largest number of people about a new job opportunity, a fair or a festival, or important health information?
2. Several (between 3 and 30) "outcome survey respondents." We will sample from all of the phone numbers we can manage to collect in the village though all of the strategies listed above.

For the FHWs, we will use publicly available phone numbers to contact them initially. We will go through a consent process before conducting any surveys or interventions.

3. Informed Consent

Informed consent is required from all human subject research studies involving participants. Templates are available on the COUHES website under Forms & Templates (https://couhes.mit.edu/forms-templates). *Under very limited circumstances, COUHES may waive the elements or requirement for informed consent. If you are requesting a waiver or alteration of consent, include the Waiver or Alteration of Informed Consent Request form.*

Attach informed consent form(s) with this application.

4. Subject Compensation
Payment must be reasonable in relation to the time and trouble associated with participating in the study. It cannot constitute an undue inducement to participate.

A. Describe all plans to pay subjects in cash or other form of payment:

Twitter, YouTube, Instagram sites:
None. Subjects will not be compensated in general, but we will have a lottery for one subject respondent to win an iPad. The online surveys will be short and costs of participation are minimal.

MGH doctors project (Round 1 and Round 2): Lucid will compensate participants their standard, agreed-upon rates. In the Round 2 study, we will also assess each individual's willingness to pay for masks. Individuals will be asked their valuation for 2 reusable masks. Some individuals will randomly be chosen to receive a prize worth at most $30, either paid as an Amazon gift card or a coupon for 2 masks from an online mask store. We will not collect any PII to execute this reward system. Individuals will be given single use coupon codes. We will not know the identity of the users.

WhatsApp sites:
None. There will be no compensation to participants.

B. Will subjects be reimbursed for travel and expenses?
N/A

5. Potential Risks
A risk is a potential harm that a reasonable person would consider important in deciding whether to participate in research. Risks can be categorized as physical, psychological, sociological, economic and legal, and include pain, stress, invasion of privacy, embarrassment or exposure of sensitive or confidential data. All potential risks and discomforts must be minimized to the greatest extent possible by using e.g., appropriate monitoring, safety devices and withdrawal of a subject if there is evidence of a specific adverse event.

A. What are the risks/discomforts associated with each intervention or procedure in the study
We believe risks to be minimal. All suggested messages will be vetted by the MD(s) on our team and in accordance with CDC guidelines.

For the India site, we are also running all of our materials by a MD based in Kolkata, India to ensure that our guidance is consistent with local practice.

In this current climate, we do not understand which types of messages are more successful at informing rural households or encouraging behaviors like social distancing and hygiene. After consulting with local experts, we believe that each message could be beneficial ex ante. For the social media and India projects, we plan to use an adaptive research design to dynamically learn about which treatment is the best. Once we learn which one is the best, we will only distribute the best message(s). If we find that some treatment is doing particularly badly (or even might be backfiring) we will discontinue the use of that treatment. Moreover, we will give the more

effective videos to all study villages once we learn what that treatment is.

B. What procedures will be in place to prevent/minimize potential risks or discomfort?
All suggested messages will be vetted by the MD on our team and in accordance with CDC or other appropriate local guidelines. Survey participants can skip any question they choose and withdraw participation at any time.

6. Potential Benefits

A. What potential benefits may subjects receive from participating in the study?
We aim to encourage compliance with CDC guidelines about hygiene, mask-wearing and social distancing, reducing the spread of COVID19. We also believe that our treatments might help to make the FHWs more effective.

B. What potential benefits can society expect from the study?
We aim to encourage compliance with CDC and other local guidelines about hygiene, mask-wearing, and social distancing, reducing the spread of COVID19. Also we aim to understand how to encourage information sharing among FHWs, who are stretched very thin during a health crisis.

7. Data Collection, Storage, and Confidentiality

A. How will data be collected?

Twitter, YouTube, Instagram sites and US Doctors messaging project:
Online survey platform (Qualtrics or equivalent, Lucid)

WhatsApp sites:
Phone surveys. The enumerators will record answers using SurveyCTO on tablets. If we run out of tablets, or the tablets malfunction, the enumerators will record responses on paper surveys.

B. Is there audio or videotaping? ☐ Yes X No
Explain the procedures you plan to follow:

C. Will data be associated with personal identifiers or will it be coded?
☐ Personal Identifiers X Coded
Explain the procedures you plan to follow.

Twitter, YouTube, Instagram sites:
We will collect email addresses and social media user names to follow up with participants since we will survey the same respondents multiple times. We will separate out identifiers from the coded dataset and analyze data on the de-identified dataset.

MGH doctors project pilot:
We will collect phone numbers to be able to contact respondents in the future. We will separate out identifiers from the coded dataset and analyze data on the de-identified dataset.

MGH Doctors Messaging Project:

We are currently not planning to collect any contact information or personal identifiers from participants. If we decide to collect identifiers such as phone numbers or email addresses, we will apply for an amendment. This is also true in Round 2. Unlike the previous round, we plan for Lucid to do the re-contacting of respondents for the follow-up survey. This means that we still will not need to collect PII from the respondents. We will only share coded identifiers with Lucid.

Whatsapp sites:
We will collect names and phone numbers to follow up with participants since we will survey the same respondents multiple times. We will separate out identifiers from the coded dataset and analyze data on the de-identified dataset.

D. Where will the data be stored and how will it be secured?
All identifiers will be kept in Veracrypt-encrypted dropboxes.

E. What will happen to the data when the study is completed?
All PII will be destroyed once the study is complete.

F. Can data acquired in the study affect a subject's relationship with other individuals (e.g. employee-supervisor, patient — physician, student-teacher, family relationships)?
N/A

8. Deception
Investigators must not exclude information from a subject that a reasonable person would want to know in deciding whether to participate in a study.

A. Will information about the research purpose and design be withheld from subjects?
☐ Yes X No
If yes, explain and justify:

9. Adverse Effects
Serious or unexpected adverse reactions or injuries, and/or unanticipated problems involving risks to subjects or others must be reported to COUHES within 48 hours. Other adverse events should be reported within 10 working days.

A. What follow-up efforts will be made to detect any harm to subjects, and how will COUHES be kept informed?
We do not expect harm from the study.

10. Health Insurance Portability and Accountability Act ("HIPAA")
If your study involves individually identifiable health information and is sponsored by MIT Medical, an MIT Health Plan or another healthcare provider, then you must complete the questions below because HIPAA likely applies to your study. For more information regarding the applicability of HIPAA to human subjects research, please click here.

A. Do you plan to obtain, use or disclose identifiable health information in connection with your research study?
X Yes ☐ No

While the study is about COVID-19, we are not asking participants any information about their own health status in the celebrities studies.

In the MGH doctors project pilot and the main messaging project, we are asking an individual about his/her covid19 status and about pre-existing conditions that are co-morbidities for covid19. However, we are not obtaining health information from any third party.

If YES, then all participants must complete an Authorization for Release of Protected Health Information Form. Please attach a copy of this draft form. You must use the template available on the COUHES website.
Alternatively, COUHES may grant a Waiver of Authorization in certain very limited circumstances when use of individually identifiable health information would pose only minimal risk to study participants (among other requirements). For additional information regarding whether your study might qualify for a waiver, please click here.

B. Are you requesting a Waiver of Authorization?
☐ Yes X No ☐ N/A

If yes, explain your rationale for concluding that:
(i) *use of participant health information poses no more than minimal risk;*
(ii) *the research could not be conducted without the waiver and*
(iii) *the research could not be conducted without the information.*

In addition, please explain your plan for (i) ensuring the participant health information is not improperly used or disclosed either within MIT or to any outside third parties and (ii) destroying identifiers at the earliest possible opportunity.

C. Will the health information you will receive for use in this study be de-identified?
☐ Yes X No ☐ N/A

The health information will all come directly from the respondent.

If yes, you do not need to obtain a signed Authorization for Release of Protected Health Information Form from study participants. Note, however, that if you receive identifiable participant health information that you plan to convert into de-identified information for use by other researchers, then you must obtain a signed Authorization for Release of Protected Health Information Form from each participant before receiving their identifiable health information for use in your study.

D. Will you be using or disclosing a limited data set?
☐ Yes X No

If yes and you will only receive participant health information in limited data set form, then you do not need to obtain a signed Authorization for Release of Protected Health Information Form from study participants. You must complete a formal data use agreement with the party from whom you will receive the limited data set information in order for your application to be approved.

If yes and you will receive identifiable participant health information that you plan to convert into limited data set form for use by other researchers, then you must obtain a signed Authorization for Release of Protected Health Information Form from each participant before receiving their identifiable health information for use in your study. You must complete a formal data use agreement in order for your application to be approved.

V. INVESTIGATOR'S ASSURANCE

I certify the information provided in this application is complete and correct.

I understand that I have ultimate responsibility for the conduct of the study, the ethical performance of the project, the protection of the rights and welfare of human subjects, and strict adherence to any stipulations imposed by COUHES

I agree to comply with all MIT policies, as well all federal, state and local laws on the protection of human subjects in research, including:

- **ensuring all study personnel satisfactorily complete human subjects training;**
- **performing the study according to the approved protocol;**
- **implementing no changes in the approved study without COUHES approval;**
- **obtaining informed consent from subjects using only the currently approved consent form;**
- **protecting identifiable health information, to the extent required by law, in accordance with HIPAA requirements; and**
- **promptly reporting significant or untoward adverse effects.**

Signature of Principal Investigator ______________________________ Date ___________

Print Full Name and Title ____

Signature of Department Head _________________________________ Date ___________

Print Full Name and Title ____

By signing this form, you confirm a scientific review of the proposed research has been conducted and that the proposed research is of scientific and scholarly validity.

Signed copies of the Comprehensive Review Application and supporting documents should be e-mailed to couhes@mit.edu. In addition, two single sided hardcopies must be submitted to the COUHES office: Building E25-Room 143b.

	Massachusetts Institute of Technology<br>Committee on the Use of<br>Humans as Experimental Subjects<br>COUHES	**COUHES Protocol #**<br>2003000118

PERSONNEL LIST

This form must be attached with the Application for Comprehensive Review. ***Any application submitted without a completed personnel list will be returned to you.***

Personnel is defined as anyone that plays a role in research involving human subjects, including direct contact, indirect involvement, analysis of data, blood or tissue samples. This extends to principal investigators, associate investigators, student investigators, study coordinators, visiting scientists, consultants, laboratory technicians and assistants.

All study personnel must be listed below. This listing must include contact information, a brief statement of qualifications and their study role.

Important note: all study personnel are required to complete Human Subject Training before work begins on the project.

I. MIT AFFILIATES

Personnel name and e-mail address	*Briefly describe qualifications*	*Study role(s)*	*Obtaining consent*
**Contact***<br>Name: Benjamin Olken<br>Email: bolken@mit.edu	Professor of Economics<br>Prior work on social messaging for public health	PI	X
Name: Esther Duflo<br>Email: eduflo@mit.edu	Professor of Economics	PI	☐
Name: Abhijit Banerjee<br>Email: banerjee@mit.edu	Professor of Economics	PI	☐
Name: Robert Dulin<br>Email: rdulin@povertyactionlab.org	Research Analyst		☐
Name: Pierre-Luc Vautrey<br>Email: vautrey@mit.edu	PhD student in Economics	Co-Investigator	X
Name: Anirudh Sankar<br>Email: asankar@povertyactionlab.org	Research Analyst		X
Name: Harsh Goyal<br>Email: hdgotal@mit.edu	MIT DEDP student<br>Research assistant		
Name: Ritesh Das<br>Email: dritesh@mit.edu	MIT DEDP student<br>Research assistant		

Name: Sirena Yu<br>Email: sirenayu@mit.edu	MIT Undergraduate Research Assistant (UROP)		X
Name: Mohit Karnani<br>Email: mohitkarnani@gmail.com	PhD student in Economics		X
Name: Advik Shreekumar<br>Email: adviks@mit.edu	PhD student in Economics		X

***NOTE:** Please designate a person with whom COUHES should communicate regarding issues or questions about the protocol.*

B. NON-MIT AFFILIATES

Proof of training must be attached for all non-MIT affiliates. Documentation from collaborating institutions may be submitted in lieu of training certificates.

Personnel name, affiliation, and e-mail address	*Briefly describe qualifications*	*Study role(s)*	*Obtaining consent*
Name: Marcella Alsan<br>Affiliation: Harvard<br>Email: marcella_alsan@hks.harvard.edu	MD, MPH, PhD<br>Professor of Public Policy	CO-PI<br>Will also vet all messaging to ensure consistency with CDC/public health authority guidelines	X
Name: Arun Chandrasekhar<br>Affiliation: Stanford<br>Email: arungc@stanford.edu	Assistant Professor of Economics<br>Prior work on social messaging for public health	Co-PI	X
Name: Emily Breza<br>Affiliation: Harvard<br>Email: ebreza@fas.harvard.edu.	Assistant Professor of Economics<br>Prior work on social networks	Co-PI	X
Name: Paul Goldsmith-Pinkham<br>Affiliation: Yale<br>Email: paul.goldsmith-pinkham@yale.edu	Assistant Professor of Finance<br>Prior work on social networks	Co-PI	X
Name: Fatima Cody Stanford<br>Affiliation: Massachusetts General Hospital/ Harvard Medical School<br>Email: FSTANFORD@mgh.harvard.edu	MD, MPH, MPA, FAAP, FACP, FAHA, FTOS	The MGH doctors are involved in the following activities:<br>- advising on message content<br>- recording video messaging	

		- advising on data analysis with deidentified data	
Name: Lucy Ogbu-Nwobodo<br>Affiliation: MGH/ Harvard Medical School<br>Email: logbu-nwobodo@mgh.harvard.edu	MD, MS<br>Resident, Clinical Fellow	The MGH doctors are involved in the following activities:<br>- advising on message content<br>- recording video messaging<br>advising on data analysis with deidentified data	
Name: Carlos Torres<br>Affiliation: MGH Chelsea HealthCare Center and MassGeneral Hospital for Children<br>Email: CTORRES4@PARTNERS.ORG	MD	The MGH doctors are involved in the following activities:<br>- advising on message content<br>- recording video messaging<br>advising on data analysis with deidentified data	
Name: Shobitha Cherian<br>Affiliation: J-PAL South Asia at IFMR<br><br>Email: shobitha.cherian@ifmr.ac.in	Prior involvement in education, diseases	Research Associate	X
Name: Tithee Mukhopadhyay<br>Affiliation: J-PAL South Asia at IFMR<br><br>Email: tithee.m@ifmr.ac.in	Prior involvement in social networks	Associate Director-Rsearch	X
Name: Vasu Chaudhary<br>Affiliation: J-PAL South Asia at IFMR<br>Email: vasu.chaudhary@ifmr.ac.	Prior involvement in education, diseases	Research Associate	X
Name: Sarah Eichmeyer<br>Affiliation: Stanford University<br>Email: saraeich@stanford.edu	PhD student in Economics		
