# Supplementary material for: Effect of Physician-Delivered COVID-19 Public Health Messages and Messages Acknowledging Racial Inequity on Black and White Adults’ Knowledge, Beliefs, and Practices Related to COVID-19: A Randomized Clinical Trial
Source: JAMA Netw Open. 2021 Jul 14;4(7):e2117115. doi: 10.1001/jamanetworkopen.2021.17115 (PMC8280971; doi:10.1001/jamanetworkopen.2021.17115)
Supplement: Supplement 3. — Nonauthor Contributors [file jamanetwopen-e2117115-s003.pdf]

Supplemental Online Content: Nonauthor Collaborators

*Indicates required information. Only first name, last name, and suffix will appear in PubMed.

***Group Name(s): COVID-19 Working Group**

***First Name and Middle Initial(s)**	***Last Name**	***Suffix (eg, Jr, III)**	Academic Degrees	Institution	Location (city, state/province, country)	Role or Contribution, eg, chair, principal investigator	Group (if more than 1 Group listed in the byline) and/or Subgroup (eg, Steering Committee)
Ingabire Grace	Balinda		M.D.	Massachusetts General Hospital	Boston, MA	Collaborator	
Richard	Bido-Medina		M.D., Ph.D.	Massachusetts General Hospital	Boston, MA	Collaborator	
Allison	Brandt		M.D.	Massachusetts General Hospital	Boston, MA	Collaborator	
Kemar	Brown		M.D.	Massachusetts General Hospital	Boston, MA	Collaborator	
Sherri-Ann	Burnett-Bowie		M.D., M.P.H.	Harvard Medical School	Boston, MA	Collaborator	
Lindsay Pindyck	Carter		M.D.	Massachusetts General Hospital	Boston, MA	Collaborator	
Jonathan	Chou		M.D.	Massachusetts General Hospital	Boston, MA	Collaborator	
Adriana	Cohen-Hauseman		M.D.	Massachusetts General Hospital	Boston, MA	Collaborator	
Kristen	Cotter		M.D., M.P.H.	Lynn Community Health Center	Lynn, MA	Collaborator	
Carine	Davila		M.D.	Massachusetts General Hospital	Boston, MA	Collaborator	
Paola	Daza		M.D.	Massachusetts General Hospital	Boston, MA	Collaborator	
Ariel	Frey-Vogel		M.D., M.A.T.	Massachusetts General Hospital	Boston, MA	Collaborator	
Linda	Galligani		N.P.	Lynn Community Health Center	Lynn, MA	Collaborator	
Wanda	Gonzalez		M.D.	Massachusetts General Hospital	Boston, MA	Collaborator	
Marita	Gove		N.P.	Lynn Community Health Center	Lynn, MA	Collaborator	
Daniel	Hall		M.D.	Massachusetts General Hospital	Boston, MA	Collaborator	
Kayla Theresa	Hartjes		M.D.	Massachusetts General Hospital	Boston, MA	Collaborator	
Jonathan	Hauseman		M.D.	Harvard Medical School	Boston, MA	Collaborator	
Linda	Herrera Santos		M.D., Ph.D.	Massachusetts General Hospital	Boston, MA	Collaborator	
Kelly John	Holland		M.D.	Lynn Community Health Center	Lynn, MA	Collaborator	
Katie	Hsih		M.D., MPhil	Massachusetts General Hospital	Boston, MA	Collaborator	

*Indicates required information. Only first name, last name, and suffix will appear in PubMed.

*First Name and Middle Initial(s)	*Last Name	*Suffix (eg, Jr, III)	Academic Degrees	Institution	Location (city, state/province, country)	Role or Contribution, eg, chair, principal investigator	Group (if more than 1 Group listed in the byline) and/or Subgroup (eg, Steering Committee)
Aisha	James		M.D., M.S.	Massachusetts General Hospital	Boston, MA	Collaborator	
Hannah	Janoowalla		M.D.	Lynn Community Health Center	Lynn, MA	Collaborator	
Gracia	Kwete		M.D.	Massachusetts General Hospital	Boston, MA	Collaborator	
Desta	Lissanu		M.D.	Massachusetts General Hospital	Boston, MA	Collaborator	
Merranda	Logan		M.D.	Massachusetts General Hospital	Boston, MA	Collaborator	
Lizbeeth	Lopez		M.D.	Massachusetts General Hospital	Boston, MA	Collaborator	
Wanda	Lopez-Rodriguez		M.D.	Massachusetts General Hospital	Boston, MA	Collaborator	
Njambi	Mathenge		M.D.	Massachusetts General Hospital	Boston, MA	Collaborator	
Juan	Matute		M.D.	Massachusetts General Hospital	Boston, MA	Collaborator	
George	Molina		M.D., M.P.H.	Brigham and Women's Hospital	Boston, MA	Collaborator	
Leah	Morelli		M.D.	Massachusetts General Hospital	Boston, MA	Collaborator	
Maeve	O'Neill		M.D.	Massachusetts General Hospital	Boston, MA	Collaborator	
Tawakalitu	Oseni		M.D.	Massachusetts General Hospital	Boston, MA	Collaborator	
Asishana	Osho		M.D., M.P.H.	Massachusetts General Hospital	Boston, MA	Collaborator	
Vashti	Otuya		D.O.	Lynn Community Health Center	Lynn, MA	Collaborator	
Numa	Perez		M.D.	Massachusetts General Hospital	Boston, MA	Collaborator	
Meryl	Perlman		M.D.	Massachusetts General Hospital	Boston, MA	Collaborator	
Rozanne	Puleo		N.P., M.S.	Lynn Community Health Center	Lynn, MA	Collaborator	
Daniela	Romero Crousillat		M.D.	Massachusetts General Hospital	Boston, MA	Collaborator	
Ana Maria	Rosales		M.D.	Massachusetts General Hospital	Boston, MA	Collaborator	
Shiva	Saboori		M.D.	Lynn Community Health Center	Lynn, MA	Collaborator	
Gloria	Salazar		M.D.	Massachusetts General Hospital	Boston, MA	Collaborator	
Shannon	Scott-Vernaglia		M.D.	Massachusetts General Hospital	Boston, MA	Collaborator	
Ashley Yih	Shaw		M.D., M.B.A.	Massachusetts General Hospital	Boston, MA	Collaborator	
Sahael	Stapleton		M.D.	Massachusetts General Hospital	Boston, MA	Collaborator	

Supplemental Online Content: Nonauthor Collaborators

*Indicates required information. Only first name, last name, and suffix will appear in PubMed.

***First Name and Middle Initial(s)**	***Last Name**	***Suffix (eg, Jr, III)**	Academic Degrees	Institution	Location (city, state/province, country)	Role or Contribution, eg, chair, principal investigator	Group (if more than 1 Group listed in the byline) and/or Subgroup (eg, Steering Committee)
Clark	Van Den Berghe		M.D.	Lynn Community Health Center	Lynn, MA	Collaborator	
Christopher	Velez		M.D.	Massachusetts General Hospital	Boston, MA	Collaborator	
