# Supplementary material for: Effect of Physician-Delivered COVID-19 Public Health Messages and Messages Acknowledging Racial Inequity on Black and White Adults’ Knowledge, Beliefs, and Practices Related to COVID-19: A Randomized Clinical Trial
Source: JAMA Netw Open. 2021 Jul 14;4(7):e2117115. doi: 10.1001/jamanetworkopen.2021.17115 (PMC8280971; doi:10.1001/jamanetworkopen.2021.17115)
Supplement: Supplement 4. — Data Sharing Statement [file jamanetwopen-e2117115-s004.pdf]

Data Sharing Statement

Torres. Effect of Physician-Delivered COVID-19 Public Health Messages and Messages Acknowledging Racial Inequity on Black and White Adults' Knowledge, Beliefs, and Practices Related to COVID-19. *JAMA Netw Open*. Published July 14, 2021. doi:10.1001/jamanetworkopen.2021.17115

Data
Data available: Yes
Data types: Deidentified participant data, Data dictionary, Other (please specify)
Additional Information: complete replication code and data is available at https://doi.org/10.7910/DVN/URX0UE
How to access data: complete replication code and data is available at https://doi.org/10.7910/DVN/URX0UE
When available: With publication

Supporting Documents
Document types: Statistical/analytic code, Informed consent form, Other (please specify)
Additional Information: IRB protocol
How to access documents: complete replication code and data is available at https://doi.org/10.7910/DVN/URX0UE. The protocol were also added to the submission package and can be made public on JAMA web page
When available: With publication

Additional Information
Who can access the data: anyone requesting the data
Types of analyses: any purpose
Mechanisms of data availability: with full documentation and without investigator support
